# Intense upconverted ultraviolet emission of Er^3+^ through confined energy transfer in Yb^3+^/Er^3+^ co-doped Rb_3_InCl_6_

**DOI:** 10.1038/s41467-025-58901-4

**Published:** 2025-07-22

**Authors:** Wen Zhang, Wei Zheng, Ping Huang, Dengfeng Yang, Zhiqing Shao, Wei Zhang, Hao Zhang, Zhi Xie, Jin Xu, Xueyuan Chen

**Affiliations:** 1 State Key Laboratory of Structural Chemistry and Fujian Key Laboratory of Nanomaterials, Fujian Institute of Research on the Structure of Matter, Chinese Academy of Sciences, Fuzhou, China; 2 Fujian Science & Technology Innovation Laboratory for Optoelectronic Information of China, Fuzhou, China; 3 University of Chinese Academy of Sciences, Beijing, China; 4 College of Mechanical and Electronic Engineering, Fujian Agriculture and Forestry University, Fuzhou, China

**Keywords:** Nanoparticles, Optical materials, Nonlinear optics

## Abstract

Yb^3+^/Er^3+^ activated upconversion (UC) materials have been widely applied in many advanced technologies owing to their high UC efficiency in the visible region. However, it is challenging to achieve efficient ultraviolet (UV) UC luminescence (UCL) in Yb^3+^/Er^3+^ system, due to the dense energy levels of Er^3+^ that impose deleterious nonradiative relaxation. Herein, we report a strategy to liberate the UV-UCL of Er^3+^ based on the confined energy transfer in Yb^3+^/Er^3+^ co-doped 0D Rb_3_InCl_6_ with a low phonon energy and a large interionic distance. This facilitates the population of Er^3+^ at the ^4^G_11/2_ state, which yields intense upconverted UV emission at 384 nm, with a much higher UV-to-green ratio (*I*_384_/*I*_554_ = 0.864) than that of traditional UC materials ( < 0.1). By leveraging the intense upconverted UV emission of Er^3+^, we demonstrate the application of Rb_3_InCl_6_:Yb^3+^/Er^3+^ nanocrystals as a NIR-to-UV transducer for NIR-triggered anion exchange of CsPbX_3_ perovskite nanocrystals with high efficiency and good controllability. These findings offer an approach for the exploration of novel UC materials via energy transfer and crystal lattice engineering towards versatile applications.

## Abstract (web summary)

Intense upconverted ultraviolet emission of Er^3+^ is achieved through confined energy transfer in Yb^3+^/Er^3+^ co-doped 0D Rb_3_InCl_6_, enabling NIR-driven anion exchange of CsPbX_3_ perovskite nanocrystals in haloalkanes with high controllability.

## Introduction

Lanthanide (Ln^3+^)-doped upconversion (UC) materials, owing to their ability of converting low-energy near-infrared (NIR) photons into high-energy ultraviolet (UV) and visible photons, have evoked tremendous interest in diverse fields covering from optoelectronics, photovoltaics and photocatalysis to biomedicine^1–4^. Specifically, the UV UC luminescence (UCL) of Ln^3+^ has great prospects in many frontier applications such as UV sterilization, cancer therapy, photochemical reaction, and aerospace, because of the high-energy and spatial resolution of UV light triggered by the NIR laser with good penetration depth, convenient remote controllability, and little photodamage to the targeted samples^5–7^. Generally, UV-UCL is achieved through a four- or five-photon energy transfer (ET) UC (ETU) process in Yb^3+^/Tm^3+^ co-doped system upon 980-nm excitation^8–12^. Compared with Yb^3+^/Tm^3+^, the Yb^3+^/Er^3+^ couple as a famous UC engine has been demonstrated to be more effective in UCL, typically in the green (≈540 nm) and red (≈650 nm) regions, due to the ladder-like energy levels of Er^3+^ that facilitate efficient ET from Yb^3+^ to Er^3+^ with minimal energy mismatch^13–15^. Specifically, the upconverted UV emission of Yb^3+^/Er^3+^ may offer a higher theoretical efficiency than that of Yb^3+^/Tm^3+^, as it can be generated from ^4^G_11/2_ (≈380 nm) of Er^3+^ through a three-photon ETU process under 980-nm excitation. However, it is challenging to realize efficient UV-UCL in Yb^3+^/Er^3+^ system, because of the dense energy levels of Er^3+^ that aggravate the nonradiative energy losses through cross relaxation between adjacent Er^3+^, back ET from Er^3+^ to Yb^3+^, and energy migration (EM) among Yb^3+^ to the surface and lattice defects^16,17^. In this context, it is imperative to develop a strategy to liberate the upconverted UV emission of Er^3+^, which is not only important for the NIR-to-UV utilization but also fundamentally significant for UC materials innovation based on ET engineering between Yb^3+^/Er^3+^ and other Ln^3+^ emitters.

To unlock the UV-UCL of Er^3+^, the search for new host materials with low phonon energies that can mitigate the nonradiative multiphonon relaxation (MPR) from ^4^G_11/2_ to ^2^H_9/2_ of Er^3+^ is of utmost importance^18,19^. In this regard, all-inorganic lead-free metal halides with cutoff phonon energies smaller than 300 cm^–1^ could be ideal candidates for this purpose. By doping with transition-metal, lanthanide, and/or main-group s-electron ions, a plethora of lead-free luminescent metal halides with excellent optical properties have been developed and explored as alternatives to lead halide perovskites for various optoelectronic applications^20–26^. Among these candidates, zero-dimensional (0D) In-based metal halides such as Rb_3_InCl_6_ stand out owing to their good structural stability, suitable bandgap and coordination environment for Ln^3+^ dopants, and spatially confined 0D structure that may alleviate the nonradiative energy losses associated with EM^27–29^. Note that the term “0D” herein is used to define the crystal structure with isolated structural units at the molecular level rather than the spherical nanoparticles with small sizes. These features make 0D Rb_3_InCl_6_ appealing as a distinctive host material for Ln^3+^ doping to achieve desirable UCL properties.

Herein, we report strong UV-UCL of Er^3+^ at 384 nm in Yb^3+^/Er^3+^ co-doped Rb_3_InCl_6_ microcrystals (MCs) and nanocrystals (NCs) under 980-nm excitation. The mechanism for the unusual upconverted UV emission of Er^3+^ in Rb_3_InCl_6_ is investigated in detail through the structural and spectroscopic analyses combined with the theoretical calculations as well as the comparison with traditional UC phosphors. By taking advantage of the intense upconverted UV emission of Er^3+^, we demonstrate the application of Rb_3_InCl_6_:Yb^3+^/Er^3+^ NCs as a UV generator for NIR-triggered anion exchange of CsPbX_3_ (X = Cl, Br, and I) perovskite NCs (PeNCs) in haloalkanes with high efficiency and remote controllability, thus unlocking the potential of Yb^3+^/Er^3+^ couple for NIR-to-UV utilization.

## Results

### Structural characterization of Rb_3_InCl_6_:Yb^3+^/Er^3+^

**Fig. 1:**
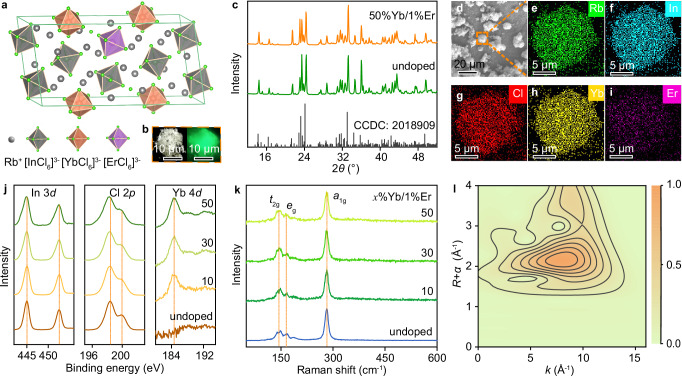
Structural characterization of Rb_3_InCl_6_:Yb^3+^/Er^3+^. **a** Schematic of the crystal structure of monoclinic Rb_3_InCl_6_ and the crystallographic site for Yb^3+^ and Er^3+^ dopants. **b** Photographs of Rb_3_InCl_6_: 50%Yb^3+^/1%Er^3+^ MCs under daylight (left) and 980-nm NIR laser irradiation (right). **c** Powder XRD patterns of undoped and 50 mol% Yb^3+^ and 1 mol% Er^3+^ co-doped Rb_3_InCl_6_ MCs. The bottom lines represent the standard XRD pattern of monoclinic Rb_3_InCl_6_ (CCDC No. 2018909). **d** SEM image and **e**–**i** EDS elemental mappings (Rb, In, Cl, Yb, and Er) of Rb_3_InCl_6_: 50%Yb^3+^/4%Er^3+^ MCs. **j** XPS and **k** Raman spectra of undoped Rb_3_InCl_6_ and Rb_3_InCl_6_: *x*%Yb^3+^/1%Er^3+^ MCs with different Yb^3+^ concentrations. **l** Contour plot of the EXAFS in *k*/*R*-space of Yb in Rb_3_InCl_6_: 50%Yb^3+^/1%Er^3+^ MCs.

Rb_3_InCl_6_ crystallizes with the Rb_3_YCl_6_ structure type (space group *C*2/*c*), which can be derived from the double perovskite structure by noncooperative tilting of isolated [InCl_6_]^3–^ octahedra^30^. Each [InCl_6_]^3–^ octahedron is surrounded by Rb^+^ cations, forming a spatially confined 0D structure at the molecular level (Fig. 1a). Yb^3+^ and Er^3+^ dopants are supposed to occupy the octahedral In^3+^ site with a symmetry of *C*_2h_^29^, resulting in bright UCL under 980 nm excitation (Fig. 1b). Yb^3+^ (or Er^3+^) singly-doped and Yb^3+^/Er^3+^ co-doped Rb_3_InCl_6_ MCs were synthesized via a solvothermal method by using InCl_3_, YbCl_3_, and ErCl_3_ as the metal precursors and methyl alcohol as the solvent. The doping concentrations of Yb^3+^ and Er^3+^ were controlled by varying the feeding ratios of the metal precursors between In, Yb, and Er, and identified by inductively coupled plasma-atomic emission spectroscopy (ICP-AES), showing the actual doping or alloying concentrations of Yb^3+^ and Er^3+^ ranging from 6.2 and 0.9 mol% to 36.9 and 5.9 mol%, respectively (Supplementary Tables 1 and 2), slightly lower than their feeding concentrations. For convenience, we used the feeding concentrations in the following discussion.

Powder XRD patterns of the MCs displayed intense diffraction peaks that can be well indexed into monoclinic Rb_3_InCl_6_ (CCDC No. 2018909) without any discernible impurities, revealing high crystallinity and phase purity of the resulting MCs (Fig. 1c). Moreover, the diffraction peaks of the MCs shifted towards smaller angles with an increase in Yb^3+^ or Er^3+^ concentration (Supplementary Figs. 1 and 2), indicating lattice expansion of the MCs, as confirmed by Rietveld refinement of the XRD patterns (Supplementary Figs. 3 and 4), whereby the cell volume of the MCs was calculated to increase from 2451.3 Å^3^ in undoped Rb_3_InCl_6_ to 2460.5 Å^3^ in Rb_3_InCl_6_: 50%Yb^3+^/1%Er^3+^ (Supplementary Tables 3, 4). Such dopant-induced lattice expansion suggests that Yb^3+^ and Er^3+^ have been successfully incorporated into Rb_3_InCl_6_ lattice and substituted the In^3+^ site bearing the smaller ionic radius (*r*_Yb3+_ = 0.86 Å, *r*_Er3+_ = 0.88 Å, *r*_In3+_ = 0.81 Å, CN = 6). This can be further verified by scanning electron microscopy (SEM), energy-dispersive X-ray spectroscopy (EDS), X-ray photoelectron spectroscopy (XPS), Raman spectroscopy, and extended X-ray absorption fine structure (EXAFS) analyses. SEM images showed that the MCs had an irregular morphology with a broad size distribution in the range of 5–20 μm (Fig. 1d), and EDS elemental mappings revealed the homogeneous distribution of the Yb^3+^ and Er^3+^ dopants in Rb_3_InCl_6_ lattice (Fig. 1e–i, Supplementary Table 5). XPS spectra exhibited typical Yb 4 *d* and Er 4 *d* signals in Yb^3+^/Er^3+^ co-doped Rb_3_InCl_6_ MCs, accompanied by a slight shift of the In 3 *d*, Rb 3 *d* and Cl 2*p* peaks towards lower energies as compared to those observed in undoped Rb_3_InCl_6_ (Fig. 1j, Supplementary Figs. 5 and 6). Raman spectra of the MCs displayed vibrational peaks at 142, 169, and 282 cm^–1^, corresponding to the *t*_2g_, *e*_g_, and *a*_1g_ vibrational modes of the [InCl_6_]^3–^ octahedra, respectively (Fig. 1k)^31^. The introduction of Yb^3+^ led to a decrease in intensities and a broadening in the bandwidth of the Raman peaks, due to the decreased content of [InCl_6_]^3–^ octahedra as well as the lattice distortion resulting from the substitution of [InCl_6_]^3–^ with [YbCl_6_]^3–^ octahedra. The EXAFS analysis showed that the average coordination number for Yb^3+^ is ≈6.2 in Rb_3_InCl_6_: 50%Yb^3+^/1%Er^3+^ MCs (Fig. 1l, Supplementary Fig. 7, Supplementary Table 6), which is close to the coordination number (CN = 6) of In^3+^ in Rb_3_InCl_6_ lattice. These observations confirmed that Yb^3+^ substituted the octahedral In^3+^ site in Rb_3_InCl_6_ lattice.

### UCL properties of Rb_3_InCl_6_:Yb^3+^/Er^3+^

**Fig. 2:**
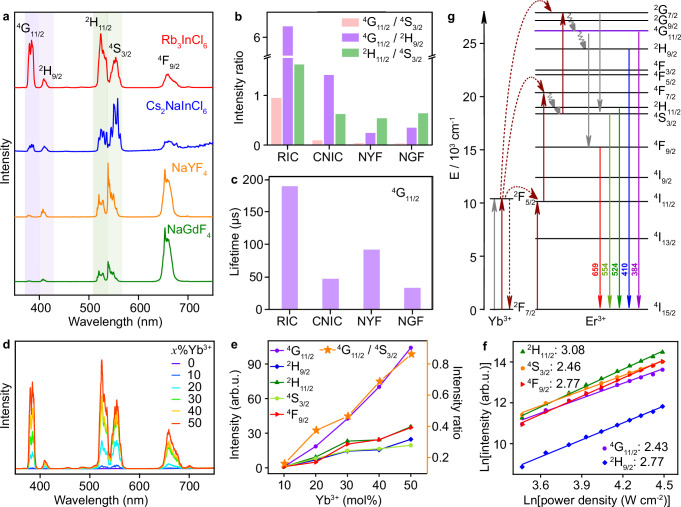
Intense upconverted UV emission of Er^3+^ in 0D Rb_3_InCl_6_:Yb^3+^/Er^3+^. **a** UCL spectra and **b** intensity ratios between the UV and green emissions from ^4^G_11/2_ and ^4^S_3/2_ of Er^3+^ (^4^G_11/2_/^4^S_3/2_) and between the emissions from the thermally coupled energy levels of ^4^G_11/2_/^2^H_9/2_ and ^2^H_11/2_/^4^S_3/2_ of Er^3+^ in Yb^3+^/Er^3+^ co-doped Rb_3_InCl_6_ (RIC), Cs_2_NaInCl_6_ (CNIC), NaYF_4_ (NYF), and NaGdF_4_ (NGF) MCs under excitation at 980 nm with a power density of 60 W cm^–2^. **c** UCL lifetimes of ^4^G_11/2_ of Er^3+^ (*λ*_em_: ≈384 nm) in Yb^3+^/Er^3+^ co-doped RIC, CNIC, NYF, and NGF. **d** UCL spectra of Rb_3_InCl_6_: *x*%Yb^3+^/1%Er^3+^ MCs with different Yb^3+^ concentrations under 980-nm excitation at a power density of 60 W cm^–2^. **e** UCL intensity ratio of ^4^G_11/2_/^4^S_3/2_ of Er^3+^ and intensities of the upconverted emissions from ^4^G_11/2_ (384 nm), ^2^H_9/2_ (409 nm), ^2^H_11/2_ (524 nm), ^4^S_3/2_ (554 nm), and ^4^F_9/2_ (659 nm) of Er^3+^ in Rb_3_InCl_6_: *x*%Yb^3+^/1%Er^3+^ MCs as a function of the Yb^3+^ concentration. **f** Power dependence of the UCL for the emissions from ^4^G_11/2_, ^2^H_9/2_, ^2^H_11/2_, ^4^S_3/2_, and ^4^F_9/2_ of Er^3+^ in Rb_3_InCl_6_: 50%Yb^3+^/1%Er^3+^ MCs. **g** Simplified energy-level scheme of Rb_3_InCl_6_:Yb^3+^/Er^3+^ MCs indicating the major UC processes. The colored solid lines and gray curves represent the radiative and nonradiative transitions, respectively.

Figure 2a presents the representative UCL spectrum of Rb_3_InCl_6_: 50%Yb^3+^/1%Er^3+^ MCs upon NIR excitation with a 980-nm diode laser. The MCs exhibited a set of sharp and intense emission peaks of Er^3+^ at 384 nm (^4^G_11/2_ → ^4^I_15/2_), 409 nm (^2^H_9/2_ → ^4^I_15/2_), 524 nm (^2^H_11/2_ → ^4^I_15/2_), 554 nm (^4^S_3/2_ → ^4^I_15/2_), and 659 nm (^4^F_9/2_ → ^4^I_15/2_). Strikingly, we found that the UV emission (384 nm) of Er^3+^ was unusually strong and comparable to the green emission (554 nm) under excitation with power densities in a wide range from 0.5 to 150 W cm^–2^ (Supplementary Fig. 8). The intensity ratio between the UV and green emissions of Er^3+^ (*I*_384_/*I*_554_) in Rb_3_InCl_6_: 50%Yb^3+^/1%Er^3+^ MCs was calculated to be 0.864 under 980-nm excitation at a power density of 60 W cm^–2^, which is about 1 − 2 orders of magnitude larger than those obtained in conventional UC phosphors such as NaYF_4_:Yb^3+^/Er^3+^ (0.025) and NaGdF_4_:Yb^3+^/Er^3+^ (0.027) and double perovskite Cs_2_NaInCl_6_:Yb^3+^/Er^3+^ MCs (0.083) (Fig. 2b, Supplementary Figs. 9–11). The corresponding UCL lifetime of ^4^G_11/2_ of Er^3+^ (190 μs) in Rb_3_InCl_6_:Yb^3+^/Er^3+^ was also much longer than those in NaYF_4_:Yb^3+^/Er^3+^ (57 μs), NaGdF_4_:Yb^3+^/Er^3+^ (18 μs), and Cs_2_NaInCl_6_:Yb^3+^/Er^3+^ (47 μs) (Fig. 2c, Supplementary Fig. 12), revealing the long-lived ^4^G_11/2_ state of Er^3+^ in Rb_3_InCl_6_:Yb^3+^/Er^3+^, which supports the intense UV emission. It is worth mentioning that, Cs_2_NaInCl_6_:Yb^3+^/Er^3+^ MCs exhibited much weaker UCL with a short decay time for ^4^G_11/2_ of Er^3+^ in comparison with that of Rb_3_InCl_6_:Yb^3+^/Er^3+^ MCs, due to the high symmetry of Yb^3+^ and Er^3+^ (*O*_h_) and the inefficient ETU processes from Yb^3+^ to Er^3+^ in Cs_2_NaInCl_6_:Yb^3+^/Er^3+^^32,33^, though they have similarly low phonon energies (Supplementary Fig. 13). Additionally, because of the low phonon energies (<282 cm^−1^) of Rb_3_InCl_6_ that alleviate the MPR processes, the intensity ratios between the emissions from the thermally coupled energy levels of ^4^G_11/2_/^2^H_9/2_ (*I*_384/409_) and ^2^H_11/2_/^4^S_3/2_ (*I*_524/554_) of Er^3+^ were significantly improved in Rb_3_InCl_6_:Yb^3+^/Er^3+^ in comparison with those in the other UC phosphors (Supplementary Table 7). The suppressed MPR of Ln^3+^ in Rb_3_InCl_6_ can also be evidenced by the negligibly weak UCL in Rb_3_InCl_6_:Yb^3+^/Tm^3+^ and Rb_3_InCl_6_:Yb^3+^/Ho^3+^ MCs (Supplementary Fig. 14), wherein the energy gap between ^3^H_5_ of Tm^3+^ (or ^5^I_6_ of Ho^3+^) and ^2^F_5/2_ of Yb^3+^ cannot be bridged by the lattice phonons. The intriguing UCL properties of Rb_3_InCl_6_:Yb^3+^/Er^3+^ MCs cannot be ascribed to the laser heating effect, as the temperature rise due to laser heating (11.3 °C, 60 W cm^–2^) is not high enough to cause a significant change in the population of Er^3+^ among different energy levels (Supplementary Figs. 15 and 16).

To gain deep insights into the unusual upconverted UV emission (^4^G_11/2_ → ^4^I_15/2_) of Er^3+^ in Rb_3_InCl_6_:Yb^3+^/Er^3+^ MCs, we investigated the effects of the Yb^3+^ concentration on the UCL properties of the MCs. As shown in Fig. 2d, the integrated UCL intensity of the MCs increased steadily with the increasing Yb^3+^ concentration from 10 to 50 mol%, indicating the absence of concentration quenching effect of Yb^3+^ in Rb_3_InCl_6_: *x*%Yb^3+^/1%Er^3+^ MCs. Higher doping concentrations of Yb^3+^ beyond 50 mol% led to the RbCl impurity. Concurrently, the intensity ratio of UV-to-green (*I*_384_/*I*_554_) of Er^3+^ was remarkably enhanced from 0.182 (10 mol% of Yb^3+^) to 0.864 (50 mol% of Yb^3+^) (Fig. 2e). This observation is in stark contrast to previous findings in other Yb^3+^/Er^3+^ co-doped UC phosphors such as NaYF_4_:Yb^3+^/Er^3+^, where the intensity ratio of ^4^G_11/2_/^4^S_3/2_ of Er^3+^ underwent a decrease with the increasing Yb^3+^ concentration, due to the enhanced back ET from Er^3+^ to Yb^3+^ (Er^3+^: ^4^G_11/2_ + Yb^3+^: ^2^F_7/2_ → Er^3+^: ^4^F_9/2_ + Yb^3+^: ^2^F_5/2_) at higher Yb^3+^ concentrations^34–36^. Correspondingly, the UCL lifetimes of Er^3+^ exhibited a decrease upon increasing the Yb^3+^ concentration (Supplementary Fig. 17, Supplementary Table 8), as a result of improved ETU processes that accelerated the exhaustion of the excitation energy from Yb^3+^ and consequently the depopulation of Er^3+^ from the emitting levels^37^.

Figure 2f shows the double logarithmic plots of the UCL intensities (*I*) of Rb_3_InCl_6_: 50%Yb^3+^/1%Er^3+^ MCs versus the excitation power density (*P*), whereby the number of pump photons required to populate each emitting level of Er^3+^ can be derived from the slope (*I* ∝ *P*^s^)^38^. By linear fitting to the plots, the slopes (*s*) for the upconverted emissions from ^4^G_11/2_, ^2^H_9/2_, ^2^H_11/2_, ^4^S_3/2_, and ^4^F_9/2_ of Er^3+^ were determined to be 2.43, 2.77, 3.08, 2.46, and 2.77, respectively. Similar nonlinear slopes were also obtained in Rb_3_InCl_6_: *x*%Yb^3+^/1%Er^3+^ MCs with different Yb^3+^ concentrations (Supplementary Fig. 18). According to Pollnau et al^38^., this may be interpreted as a three-photon process, wherein ETU appears to be the dominant mechanism. Generally, the upconverted green emissions from ^2^H_11/2_ and ^4^S_3/2_ of Er^3+^ (524 and 554 nm) in Yb^3+^/Er^3+^ co-doped systems are governed by a two-photon ETU process, characterized by a nonlinear slope *s* < 2^39–41^. Therefore, the observation of nonlinear slopes greater than 2 for the green emissions of Er^3+^ in Rb_3_InCl_6_:Yb^3+^/Er^3+^ indicates a significant contribution of three-photon ETU processes for the population of ^2^H_11/2_ and ^4^S_3/2_ of Er^3+^. As schematic illustration in Fig. 2g, the ^4^G_11/2_ and ^2^H_9/2_ levels of Er^3+^ are primarily populated through nonradiative relaxation from ^2^G_7/2_ with a three-photon ETU process, while the ^2^H_11/2_ and ^4^S_3/2_ levels can be populated through nonradiative relaxation from ^4^F_7/2_ with a two-photon ETU process. Concurrently, the excited Er^3+^ at ^2^G_7/2_ and ^4^G_11/2_ may also transfer the excitation energy to Yb^3+^, leaving themselves at ^2^H_11/2_/^4^S_3/2_ and ^4^F_9/2_, respectively^42^. The back ET from Er^3+^ to Yb^3+^ can be evidenced by the decreased intensity ratio of UV-to-green (*I*_384_/*I*_554_) of Er^3+^ and markedly reduced UCL lifetime of ^4^G_11/2_ with the increasing Er^3+^ concentration in Rb_3_InCl_6_: 50%Yb^3+^/*y*%Er^3+^ MCs (Supplementary Figs. 19 and 20, Supplementary Table 9). Such ET cycles between Yb^3+^ and Er^3+^ were also documented by Liu et al. in KYb_2_F_7_: Er^3+^ NCs, which led to the improved violet emission from ^2^H_9/2_ of Er^3+^ at 407 nm^43^. Moreover, owing to the low phonon energies (<282 cm^−1^) of the matrix, the MPR between the levels of Er^3+^ with energy gaps larger than 1400 cm^−1^ is inefficient in Rb_3_InCl_6_:Yb^3+^/Er^3+^, as a rule of thumb^44–46^. This property enables the high-order UCL of Rb_3_InCl_6_:Yb^3+^/Er^3+^, whereby the four-photon UCL from ^2^K_3/2_ (307 nm) and ^2^P_3/2_ (320 nm) of Er^3+^ can be explicitly observed (Supplementary Fig. 21). These results verify that the unusual upconverted UV emission of Er^3+^ in Rb_3_InCl_6_:Yb^3+^/Er^3+^ is associated with the low phonon energies of the matrix and the confined ET between Yb^3+^ and Er^3+^ in the peculiar 0D structure of Rb_3_InCl_6_.

### Confined ET between Yb^3+^ and Er^3+^ in 0D Rb_3_InCl_6_:Yb^3+^/Er^3+^

**Fig. 3:**
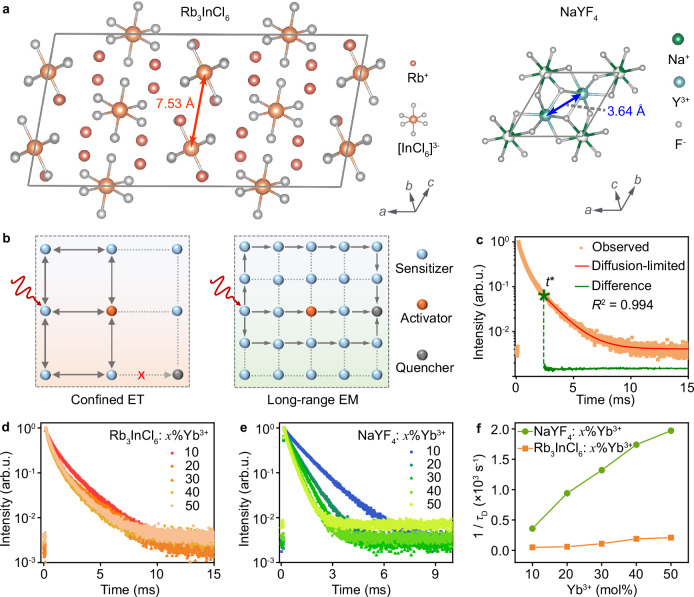
Confined ET between Yb^3+^ and Er^3+^ in 0D Rb_3_InCl_6_:Yb^3+^/Er^3+^. **a** Crystal structures of monoclinic Rb_3_InCl_6_ and hexagonal NaYF_4_ viewed along the *c* and *b* axes, respectively, showing the shortest In−In and Y−Y distances. **b** Schematic of the confined ET and long-range EM among the sensitizers and activators with long and short interionic distances, respectively. **c** PL decay curve of Yb^3+^ (*λ*_em_ = 994 nm) in Rb_3_InCl_6_: 50%Yb^3+^ MCs, which can be fitted well with the diffusion-limited mode. **d** PL decay curves of Yb^3+^ in d Rb_3_InCl_6_: *x*%Yb^3+^ and **e** NaYF_4_: *x*%Yb^3+^ MCs with different Yb^3+^ concentrations by monitoring the Yb^3+^ emission at 994 nm. **f** EM rates among Yb^3+^ in Rb_3_InCl_6_: *x*%Yb^3+^ and NaYF_4_: *x*%Yb^3+^ MCs with different Yb^3+^ concentrations.

To shed more light on the ET processes between Yb^3+^ and Er^3+^ in Rb_3_InCl_6_:Yb^3+^/Er^3+^ MCs, we calculated the interionic distance in Rb_3_InCl_6_: *x*%Yb^3+^/1%Er^3+^ MCs with different Yb^3+^ concentrations based on the density functional theory. As shown in Fig. 3a, the nearest distance between In^3+^ was determined to be 7.39 Å in Rb_3_InCl_6_, which is much longer than that between Y^3+^ (3.64 Å) in NaYF_4_. As a result, the Yb−Yb and Yb−Er distances (>7.14 Å) in Rb_3_InCl_6_:Yb^3+^/Er^3+^ are significantly larger than those (<3.50 Å) in NaYF_4_:Yb^3+^/Er^3+^ when Yb^3+^ and Er^3+^ substitute the In^3+^ or Y^3+^ sites in these two matrices (Supplementary Fig. 22)^36,47^. It is well known that the probability (or rate) of ET between Ln^3+^ ions depends strongly on their distance, and a short interionic distance favors the successive ET, namely, EM among identical ions (e.g., Yb^3+^), while an increase in the interionic distance will slow down the EM rate^48,49^. Regarding the large Yb−Yb and Yb−Er distances in Rb_3_InCl_6_:Yb^3+^/Er^3+^, we speculate that the long-range EM process among Yb^3+^ is suppressed, which accounts for the absence of concentration quenching effect of Yb^3+^ in this system. Instead, the excitation energy is deduced to be confined in the [YbCl_6_]^3–^ and [ErCl_6_]^3–^ octahedra within a short range, resulting in the ET cycles between Yb^3+^ and Er^3+^ (Fig. 3b).

The confined ET and suppressed EM processes in 0D Rb_3_InCl_6_:Yb^3+^/Er^3+^ can be further validated by measuring the photoluminescence (PL) decay curves of Rb_3_InCl_6_: *x*%Yb^3+^ MCs with different Yb^3+^ concentrations, whereby the rate of EM among Yb^3+^ can be derived on the basis of the energy diffusion model. According to G. Blasse et al.^50^, there are three modes of ET between Ln^3+^ ions when the back ET is negligible, namely, direct ET without diffusion, diffusion-limited, and fast-diffusion modes, which can be distinguished from the shapes of the decay curves of their excited states (Supplementary Fig. 23). In the case of direct ET without diffusion (*P*_DD_ = 0), the decay curve is characterized by an initially non-exponential portion which reflects the transfer to acceptors located at various distances from the donors, followed by an exponential portion with a decay rate equal to the radiative rate. In the case of fast diffusion, the rate of ET among donors (namely, EM) is much larger than that of ET to acceptors (*P*_DD_ > *P*_DA_), and the decay curve is exponential because the rapid migration has the effect of averaging the environments of the donors. In the case of diffusion-limited migration, the rate of ET among donors is lower than that from donors to acceptors (*P*_DD_ < *P*_DA_). In this situation, the decay curve of the donors is characterized by an initially fast non-exponential portion followed by a slow exponential portion with the decay time (*τ*) shorter than the radiative decay time (*τ*_0_) due to the influence of EM. The boundary between the non-exponential and exponential decay portions can be marked by a characteristic time t*. Based on these definitions, it can be deduced that the decay of ^2^F_5/2_ of Yb^3+^ in Rb_3_InCl_6_: *x*%Yb^3+^ MCs obeyed the diffusion-limited migration mode (Fig. 3c). According to M. Yokota and O. Tanimoto^51^, the decay rate due to migration (1/*τ*_D_) can thus be obtained by the following expression:

1 $$1/\tau=1/{\tau }_{D}+1/{\tau }_{0}$$

where 1/*τ* is the observed decay rate derived from the exponential portion of the decay curve and 1/*τ*_0_ is the radiative decay rate obtained from the low-doping (1 mol% Yb^3+^) sample. The results showed that the observed decay time (*τ*) of ^2^F_5/2_ of Yb^3+^ in Rb_3_InCl_6_: *x*%Yb^3+^ MCs exhibited only a slight decline from 1.84 to 1.43 ms as the Yb^3+^ concentration increased from 10 to 50 mol% (Fig. 3d, Supplementary Fig. 24), due to the long Yb−Yb distance that mitigated the nonradiative relaxation of Yb^3+^ via EM. This is in marked contrast to the observation in NaYF_4_: *x*%Yb^3+^ MCs, wherein the decay time (*τ*) of Yb^3+^ reduced drastically from 1.12 ms (10 mol%) to 0.37 ms (50 mol%) (Fig. 3e, Supplementary Fig. 25). Specifically, the EM rate (1/*τ*_D_) of Yb^3+^ in Rb_3_InCl_6_:Yb^3+^ MCs (2.10 × 10^2 ^s^−1^ for 50%Yb^3+^) was determined to be about one order of magnitude slower than that in NaYF_4_:Yb^3+^ MCs (2.17 × 10^3 ^s^−1^ for 50%Yb^3+^) (Fig. 3f and Supplementary Table 10), confirming the inhibition of EM in Rb_3_InCl_6_:Yb^3+^ MCs. Therefore, after co-doping with Er^3+^ ions, ET cycles occurred mainly between Yb^3+^ and Er^3+^ because Er^3+^ ions are prone to locate in close proximity to Yb^3+^ in Rb_3_InCl_6_:Yb^3+^/Er^3+^ (Supplementary Fig. 26), which led to the unusual upconverted UV emission of Er^3+^ in this system. It is worthy of mentioning that the “shell model” developed by F. Rabouw and A. Meijerink et al^52^. instead of the Yokota-Tanimoto model could be more appropriate for elucidating the dynamics of ET from Yb^3+^ to Er^3+^, considering the complex interplay between the donors and acceptors. Benefiting from the confined ET, we achieved a quantum yield (QY) of 0.12% for the upconverted UV emission (384 nm) of Er^3+^ in Rb_3_InCl_6_: 50%Yb^3+^/1%Er^3+^ MCs upon 980-nm excitation at a power density of 60 W cm^–2^, which is much higher than that of the benchmark NaYF_4_: 18%Yb^3+^/2%Er^3+^ MCs (0.04%), although the overall UCQY of Rb_3_InCl_6_:Yb^3+^/Er^3+^ (0.42%) is lower than that of NaYF_4_:Yb^3+^/Er^3+^ (1.38%).

### NIR-triggered anion exchange of CsPbX_3_ PeNCs

Furthermore, we synthesized Rb_3_InCl_6_:Yb^3+^/Er^3+^ NCs with different Yb^3+^ and Er^3+^ concentrations to investigate the effect of particle size on the upconverted UV emission of Er^3+^. Structural and compositional analyses through XRD, transmission electron microscopy (TEM), EDS, ICP-AES, XPS, and Raman spectra confirmed the pure phase and high crystallinity of the resulting Rb_3_InCl_6_:Yb^3+^/Er^3+^ NCs with particle sizes in the range of 48.8–52.8 nm (Supplementary Figs. 27–29, Supplementary Tables 11, 12). Similar to their MC counterparts, Rb_3_InCl_6_:Yb^3+^/Er^3+^ NCs displayed strong UV emission of Er^3+^ at 384 nm (^4^G_11/2_ → ^4^I_15/2_), with the UV-to-green ratio (*I*_384_/*I*_554_) of up to 0.702 for Rb_3_InCl_6_: 50%Yb^3+^/1%Er^3+^ NCs, upon 980-nm excitation at a power density of 60 W cm^−2^ (Supplementary Fig. 30). The corresponding UC QYs for the UV emission (≈384 nm) and overall emissions (360−720 nm) of Er^3+^ in Rb_3_InCl_6_: 50%Yb^3+^/1%Er^3+^ NCs were determined to be 0.08% and 0.37%, respectively, slightly lower than those of their MC counterparts (0.12% and 0.42%). Concentration-dependent UCL measurements showed the increased UCL intensity and *I*_384_/*I*_554_ ratio with the increasing Yb^3+^ concentration from 10 to 50 mol%, concurrent with the decreased UCL lifetimes (Supplementary Fig. 31, Supplementary Table 13), confirming the absence of concentration quenching effect of Yb^3+^ in Rb_3_InCl_6_:Yb^3+^/Er^3+^ NCs. Further increasing the Er^3+^ concentration on the basis of Rb_3_InCl_6_: 50%Yb^3+^/1%Er^3+^ NCs shortened the interionic distance between Yb^3+^ and Er^3+^, resulting in the enhanced back ET from Er^3+^ to Yb^3+^ and the decreased *I*_384_/*I*_554_ ratio of Er^3+^ with reduced UCL lifetime of ^4^G_11/2_ (Supplementary Figs. 32 and 33, Supplementary Table 14). These results are generally consistent with those observed in Rb_3_InCl_6_:Yb^3+^/Er^3+^ MCs, unraveling that the intense upconverted UV emission of Er^3+^ is the intrinsic properties of Rb_3_InCl_6_:Yb^3+^/Er^3+^ and independent of the particle size. Our findings may pave the way for exploring efficient UV-UCL from the Yb^3+^/Er^3+^ couple based on the host materials with low photon energies and large interionic distances, which favor the multiphoton UC processes with a long lifetime of ^4^G_11/2_ of Er^3+^.

**Fig. 4:**
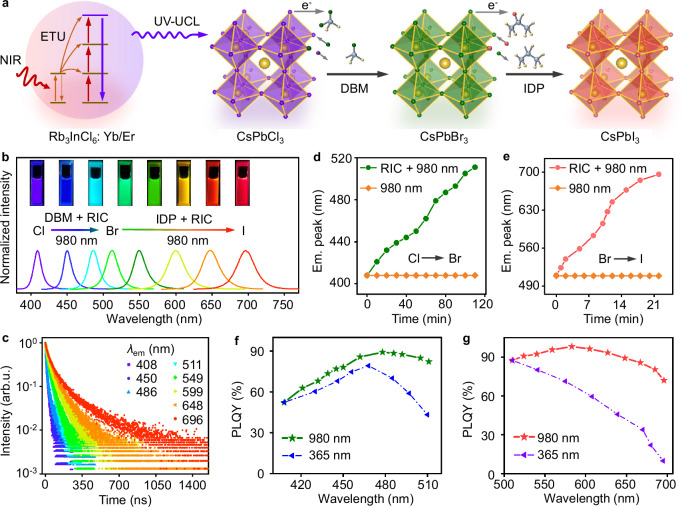
NIR-triggered anion exchange of CsPbX_3_ PeNCs using Rb_3_InCl_6_:Yb^3+^/Er^3+^. **a** Schematic of NIR-triggered anion exchange of CsPbX_3_ PeNCs in haloalkanes by using Rb_3_InCl_6_:Yb^3+^/Er^3+^ NCs as the NIR-to-UV transducer. **b** PL emission spectra (*λ*_ex_ = 365 nm) and **c** PL decay curves of CsPbX_3_ PeNCs with different halide compositions, derived from anion exchange based on the mixtures of RIC/DBM/CsPbCl_3_ and RIC/IDP/CsPbBr_3_ upon 980-nm NIR laser exposure. The insets in (**b**) show the PL photographs of the resulting PeNCs under 365-nm irradiation. Time dependence of the PL emission peaks (*λ*_ex_ = 365 nm) for **d** CsPbCl_3_ → CsPbBr_3_ in DBM/CsPbCl_3_ and RIC/DBM/CsPbCl_3_ and **e** CsPbBr_3_ → CsPbI_3_ in IDP/CsPbBr_3_ and RIC/IDP/CsPbBr_3_ upon 980-nm irradiation at a power density of 60 W cm^–2^. PLQYs for PeNCs derived from the photoinduced anion exchange **f** from CsPbCl_3_ to CsPbBr_3_ in DBM/CsPbCl_3_ (*λ*_ex_ = 365 nm) and RIC/DBM/CsPbCl_3_ (*λ*_ex_ = 980 nm) and **g** from CsPbBr_3_ to CsPbI_3_ in IDP/CsPbBr_3_ (*λ*_ex_ = 365 nm) and RIC/IDP/CsPbBr_3_ (*λ*_ex_ = 980 nm), respectively.

Considering the fact that the intense upconverted UV emission of Er^3+^ at 384 nm matches well with the absorption of CsPbX_3_ (X = Cl, Br, and I) PeNCs, we explored Rb_3_InCl_6_:Yb^3+^/Er^3+^ as a NIR-to-UV transducer for photoinduced anion exchange of CsPbX_3_ PeNCs in haloalkanes. CsPbX_3_ PeNCs are emerging as a new generation of semiconductor materials for various optoelectronic and photovoltaic applications owing their outstanding optical properties^53–55^. Upon 980-nm excitation, the upconverted UV emission of Er^3+^ from Rb_3_InCl_6_:Yb^3+^/Er^3+^ can be effectively absorbed by CsPbX_3_ to trigger the interfacial electron transfer from PeNCs to haloalkanes, resulting in the breakage of the covalent C–X bonds of haloalkanes with released halide ions for anion exchange with PeNCs (Fig. 4a)^56–58^. For this purpose, dibromomethane (DBM) and 2-iodopropane (IDP) were selected as the Br^−^ and I^−^ sources and mixed with cyclohexane (1:1 in volume), respectively. Then, CsPbCl_3_ and CsPbBr_3_ PeNCs were dispersed in the solvent of DBM and IDP, respectively, along with Rb_3_InCl_6_:Yb^3+^/Er^3+^ (RIC) NCs at the molar ratio of ≈1:1. Additionally, a trace amount of trioctylphosphine (≈20 μL) was added to improve the PL efficiency of the resulting CsPbX_3_ PeNCs^59,60^. The solution was then subjected to photoirradiation with a 980-nm diode laser, and the reaction was tracked by extracting aliquots of the reaction mixture at different time intervals, followed by the measurement of the optical absorption and PL spectra.

As expected, the mixtures of RIC/DBM/CsPbCl_3_ and RIC/IDP/CsPbBr_3_ underwent an obvious PL color change, respectively, from violet to blue and green and from green to yellow and red with the increasing irradiation time upon 980-nm exposure (Fig. 4b). For comparison, the emission color of the mixtures remained unchanged in the dark. The corresponding optical absorption and emission spectra of the PeNCs after NIR irradiation showed tunable bandgap (1.8–3.0 eV) and PL emission covering the entire visible spectral region from 408 to 696 nm (Supplementary Fig. 34), indicating the successful NIR-triggered halide exchange of CsPbX_3_ in haloalkanes. PL decay curves showed an increased PL lifetime from 9.8 to 84.0 ns, as the halide composition of PeNCs evolved from Cl^−^ to I^−^ with the increasing irradiation time (Fig. 4c), which is consistent with our previous findings^60^. Structural and morphological characterizations through XRD and TEM showed the cubic phase of PeNCs with mean sizes in the range of 18.0–21.9 nm (Supplementary Figs. 35 and 36), unveiling that the process of photoinduced anion exchange had no noticeable influence on the size and morphology of the resulting PeNCs.

The strategy of NIR-triggered anion exchange of CsPbX_3_ PeNCs provides significant advantages as compared to that using UV light directly, because it can not only avoid the photodamage of PeNCs upon prolonged UV exposure, but also offer a high degree of remote control by using the NIR laser as the excitation source. As shown in Fig. 4d and Supplementary Fig. 37, the emission peak of PeNCs derived from the mixture of RIC/DBM/CsPbCl_3_ experienced a continuous red-shift from 408 to 511 nm upon 980-nm excitation for 110 min, with PLQYs in the range of 52.3%–89.2% (Supplementary Table 15). Similarly, the emission peak of PeNCs derived from the mixture of RIC/IDP/CsPbBr_3_ shifted to red gradually from 510 to 696 nm upon 980-nm excitation for 22 min (Fig. 4e, Supplementary Fig. 38), with PLQYs in the range of 72.0%–98.1% (Supplementary Table 16). The faster anion exchange rate of CsPbBr_3_ in IDP than that of CsPbCl_3_ in DBM is ascribed to the weaker C–I bond than C–Br bond, which makes IDP more vulnerable to photoreduction^58^. By contrast, the emission peaks of PeNCs derived from DBM/CsPbCl_3_ and IDP/CsPbBr_3_ without RIC remained essentially unchanged upon 980-nm excitation for 3 h (Supplementary Fig. 39), underscoring the key role of NIR-to-UV UC processes in NIR-triggered anion exchange of CsPbX_3_ PeNCs. This feature enables the remote control of PeNCs with desired PL emissions by switching off the laser on demand at a specific exposure time (Supplementary Fig. 40). Moreover, the extent of halide exchange can also be controlled by tuning the power density of laser, whereby high-power excitation promoted the exchange rate (Supplementary Fig. 41). Note that the laser-induced heating effect may also contribute to the enhanced anion exchange rate due to the increased motion of the anions with the temperature rise (Supplementary Fig. 42), but it is not the cause of anion exchange as it cannot lead to the cleavage of the C–X bonds of DBM and IDP (Supplementary Fig. 43). For comparison, we also performed UV-triggered anion exchange of CsPbX_3_ PeNCs in haloalkanes without RIC NCs by using a 365-nm light-emitting diode (LED) as the excitation source under otherwise identical conditions. The results showed that 365-nm UV-LED can trigger the anion exchange of CsPbX_3_ PeNCs with an accelerated reaction rate (Supplementary Fig. 44), but it is detrimental to the PLQYs (decreased to 10.0% for CsPbI_3_) due to the serious photodamage of PeNCs upon direct UV exposure (Fig. 4f, g, Supplementary Tables 17, 18). Besides, although conventional UC materials such as NaYF_4_:Yb^3+^/Er^3+^ may also be applied for the NIR-triggered anion exchange of CsPbX_3_ PeNCs (Supplementary Fig. 45, Supplementary Tables 19 and 20), they are less efficient with a significantly reduced reaction rate, due to the much weaker UV-UCL than that of Rb_3_InCl_6_:Yb^3+^/Er^3+^. These results reveal the great potential of Rb_3_InCl_6_:Yb^3+^/Er^3+^ in NIR-to-UV utilization, and also offer a new way for the post-synthesis modification of PeNCs through NIR-triggered halide exchange towards various optoelectronic applications such as perovskite-based color conversion and patterning.

## Discussion

In summary, we have achieved abnormally strong UV-UCL of Er^3+^ in Yb^3+^/Er^3+^ co-doped 0D Rb_3_InCl_6_ under 980-nm excitation. Mechanistic investigation through concentration- and power-dependent UCL spectroscopic analyses unraveled that the unusual upconverted UV emission of Er^3+^ is dictated by the spatially confined 0D structure of Rb_3_InCl_6_ with a large interionic distance and low phonon energies, which facilitated the confined ET between Yb^3+^ and Er^3+^ within a short range while suppressing the energy losses through the MPR and long-range EM processes. This promoted the population of Er^3+^ at the ^4^G_11/2_ state, resulting in the intense UV emission at 384 nm with a large UV-to-green ratio (*I*_384_/*I*_554_) of 0.864 and a QY of 0.12% upon 980-nm excitation at a power density of 60 W cm^–2^, which are significantly higher than those of conventional UC materials. Furthermore, we demonstrated the application of Rb_3_InCl_6_:Yb^3+^/Er^3+^ NCs as an efficient NIR-to-UV transducer for NIR-triggered anion exchange of CsPbX_3_ PeNCs in haloalkanes with a high efficiency and remote controllability. These findings provide fundamental insights into the effect of ET and crystal lattice engineering on the UC dynamics of Ln^3+^, which lay a foundation for the UC materials innovation based on the confined ET between different Ln^3+^ emitters towards versatile applications such as NIR-to-UV utilization.

## Methods

### Materials

RbCl (99.99%), Rb_2_CO_3_ (99.8%), Cs_2_CO_3_ (99.99%), InCl_3_ (99.99%), In(CH_3_COO)_3_ (99.99%), YbCl_3_ 6H_2_O (99.99%), Yb(CH_3_COO)_3_ 4H_2_O (99.99%), Yb(NO_3_)_3_ 6H_2_O (99.9%), ErCl_3_ 6H_2_O (99.99%), Er(CH_3_COO)_3_ 4H_2_O (99.99%), Er(NO_3_)_3_ 6H_2_O (99.9%), TmCl_3_ 6H_2_O (99.99%), HoCl_3_ 6H_2_O (99.99%), Y(NO_3_)_3_ 6H_2_O (99.9%), Gd(NO_3_)_3_ 6H_2_O (99.9%), oleic acid (OA, 90%), oleylamine (OAm, 90%), 1-octadecene (ODE, 90%), and trioctylphosphine (TOP, 98%) were purchased from Sigma-Aldrich (Shanghai, China). CsCl (99.999%), Pb(CH_3_COO)_3_·3H_2_O (99.99%), benzoyl chloride (98.0%), isopropyl alcohol (99.9%), dibromomethane (DBM, 99%), and 2-iodopropane (IDP, 99%) were purchased from Aladdin (Shanghai, China). NaCl (99.5%), NaOH (96.0%), NaF (99.8%), hydrochloric acid (HCl, 37%), methanol (CH_3_OH, 99.9%), and ethanol (C_2_H_5_OH, 99.9%) were purchased from Sinopharm Chemical Reagent Co. (Shanghai, China). All chemical reagents were used as received without further purification.

### Synthesis of Rb_3_InCl_6_:Yb^3+^/Er^3+^ MCs

Yb^3+^ and Er^3+^ singly-doped and Yb^3+^/Er^3+^ co-doped Rb_3_InCl_6_ MCs were synthesized via a solvothermal method by using InCl_3_, YbCl_3_, and ErCl_3_ as the metal precursors and methanol as the solvent. In a typical synthesis of Rb_3_InCl_6_: 50%Yb^3+^/1%Er^3+^ MCs, 1.5 mmol of RbCl, 0.245 mmol of InCl_3_, 0.25 mmol of YbCl_3_ ⋅ 6H_2_O and 0.005 mmol of ErCl_3_ ⋅ 6H_2_O were dissolved in 8.0 mL of methanol in a 20 mL Teflon autoclave. Thereafter, the solution was heated at 180 °C for 12 h in a stainless-steel autoclave. The mixture was then cooled down to 30 °C at a speed of 3 °C h^−1^. Finally, the MCs were filtered out, washed three times with ethanol, and dried in an oven at 60 °C. For synthesizing Rb_3_In ⋅ Cl_6_: *x*%Yb^3+^/*y*%Er^3+^ MCs with different Yb^3+^ and Er^3+^ concentrations, (0.5 × *x*%) mmol of YbCl_3_ ⋅ 6H_2_O, (0.5 × *y*%) mmol of ErCl_3_ ⋅ 6H_2_O, and (0.5 × (1 − *x*% − *y*%)) mmol of InCl_3_ were used under otherwise identical conditions.

### Synthesis of Rb_3_InCl_6_:Yb^3+^/Er^3+^ NCs

Rb_3_InCl_6_:Yb^3+^/Er^3+^ NCs were synthesized through a modified hot-injection method by using benzoyl chloride as the halide source. In a typical process of Rb_3_InCl_6_: 50%Yb^3+^/1%Er^3+^ NCs, 0.45 mmol of Rb_2_CO_3_, 0.147 mmol of In(CH_3_COO)_3_·4H_2_O, 0.15 mmol of Yb(CH_3_COO)_3_·4H_2_O, and 0.003 mmol of Er(CH_3_COO)_3_·4H_2_O were added in a 100 mL two-neck flask containing 4 mL of OA, 1 mL of OAm, and 12 mL of ODE. The mixture was then heated to 120 °C under an N_2_ flow with constant stirring for 1 h to dissolve the powder and remove the moisture from the raw materials. After the solution became clear, the temperature was raised to 200 °C and stabilized for 10 min, followed by rapid injection of 1.8 mmol of benzoyl chloride into the hot solution. After 30 s of reaction, the mixture was cooled down to room temperature (RT) by an ice-water bath. The NCs were precipitated by centrifugation of the mixture at 4000 × *g* for 5 min, washed with 2 mL of cyclohexane, and collected by centrifugation again at 12,000 × *g* for 5 min. For synthesizing Rb_3_InCl_6_: *x*%Yb^3+^/*y*%Er^3+^ NCs with different Yb^3+^ and Er^3+^ concentrations, (0.3 × *x*%) mmol of Yb(CH_3_COO)_3_·4H_2_O, (0.3 × *y*%) mmol of Er(CH_3_COO)_3_·4H_2_O, and (0.3 × (1 − *x*% − *y*%)) mmol of In(CH_3_COO)_3_·4H_2_O were used under otherwise identical conditions.

### Synthesis of Cs_2_NaInCl_6_:Yb^3+^/Er^3+^ MCs

Yb^3+^/Er^3+^ co-doped Cs_2_NaInCl_6_ double perovskite MCs were synthesized via a modified solvothermal method. In a typical synthesis of Cs_2_NaInCl_6_: 50%Yb^3+^/1%Er^3+^ MCs, 0.8 mmol of CsCl, 0.4 mmol of NaCl, 0.4 mmol of In(CH_3_COO)_3_·4H_2_O, 0.2 mmol of Yb(CH_3_COO)_3_·4H_2_O, and 0.004 mmol of Er(CH_3_COO)_3_·4H_2_O were dissolved in 8.0 mL of HCl in a 20-mL Teflon autoclave. Thereafter, the solution was heated at 180 °C for 12 h in a stainless-steel autoclave. The solution was then cooled down to 30 °C at a speed of 3 °C h^−1^. Finally, the MCs were filtered out, washed three times with isopropyl alcohol, and dried in an oven at 60 °C.

### Synthesis of NaYF_4_:Yb^3+^/Er^3+^ and NaGdF_4_:Yb^3+^/Er^3+^ MCs

NaYF_4_: 18%Yb^3+^/2%Er^3+^ MCs were synthesized via a solvothermal method. In a typical synthesis, 0.6 g NaOH was dissolved in 9 mL of distilled water, and then 20 mL of OA and 9 mL of ethanol was added. After vigorous stirring for 30 min, 2 mL of aqueous solution containing 0.8 mmol of Y(NO_3_)_3_·6H_2_O, 0.18 mmol of Yb(NO_3_)_3_·6H_2_O, and 0.02 mmol of Er(NO_3_)_3_·6H_2_O was added, followed by addition of 4.0 mL of aqueous solution containing 4 mmol of NaF. The mixture was stirred for 30 min and then transferred to a 50 mL Teflon-lined autoclave and hydrothermally treated at 200 °C for 24 h. After the autoclave was cooled to RT, the precipitates were separated by centrifugation, washed with ethanol and distilled water for three times, and then dried at 60 °C. For synthesizing NaGdF_4_: 18%Yb^3+^/2%Er^3+^ MCs, Gd(CH_3_COO)_3_·6H_2_O instead of Y(CH_3_COO)_3_·6H_2_O was used under otherwise identical conditions.

### Synthesis of CsPbX_3_ PeNCs

CsPbX_3_ (X = Cl, Br, and I) PeNCs were synthesized via a modified hot-injection method by using HX as the halide source to precipitate the PeNCs. In a typical synthesis of CsPbCl_3_ PeNCs, 0.5 mmol of Pb(CH_3_COO)_3_·3H_2_O and 0.1 mmol of Cs_2_CO_3_ were mixed with 1 mL of OA, 1 mL of OAm, 1 mL of TOP, and 6 mL of ODE in a 50 mL three-neck round-bottom flask. The resulting mixture was heated to 120 °C under a N_2_ flow with constant stirring for 1 h to form a clear solution. The temperature was then raised up to 180 °C and stabilized for 10 min, followed by rapid injection of 1.5 mmol of HCl into the hot solution. After 10 s, the reaction mixture was cooled down to RT by an ice-water bath and centrifuged at 12,000 rpm for 5 min to collect the PeNCs. The precipitate was then dispersed in 1 mL of cyclohexane and centrifuged again at 12,000 × *g* for 5 min. After centrifugation, the supernatant was discarded and the PeNCs were redispersed in 30 mL of cyclohexane. Finally, 20 μL of TOP was added and ultrasonicated for 1 min to improve the PLQYs and long-term stability of the PeNCs. For synthesizing CsPbBr_3_ PeNCs, 1.5 mmol of HBr was injected into the hot solution under otherwise identical conditions.

### NIR-triggered anion exchange of CsPbX_3_ PeNCs

We demonstrated the NIR-triggered anion exchange of CsPbX_3_ PeNCs by using DBM and IDP as the Br^−^ and I^−^ sources, respectively. For the anion exchange of CsPbCl_3_ to CsPbBr_3_, 0.05 mmol of CsPbCl_3_ PeNCs and 0.05 mmol of Rb_3_InCl_6_: 50%Yb^3+^/1%Er^3+^ (RIC) NCs were dispersed in the mixed solution of DBM (1 mL) and cyclohexane (1 mL) with a volume of 1:1 in in a 3 mL quartz cuvette at RT. To improve the PL efficiency of the resulting CsPbX_3_ PeNCs, a trace amount of TOP (≈10 μL) was added. Then, the mixture was subjected to photoirradiation with a 980-nm diode laser, and the reaction was tracked by extracting aliquots of the reaction mixture at different time intervals, followed by the measurement of the optical absorption and PL spectra. It is worth mentioning that, we extracted the PeNCs with PL emission at 450 nm through centrifugation, which were used for further halide exchange to improve the exchange rate. For the anion exchange of CsPbBr_3_ to CsPbI_3_, 0.05 mmol of CsPbBr_3_ PeNCs and 0.05 mmol of Rb_3_InCl_6_: 50%Yb^3+^/1%Er^3+^ NCs were dispersed in the mixed solution of IDP (1 mL) and cyclohexane (1 mL) with a volume of 1:1. A trace amount of TOP (≈10 μL) was added to improve the PL efficiency of the PeNCs. Then, the mixture was subjected to photoirradiation with a 980-nm diode laser, and the reaction was tracked by extracting aliquots of the reaction mixture at different time intervals, followed by the measurement of the optical absorption and PL spectra.

#### Synchrotron X-ray absorption fine spectroscopy (XAFS) analyses

The XAFS analyses including the X-ray absorption near-edge structure (XANES) and EXAFS studies were conducted on the easy XAFS 300+ (energy 5–12 keV, Bragg Angle 55°–85°, resolution ratio 0.5–1.5 eV, luminous flux 7–9 keV) at Fujian Science & Technology Innovation Laboratory for Optoelectronic Information of China. The obtained XAFS data was processed in Athena (version 0.9.26) for background, pre-edge line, and post-edge line calibrations. Then, Fourier transformed fitting was carried out in Artemis. The *k*^2^ weighting, *k*-range of 2–10 Å^−2^, and *R* range of 1–2 Å were used to fit Rb_3_InCl_6_: 50%Yb^3+^/1%Er^3+^ MCs.

### DFT calculations

We employed the first principles to perform the DFT calculations within the generalized gradient approximation (GGA) using the Perdew-Burke-Ernzerhof (PBE) formulation. The projected augmented wave (PAW) potentials were used to describe the ionic cores and the valence electrons were taken into account using a plane wave basis set with a kinetic energy cutoff of 400 eV. Partial occupancies of the Kohn−Sham orbitals were allowed using the Gaussian smearing method with a width of 0.05 eV. The electronic energy was considered self-consistent when the energy change was smaller than 10^−6^ eV. A geometry optimization was considered convergent when the energy change was smaller than 0.03 eV Å^−1^. The Brillouin zone integration is performed using 1 × 1 × 1 Monkhorst-Pack *k*-point sampling for 3 × 3 × 2 supercell structure and 1 × 4 × 3 mesh for unit cell.

### Characterization

Powder XRD patterns were collected with an X-ray diffractometer (MiniFlex2, Rigaku) using Cu *K*_α1_ radiation (*λ* = 0.154187 nm). ICP-AES analyses were conducted on an ICP-AES spectrometer (Ultima2, Jobin Yvon). SEM measurements were performed by using a JSM-6700F SEM equipped with EDS. TEM measurements were performed by using a TECNAI G2 F20 TEM. XPS was carried out on a Thermo Fisher ESCALAB 250Xi using Al *K*_α_ (1486.6 eV) and He *I*_α_ (21.2 eV) as the sources of radiation. Raman spectra were recorded using a micro-Raman spectrometer (Labram HR Evolution, Horiba Scientific) upon laser excitation at 785 nm. Optical absorption spectra were translated from the UV-vis diffuse reflectance spectra, which were acquired with a PerkinElmer Lambda 950 UV/Vis/NIR spectrometer by using BaSO_4_ as a reference. UCL and PL spectra were measured on an FLS980 spectrometer (Edinburgh), and a 980-nm continuous-wave diode laser (2 W) was used the excitation source for the UCL measurements. UCL and PL lifetimes were measured on an FLS980 spectrometer (Edinburgh) equipped with a tunable midband Optical Parametric Oscillator (OPO) pulsed laser as the excitation source (410–2400 nm, 10 Hz, pulse width ≤5 ns, Vibrant 355II, OPOTEK). The absolute PL and UC QYs of the samples were obtained by employing a standard barium sulfate coated integrating sphere (150 nm in diameter, Edinburgh) as the sample chamber that was mounted on the FLS980 spectrometer with the entry and output port of the sphere located in 90° geometry from each other in the plane of the spectrometer. A standard tungsten lamp was used to correct the optical response of the instrument. UCL and PL photographs were taken by using a Huawei P30Pro cell phone with a 750 nm short-wave pass filter. All the spectral data were recorded at RT unless otherwise noted.

## Supplementary information

Supplementary Information

Transparent Peer Review file

## Data Availability

The data that support the findings of this study are available from the corresponding authors upon request.
