# Supplementary material for: Intense upconverted ultraviolet emission of Er3+ through confined energy transfer in Yb3+/Er3+ co-doped Rb3InCl6
Source: Nat Commun. 2025 Jul 22;16:6762. doi: 10.1038/s41467-025-58901-4 (PMC12283925; doi:10.1038/s41467-025-58901-4)
Supplement: Supplementary file 1 — Supplementary Information [file 41467_2025_58901_MOESM1_ESM.pdf]

# Supplementary Information

# Intense upconverted ultraviolet emission of $Er^{3+}$ through confined energy transfer in $Yb^{3+}/Er^{3+}$ co-doped $Rb_3InCl_6$

Wen Zhang,[1,2,3] Wei Zheng,[1,2,3,*] Ping Huang,[1,2,3,*] Dengfeng Yang,[1,3] Zhinqing Shao,[1,3] Wei Zhang,[1] Hao Zhang,[1] Zhi Xie,[4] Jin Xu,[1,2,3] and Xueyuan Chen[1,2,3,*]

[1]*State Key Laboratory of Structural Chemistry and Fujian Key Laboratory of Nanomaterials, Fujian Institute of Research on the Structure of Matter, Chinese Academy of Sciences, Fuzhou 350002, China.*

[2]*Fujian Science & Technology Innovation Laboratory for Optoelectronic Information of China, Fuzhou 350108, China.*

[3]*University of Chinese Academy of Sciences, Beijing 100049, China.*

[4]*College of Mechanical and Electronic Engineering, Fujian Agriculture and Forestry University, Fuzhou 350002, China.*

*To whom correspondence should be addressed, E-mail: zhengwei@fjirsm.ac.cn, huangping09@fjirsm.ac.cn, or xchen@fjirsm.ac.cn.

**Supplementary Table 1.** Nominal and actual doping concentrations of $Yb^{3+}$ in $Rb_3InCl_6$: $x\%Yb^{3+}/1\%Er^{3+}$ microcrystals (MCs). The nominal doping concentration of $Yb^{3+}$ was defined by the molar ratio of Yb to (Yb + In + Er) in the precursor solution, and the actual doping concentrations of $Yb^{3+}$ were identified by inductively coupled plasma-atomic emission spectroscopy (ICP-AES).

| Sample | In : Yb : Er | Nominal $Yb^{3+}$ (mol%) | Actual $Yb^{3+}$ (mol%) |
|---|---|---|---|
| 1 | 99 : 0 : 1 | 0 | 0 |
| 2 | 89 : 10 : 1 | 10 | 6.2 |
| 3 | 79 : 20 : 1 | 20 | 10.5 |
| 4 | 69 : 30 : 1 | 30 | 19.9 |
| 5 | 59 : 40 : 1 | 40 | 28.3 |
| 6 | 49 : 50 : 1 | 50 | 36.9 |

**Supplementary Table 2.** Nominal and actual doping concentrations of $Er^{3+}$ in $Rb_3InCl_6$: 50%$Yb^{3+}$/*y*%$Er^{3+}$ MCs. The nominal doping concentration of $Er^{3+}$ was defined by the molar ratio of Er to (Yb + In + Er) in the precursor solution, and the actual doping concentrations of $Er^{3+}$ were identified by ICP-AES.

| Sample | In : Yb : Er | Nominal | Actual |
|---|---|---|---|
| | | $Er^{3+}$ (mol%) | $Er^{3+}$ (mol%) |
| 1 | 49 : 50 : 1 | 1 | 0.9 |
| 2 | 48 : 50 : 2 | 2 | 1.9 |
| 3 | 47 : 50 : 3 | 3 | 2.6 |
| 4 | 46 : 50 : 4 | 4 | 3.5 |
| 5 | 45 : 50 : 5 | 5 | 4.3 |
| 6 | 44 : 50 : 6 | 6 | 5.1 |
| 7 | 43 : 50 : 7 | 7 | 5.9 |

**Supplementary Table 3.** Crystallographic data and details of the data collection and refinement parameters for $Rb_3InCl_6$ MCs. The goodness-of-fit parameter $\chi^2$ is smaller than 3.2, and the reliability factors $R_p$ and $R_{wp}$ are smaller than 7% and 10%, respectively, demonstrating the reliability of the refinement.

| Structural parameters | | Atomic parameters | | | |
|---|---|---|---|---|---|
| $2\theta$ range | $10° \leq 2\theta \leq 80°$ | Atom | $x$/a | $y$/b | $z$/c |
| Crystal system | monoclinic | In1 | 0.25 | -0.25 | 0.5 |
| Space group | $C2/c$ | In2 | 0.5 | 0.7835 | 0.25 |
| $a$ (Å) | 25.45 | Rb1 | 0.346 | 0.3153 | 0.3539 |
| $b$ (Å) | 7.78 | Rb2 | 0.4499 | 0.7499 | 0.566 |
| $c$ (Å) | 12.56 | Rb3 | 0.6625 | 0.8116 | 0.301 |
| $\alpha$ (°) | 90 | Cl1 | 0.2502 | -0.3841 | 0.3167 |
| $\beta$ (°) | 99.77 | Cl2 | 0.1731 | -0.0585 | 0.4212 |
| $\gamma$ (°) | 90 | Cl3 | 0.3189 | -0.0384 | 0.4643 |
| $V$ (Å$^3$) | 2451.3 | Cl4 | 0.5577 | 1.0215 | 0.1934 |
| $R_p$ (%) | 6.73 | Cl5 | 0.4433 | 0.7790 | 0.0688 |
| $R_{wp}$ (%) | 9.32 | Cl6 | 0.4427 | 0.5564 | 0.3169 |
| $\chi^2$ | 3.10 | | | | |

**Supplementary Table 4.** Crystallographic data and details of the data collection and refinement parameters for $Rb_3InCl_6$: 50%$Yb^{3+}$/1%$Er^{3+}$ MCs. The goodness-of-fit parameter $\chi^2$ is smaller than 2, and the reliability factors $R_p$ and $R_{wp}$ are smaller than 6% and 8%, respectively, demonstrating the reliability of the refinement.

| Structural parameters | | Atomic parameters | | | |
|---|---|---|---|---|---|
| $2\theta$ range | $10° \leq 2\theta \leq 80°$ | Atom | *x*/a | *y*/b | *z*/c |
| Crystal system | monoclinic | In1 | 0.25 | -0.25 | 0.5 |
| Space group | *C*2/*c* | In2 | 0.5 | 0.7835 | 0.25 |
| *a* (Å) | 25.46 | Rb1 | 0.346 | 0.3153 | 0.3539 |
| *b* (Å) | 7.80 | Rb2 | 0.4499 | 0.7499 | 0.566 |
| *c* (Å) | 12.56 | Rb3 | 0.6625 | 0.8116 | 0.301 |
| $\alpha$ (°) | 90 | Cl1 | 0.2502 | -0.3841 | 0.3167 |
| $\beta$ (°) | 99.76 | Cl2 | 0.1731 | -0.0585 | 0.4212 |
| $\gamma$ (°) | 90 | Cl3 | 0.3189 | -0.0384 | 0.4643 |
| *V* (Å$^3$) | 2460.5 | Cl4 | 0.5577 | 1.0215 | 0.1934 |
| $R_p$ (%) | 5.64 | Cl5 | 0.4433 | 0.7790 | 0.0688 |
| $R_{wp}$ (%) | 7.96 | Cl6 | 0.4427 | 0.5564 | 0.3169 |
| $\chi^2$ | 1.96 | | | | |

**Supplementary Table 5.** Elemental analyses of (1) $Rb_3InCl_6$, (2) $Rb_3InCl_6$: 50%$Yb^{3+}$, and (3) $Rb_3InCl_6$: 50%$Yb^{3+}$/1%$Er^{3+}$ MCs by X-ray photoelectron spectroscopy (XPS) and energy-dispersive X-ray spectroscopy (EDS).

| Measurement | Sample | Molar ratio[a)] (%) | | | | |
|---|---|---|---|---|---|---|
| | | Rb | In | Yb | Er | Cl |
| XPS | 1 | 314.1 | 100.0 | - | - | 572.7 |
| | 2 | 289.6 | 70.6 | 29.4 | - | 589.2 |
| | 3 | 302.3 | 69.8 | 29.5 | 0.7 | 576.6 |
| EDS | 1 | 298.2 | 100.0 | - | - | 612.8 |
| | 2 | 292.5 | 63.4 | 36.6 | - | 610.0 |
| | 3 | 278.8 | 65.6 | 34.4 | -[b)] | 574.1 |

a) The molar ratio was normalized so that $n(In^{3+}) + n(Yb^{3+}) + n(Er^{3+}) = 100\%$. The molar ratio of Rb was therefore calculated by $n(Rb^{+})/[n(In^{3+}) + n(Yb^{3+}) + n(Er^{3+})]$, and the molar ratio of Cl was calculated by $n(Cl^{-})/[n(In^{3+}) + n(Yb^{3+}) + n(Er^{3+})]$.

b) $Er^{3+}$ was not detected by EDS due to its low doping level (1 mol%).

**Supplementary Table 6.** Extended X-ray absorption fine structure (EXAFS) fitting parameters at the Yb L-edge in $Rb_3InCl_6$: 50%$Yb^{3+}$ MCs.

| shell | CN[a] | $R$ (Å)[b] | $\sigma^2$ (Å$^2$)[c] | $\Delta E_0$ (eV)[d] | $R$ factor[e] |
|---|---|---|---|---|---|
| Yb-Cl | 6.2 ± 0.6 | 2.60 ± 0.01 | 0.0020 | 2.6 ± 0.9 | 0.0088 |

a) CN: coordination numbers; b) $R$: bond distance; c) $\sigma^2$: Debye-Waller factors; d) $\Delta E_0$: the inner-potential correction; e) $R$ factor: goodness of fit. Error bounds that characterize the structural parameters obtained by EXAFS spectroscopy were estimated as CN ± 20%, $R$ ± 1%, and $\sigma^2$ ± 20%.

**Supplementary Table 7.** Upconversion luminescence (UCL) intensity ratios between the emissions from the thermally coupled energy levels of $^4G_{11/2}/^2H_{9/2}$ and $^2H_{11/2}/^4S_{3/2}$ of $Er^{3+}$ in $Rb_3InCl_6$: 50%$Yb^{3+}$/1%$Er^{3+}$, $Cs_2NaInCl_6$: 50%$Yb^{3+}$/1%$Er^{3+}$, $NaYF_4$: 18%$Yb^{3+}$/2%$Er^{3+}$, and $NaGdF_4$: 18%$Yb^{3+}$/2%$Er^{3+}$ MCs under excitation at 980 nm with a power density of 60 W $cm^{-2}$.

| Sample | Intensity ratio | |
|---|---|---|
| | $^4G_{11/2}/^2H_{9/2}$ | $^2H_{11/2}/^4S_{3/2}$ |
| $Rb_3InCl_6$: 50%$Yb^{3+}$/1%$Er^{3+}$ | 6.24 | 1.48 |
| $Cs_2NaInCl_6$: 50%$Yb^{3+}$/1%$Er^{3+}$ | 1.28 | 0.57 |
| $NaYF_4$: 18%$Yb^{3+}$/2%$Er^{3+}$ | 0.20 | 0.28 |
| $NaGdF_4$: 18%$Yb^{3+}$/2%$Er^{3+}$ | 0.36 | 0.48 |

**Supplementary Table 8.** Effective UCL lifetimes of $Er^{3+}$ in $Rb_3InCl_6$: $x$%$Yb^{3+}$/1%$Er^{3+}$ MCs with different $Yb^{3+}$ concentrations. The effective UCL lifetimes ($\tau_{eff}$) of the MCs were measured by monitoring the $Er^{3+}$ emissions at 384 nm ($^4G_{11/2}$), 524 nm ($^2H_{11/2}$), 554 nm ($^4S_{3/2}$), and 659 nm ($^4F_{9/2}$), respectively, upon 980-nm pulsed laser excitation, and calculated by

$$\tau_{\mathrm{eff}} = \frac{1}{I_{\mathrm{max}}}\int_0^{\infty} I(t)\, d\mathrm{t}$$

where $I(t)$ denotes the UCL intensity as a function of time $t$, and $I_{max}$ is the maximum UCL intensity.

| $Yb^{3+}$ (mol%) | $^4G_{11/2}$ (μs) | $^2H_{11/2}$ (μs) | $^4S_{3/2}$ (μs) | $^4F_{9/2}$ (μs) |
|---|---|---|---|---|
| 10 | 311 | 1000 | 975 | 1580 |
| 20 | 302 | 998 | 966 | 1514 |
| 30 | 259 | 848 | 763 | 1566 |
| 40 | 215 | 760 | 684 | 1450 |
| 50 | 190 | 689 | 609 | 1195 |

**Supplementary Table 9.** Effective UCL lifetimes of $Er^{3+}$ in $Rb_3InCl_6$: 50%$Yb^{3+}$/*y*%$Er^{3+}$ MCs with different $Er^{3+}$ concentrations. The effective UCL lifetimes ($\tau_{eff}$) of the MCs were measured by monitoring the $Er^{3+}$ emissions at 384 nm ($^4G_{11/2}$), 524 nm ($^2H_{11/2}$), 554 nm ($^4S_{3/2}$), and 659 nm ($^4F_{9/2}$), respectively, upon 980-nm pulsed laser excitation, and calculated by

$$\tau_{\text{eff}} = \frac{1}{I_{\text{max}}}\int_0^{\infty} I(t)\,dt$$

where $I(t)$ denotes the UCL intensity as a function of time $t$, and $I_{max}$ is the maximum UCL intensity.

| $Er^{3+}$ (mol%) | $^4G_{11/2}$ (μs) | $^2H_{11/2}$ (μs) | $^4S_{3/2}$ (μs) | $^4F_{9/2}$ (μs) |
|---|---|---|---|---|
| 1 | 190 | 689 | 609 | 1195 |
| 2 | 178 | 570 | 569 | 936 |
| 3 | 164 | 520 | 515 | 965 |
| 4 | 150 | 470 | 475 | 876 |
| 5 | 142 | 522 | 518 | 1094 |
| 6 | 158 | 599 | 593 | 1133 |
| 7 | 141 | 588 | 579 | 1174 |

**Supplementary Table 10.** The observed decay times and energy migration (or diffusion) rates of $Yb^{3+}$ in $Rb_3InCl_6$: *x*%$Yb^{3+}$ and $NaYF_4$: *x*%$Yb^{3+}$ MCs with different $Yb^{3+}$ concentrations. The observed decay times ($\tau$) of the MCs were measured by monitoring the $Yb^{3+}$ emission at 994 nm ($^2F_{5/2}$) upon 930-nm pulsed laser excitation and derived from the exponential portion of the decay curves, while the energy migration rates ($1/\tau_D$) were calculated by

$$1/\tau_D = 1/\tau - 1/\tau_0$$

where $1/\tau_0$ is the radiative decay rate obtained from the low-doping (1 mol% $Yb^{3+}$) sample.

| $Yb^{3+}$ (mol%) | $Rb_3InCl_6$: *x*%$Yb^{3+}$ | | $NaYF_4$: *x*%$Yb^{3+}$ | |
|---|---|---|---|---|
| | $\tau$ (ms) | $1/\tau_D$ ($\times 10^3$ $s^{-1}$) | $\tau$ (ms) | $1/\tau_D$ ($\times 10^3$ $s^{-1}$) |
| 1 | 2.03 ($\tau_0$) | - | 1.88 ($\tau_0$) | - |
| 10 | 1.84 | 0.05 | 1.12 | 0.36 |
| 20 | 1.80 | 0.06 | 0.67 | 0.96 |
| 30 | 1.65 | 0.11 | 0.54 | 1.32 |
| 40 | 1.46 | 0.19 | 0.41 | 1.91 |
| 50 | 1.43 | 0.21 | 0.37 | 2.17 |

**Supplementary Table 11.** Nominal and actual doping concentrations of $Yb^{3+}$ in $Rb_3InCl_6$: $x$%$Yb^{3+}$/1%$Er^{3+}$ nanocrystals (NCs). The nominal doping concentration of $Yb^{3+}$ was defined by the molar ratio of Yb to (Yb + In + Er) in the precursor solution, and the actual doping concentrations of $Yb^{3+}$ were identified by ICP-AES.

| Sample | In : Yb : Er | Nominal | Actual |
|---|---|---|---|
| | | $Yb^{3+}$ (mol%) | $Yb^{3+}$ (mol%) |
| 1 | 89 : 10 : 1 | 10 | 2.5 |
| 2 | 69 : 30 : 1 | 30 | 18.3 |
| 3 | 49 : 50 : 1 | 50 | 43.2 |

**Supplementary Table 12.** Nominal and actual doping concentrations of $Er^{3+}$ in $Rb_3InCl_6$: 50%$Yb^{3+}$/*y*%$Er^{3+}$ NCs. The nominal doping concentration of $Er^{3+}$ was defined by the molar ratio of Er to (Yb + In + Er) in the precursor solution, and the actual doping concentrations of $Er^{3+}$ were identified by ICP-AES.

| Sample | In : Yb : Er | Nominal | Actual |
|---|---|---|---|
| | | $Er^{3+}$ (mol%) | $Er^{3+}$ (mol%) |
| 1 | 49 : 50 : 1 | 1 | 0.8 |
| 2 | 46 : 50 : 4 | 4 | 3.6 |
| 3 | 43 : 50 : 7 | 7 | 5.3 |

**Supplementary Table 13.** Effective UCL lifetimes of $Er^{3+}$ in $Rb_3InCl_6$: $x$%$Yb^{3+}$/1%$Er^{3+}$ NCs with different $Yb^{3+}$ concentrations. The effective UCL lifetimes ($\tau_{eff}$) of the NCs were measured by monitoring the $Er^{3+}$ emissions at 384 nm ($^4G_{11/2}$), 524 nm ($^2H_{11/2}$), 554 nm ($^4S_{3/2}$), and 659 nm ($^4F_{9/2}$), respectively, upon 980-nm pulsed laser excitation, and calculated by

$$\tau_{\text{eff}} = \frac{1}{I_{\text{max}}} \int_0^{\infty} I(t)\, d\text{t}$$

where $I(t)$ denotes the UCL intensity as a function of time $t$, and $I_{max}$ is the maximum UCL intensity.

| $Yb^{3+}$ (mol%) | $^4G_{11/2}$ (μs) | $^2H_{11/2}$ (μs) | $^4S_{3/2}$ (μs) | $^4F_{9/2}$ (μs) |
|---|---|---|---|---|
| 10 | 190 | 662 | 693 | 869 |
| 30 | 76 | 273 | 240 | 347 |
| 50 | 59 | 157 | 151 | 207 |

**Supplementary Table 14.** Effective UCL lifetimes of $Er^{3+}$ in $Rb_3InCl_6$: 50%$Yb^{3+}$/*y*%$Er^{3+}$ NCs with different $Er^{3+}$ concentrations. The effective UCL lifetimes ($\tau_{eff}$) of the NCs were measured by monitoring the $Er^{3+}$ emissions at 384 nm ($^4G_{11/2}$), 524 nm ($^2H_{11/2}$), 554 nm ($^4S_{3/2}$), and 659 nm ($^4F_{9/2}$), respectively, upon 980-nm pulsed laser excitation, and calculated by

$$\tau_{\mathrm{eff}} = \frac{1}{I_{\mathrm{max}}} \int_0^{\infty} I(t)\, d\mathrm{t}$$

where $I(t)$ denotes the UCL intensity as a function of time $t$, and $I_{max}$ is the maximum UCL intensity.

| $Er^{3+}$ (mol%) | $^4G_{11/2}$ (μs) | $^2H_{11/2}$ (μs) | $^4S_{3/2}$ (μs) | $^4F_{9/2}$ (μs) |
|---|---|---|---|---|
| 1 | 59 | 157 | 151 | 207 |
| 4 | 44 | 109 | 99 | 192 |
| 7 | 33 | 87 | 78 | 167 |

**Supplementary Table 15.** Time dependence of the PL emission peak ($\lambda_{em}$) and PL quantum yield (PLQY) for PeNCs derived from the mixture of RIC/DBM/$CsPbCl_3$ upon 980-nm irradiation at a power density of 60 W $cm^{-2}$.

| Time (min) | $\lambda_{em}$ (nm) | PLQY (%) |
|---|---|---|
| 0 | 408 | 52.3 |
| 10 | 421 | 62.8 |
| 20 | 432 | 68.0 |
| 30 | 439 | 73.5 |
| 40 | 445 | 76.9 |
| 50 | 450 | 78.1 |
| 60 | 462 | 85.7 |
| 70 | 478 | 89.2 |
| 80 | 486 | 88.4 |
| 90 | 492 | 87.6 |
| 100 | 505 | 84.9 |
| 110 | 511 | 82.2 |

**Supplementary Table 16.** Time dependence of the PL emission peak ($\lambda_{em}$) and PLQY for PeNCs dervied from the mixture of RIC/IDP/$CsPbBr_3$ upon 980-nm irradiation at a power density of 60 W $cm^{-2}$.

| Time (min) | $\lambda_{em}$ (nm) | PLQY (%) |
|---|---|---|
| 0 | 510 | 87.5 |
| 1 | 524 | 90.7 |
| 2 | 540 | 92.3 |
| 5 | 559 | 95.6 |
| 8 | 583 | 98.1 |
| 10 | 605 | 96.3 |
| 11 | 627 | 93.4 |
| 12 | 645 | 89.2 |
| 15 | 667 | 85.7 |
| 18 | 685 | 80.4 |
| 22 | 696 | 72.0 |

**Supplementary Table 17.** Time dependence of the PL emission peak ($\lambda_{em}$) and PLQY for PeNCs dervied from the mixture of DBM/$CsPbCl_3$ upon 365-nm UV-LED (3 W) irradiation.

| Time (min) | $\lambda_{em}$ (nm) | PLQY (%) |
|---|---|---|
| 0 | 406 | 50.8 |
| 1 | 430 | 60.3 |
| 2 | 446 | 68.0 |
| 3 | 456 | 74.7 |
| 5 | 468 | 79.2 |
| 8 | 485 | 70.1 |
| 12 | 497 | 58.9 |
| 17 | 510 | 43.2 |

**Supplementary Table 18.** Time dependence of the PL emission peak ($\lambda_{em}$) and PLQY for PeNCs dervied from the mixture of IDP/$CsPbBr_3$ upon 365-nm UV-LED (3 W) irradiation.

| Time (min) | $\lambda_{em}$ (nm) | PLQY (%) |
|---|---|---|
| 0 | 512 | 88.7 |
| 1 | 541 | 80.1 |
| 2 | 575 | 71.3 |
| 3 | 608 | 59.5 |
| 5 | 639 | 45.8 |
| 6 | 670 | 33.9 |
| 7 | 680 | 22.1 |
| 8 | 695 | 10.0 |

**Supplementary Table 19.** Time dependence of the PL emission peak ($\lambda_{em}$) and PLQY for PeNCs derived from the mixture of $NaYF_4$: $Yb^{3+}/Er^{3+}$ (NYF), DBM, and $CsPbCl_3$ (NYF/DBM/$CsPbCl_3$) upon 980-nm irradiation at a power density of 60 W $cm^{-2}$.

| Time (min) | $\lambda_{em}$ (nm) | PLQY (%) |
|---|---|---|
| 0 | 406 | 50.8 |
| 10 | 428 | 62.5 |
| 25 | 441 | 69.1 |
| 40 | 457 | 72.6 |
| 65 | 468 | 79.2 |
| 90 | 487 | 86.1 |
| 120 | 499 | 73.9 |
| 150 | 515 | 59.1 |

**Supplementary Table 20.** Time dependence of the PL emission peak ($\lambda_{em}$) and PLQY for PeNCs derived from the mixture of $NaYF_4$: $Yb^{3+}/Er^{3+}$, IDP, and $CsPbBr_3$ (NYF/IDP/$CsPbBr_3$) upon 980-nm irradiation at a power density of 60 W cm$^{-2}$.

| Time (min) | $\lambda_{em}$ (nm) | PLQY (%) |
|---|---|---|
| 0 | 512 | 88.7 |
| 2 | 538 | 90.1 |
| 5 | 570 | 93.3 |
| 10 | 601 | 95.5 |
| 15 | 630 | 87.9 |
| 20 | 665 | 79.6 |
| 25 | 683 | 70.4 |
| 32 | 698 | 56.2 |

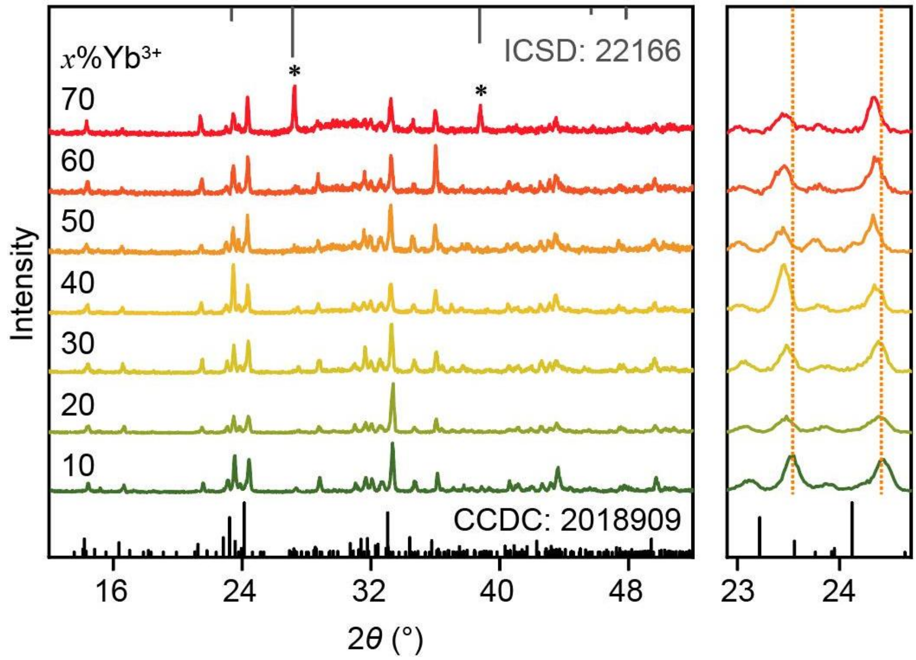


**Supplementary Figure 1.** XRD patterns of $Rb_3InCl_6$: $x$%$Yb^{3+}$/1%$Er^{3+}$ MCs with different $Yb^{3+}$ concentrations. The bottom and top lines represent the standard XRD patterns of monoclinic $Rb_3InCl_6$ (CCDC No. 2018909) and cubic RbCl (ICSD No. 22166), respectively. All diffraction peaks of the MCs ($x$ = 10−50) can be well indexed into monoclinic $Rb_3InCl_6$, indicating high crystallinity and phase purity of the as-synthesized $Rb_3InCl_6$: $x$%$Yb^{3+}$/1%$Er^{3+}$ MCs. The diffraction peaks of the MCs shifted towards lower angles with the increasing $Yb^{3+}$ concentration, as a result of lattice expansion induced by the substitution of $In^{3+}$ ($r$ = 0.81 Å, CN = 6) by larger $Yb^{3+}$ ($r$ = 0.86 Å, CN = 6). The impurity phase of RbCl (denoted by the star symbol) was observed when the doping concentration of $Yb^{3+}$ was higher than 60 mol%.

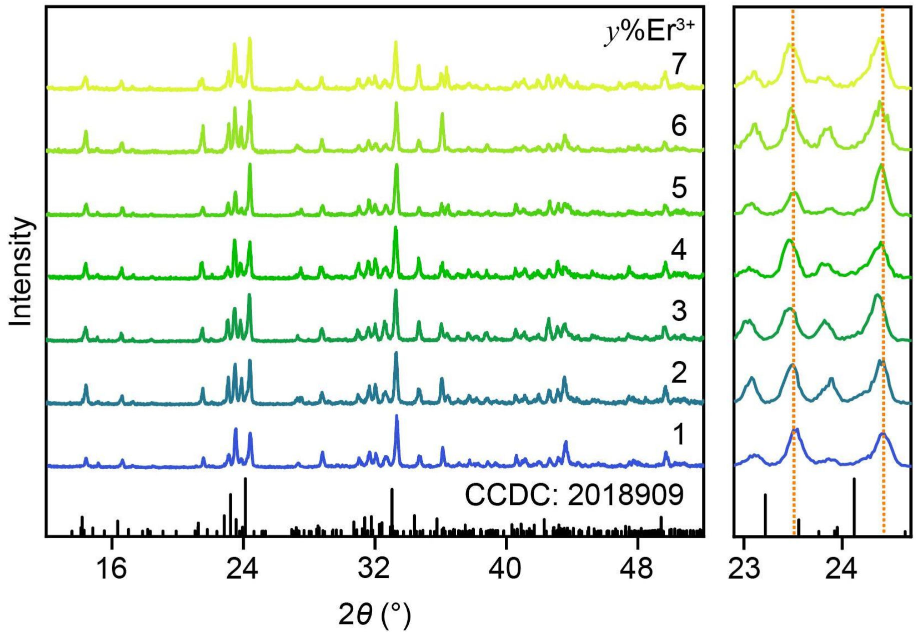


**Supplementary Figure 2.** XRD patterns of $Rb_3InCl_6$: 50%$Yb^{3+}$/*y*%$Er^{3+}$ MCs with different $Er^{3+}$ concentrations. All diffraction peaks of the MCs can be well indexed into monoclinic $Rb_3InCl_6$, indicating high crystallinity and phase purity of the as-synthesized $Rb_3InCl_6$: 50%$Yb^{3+}$/*y*%$Er^{3+}$ MCs. The diffraction peaks of the MCs shifted towards lower angles with the increasing $Er^{3+}$ concentration, as a result of lattice expansion induced by the substitution of $In^{3+}$ ($r$ = 0.81 Å, CN = 6) by larger $Er^{3+}$ ($r$ = 0.88 Å, CN = 6).

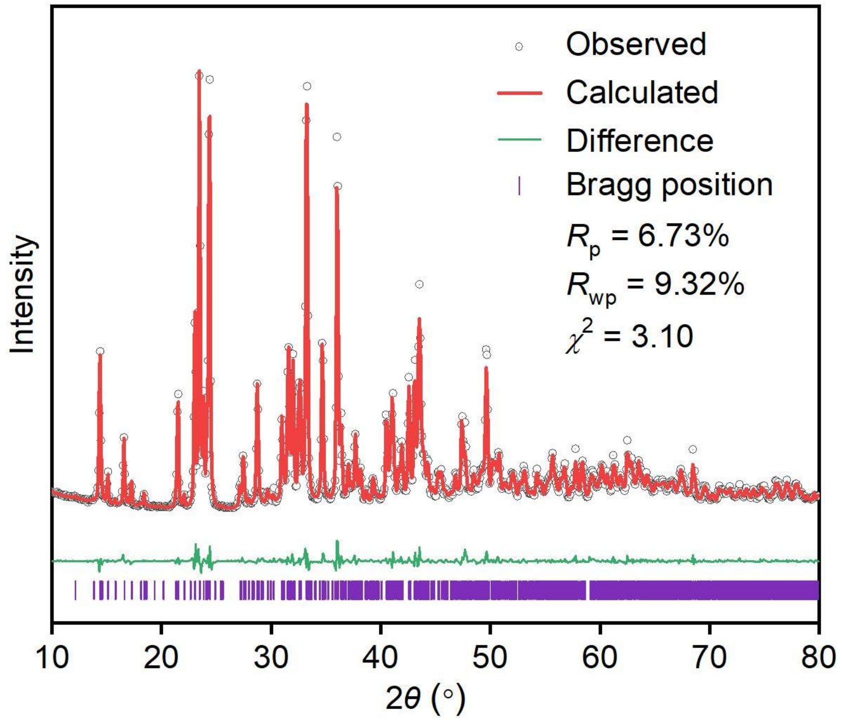


**Supplementary Figure 3.** XRD pattern and Rietveld refinement of $Rb_3InCl_6$ MCs. The white circle, red solid curve, olive solid curve, and violet vertical bar represent the observed, calculated, difference, and Bragg position data, respectively. The goodness-of-fit parameter $\chi^2$ is smaller than 4, and the full spectrum factor ($R_p$) and weighted full spectrum factor ($R_{wp}$) are smaller than 7% and 10%, respectively, demonstrating the reliability of the refinement.

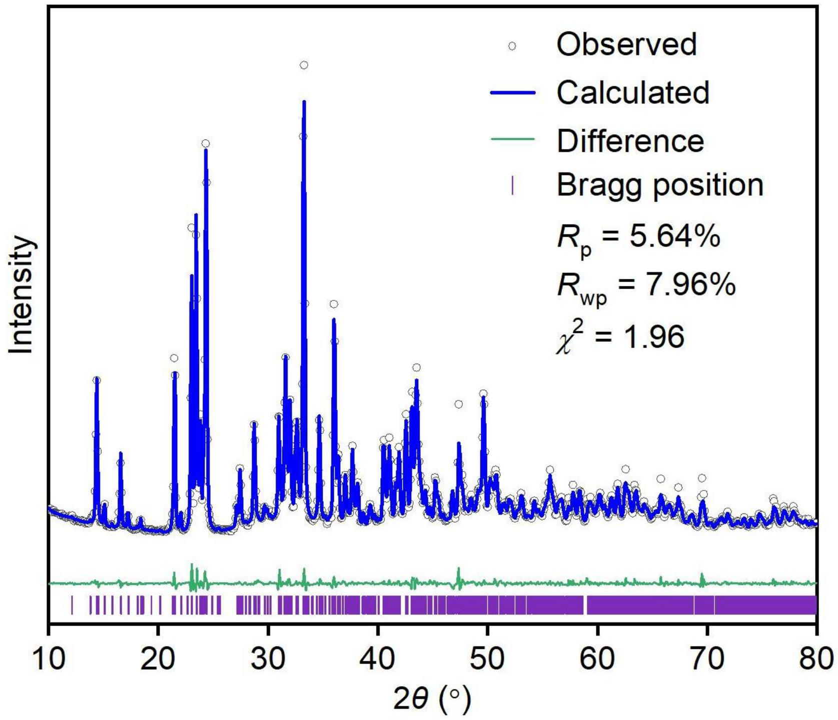


**Supplementary Figure 4.** XRD pattern and Rietveld refinement of $Rb_3InCl_6$: 50%$Yb^{3+}$/1%$Er^{3+}$ MCs. The white circle, blue solid curve, olive solid curve, and violet vertical bar represent the observed, calculated, difference, and Bragg position data, respectively. The goodness-of-fit parameter $\chi^2$ is smaller than 2, and the full spectrum factor ($R_p$) and weighted full spectrum factor ($R_{wp}$) are smaller than 6% and 8%, respectively, demonstrating the reliability of the refinement.

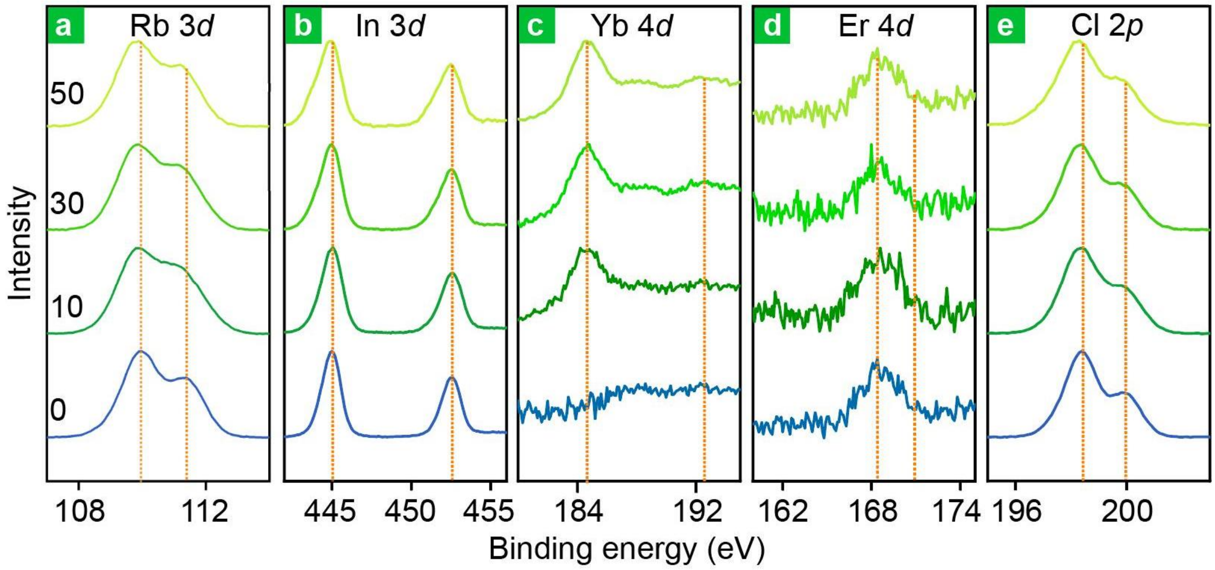


**Supplementary Figure 5.** XPS spectra of $Rb_3InCl_6$: $x$%$Yb^{3+}$/1%$Er^{3+}$ MCs with different $Yb^{3+}$ concentrations. The spectra are shown over the energy regions typical for **a** Rb 3*d*, **b** In 3*d*, **c** Yb 4*d*, **d** Er 4*d*, and **e** Cl 2*p* peaks. The bimodal peaks at the binding energies of 110.0 and 111.4 eV, 445.0 and 452.6 eV, 184.5 and 192.6 eV, 168.6 eV and 170.0 eV, and 198.4 and 200.0 eV can be assigned to the $Rb^+$ $3d_{5/2}$ and $3d_{3/2}$, $In^{3+}$ $3d_{5/2}$ and $3d_{3/2}$, $Yb^{3+}$ $4d_{5/2}$ and $4d_{3/2}$, $Er^{3+}$ $4d_{5/2}$ and $4d_{3/2}$, and $Cl^-$ $2p_{3/2}$ and $2p_{1/2}$, respectively. The $Rb^+$ 3*d*, $Cl^-$ 2*p*, and $In^{3+}$ 3*d* peaks of the MCs shifted towards lower energies with the increasing $Yb^{3+}$ concentration, ascribing to the strengthening of electron densities around $Rb^+$, $In^{3+}$, and $Cl^-$. This implies that $Yb^{3+}$ ions replace the octahedral $In^{3+}$ site in the $Rb_3InCl_6$ lattice.

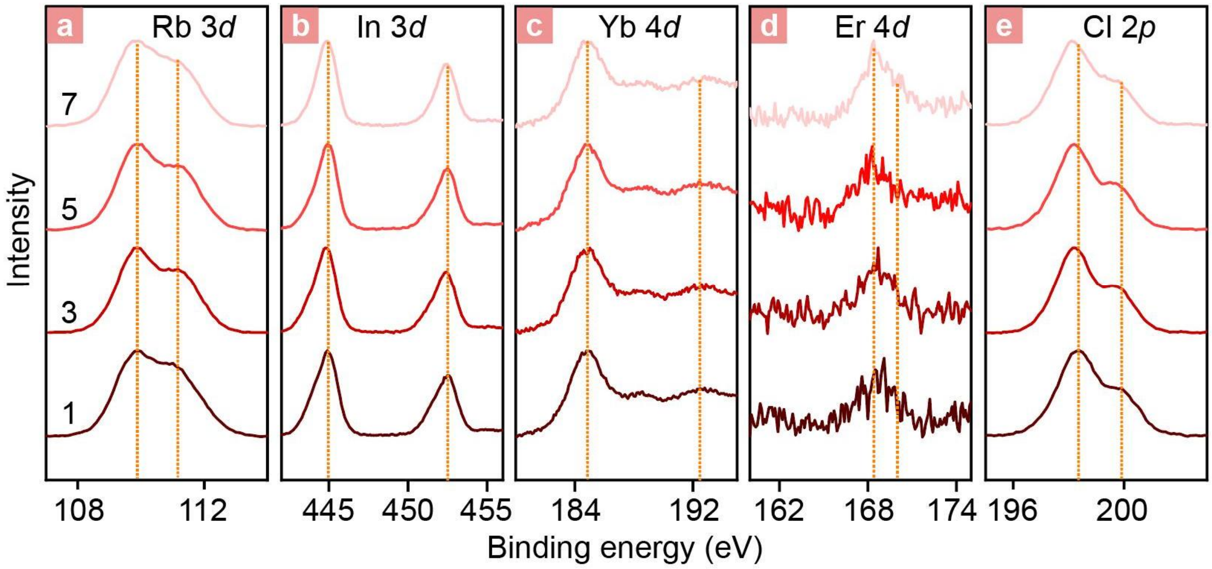


**Supplementary Figure 6.** XPS spectra of $Rb_3InCl_6$: 50%$Yb^{3+}$/*y*%$Er^{3+}$ MCs with different $Er^{3+}$ concentrations. The spectra are shown over the energy regions typical for **a** Rb 3*d*, **b** In 3*d*, **c** Yb 4*d*, **d** Er 4*d*, and **e** Cl 2*p* peaks. The bimodal peaks at the binding energies of 109.8 and 111.2 eV, 444.9 and 452.5 eV, 184.8 and 192.5 eV, 169.1 eV and 170.4 eV, and 198.2 and 199.7 eV can be assigned to the $Rb^+$ $3d_{5/2}$ and $3d_{3/2}$, $In^{3+}$ $3d_{5/2}$ and $3d_{3/2}$, $Yb^{3+}$ $4d_{5/2}$ and $4d_{3/2}$, $Er^{3+}$ $4d_{5/2}$ and $4d_{3/2}$, and $Cl^-$ $2p_{3/2}$ and $2p_{1/2}$, respectively. The $Rb^+$ 3*d*, $Cl^-$ 2*p*, and $In^{3+}$ 3*d* peaks of the MCs shifted towards lower energies with the increasing $Er^{3+}$ concentration, ascribing to the strengthening of electron densities around $Rb^+$, $In^{3+}$, and $Cl^-$. This implies that $Er^{3+}$ ions replace the octahedral $In^{3+}$ site in the $Rb_3InCl_6$ lattice.

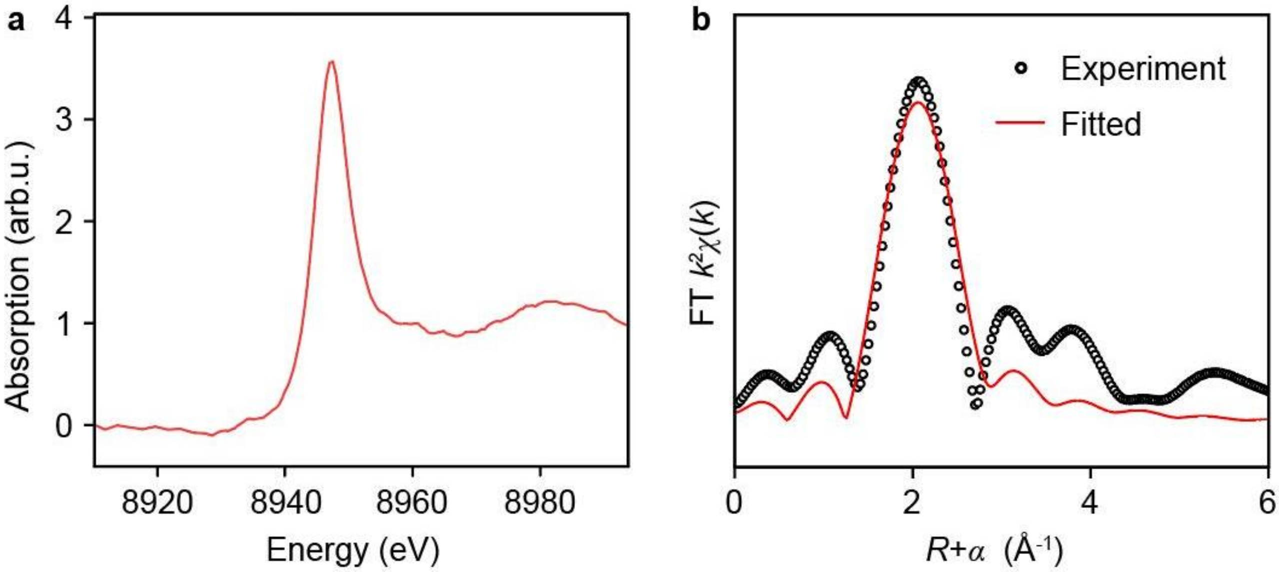


**Supplementary Figure 7. a** XANES spectrum of $Rb_3InCl_6$: 50%$Yb^{3+}$/1%$Er^{3+}$ MCs near the Yb $L_3$-edge. **b** FT-EXAFS of the Yb $L_3$-edge in $Rb_3InCl_6$: 50%$Yb^{3+}$/1%$Er^{3+}$ MCs.

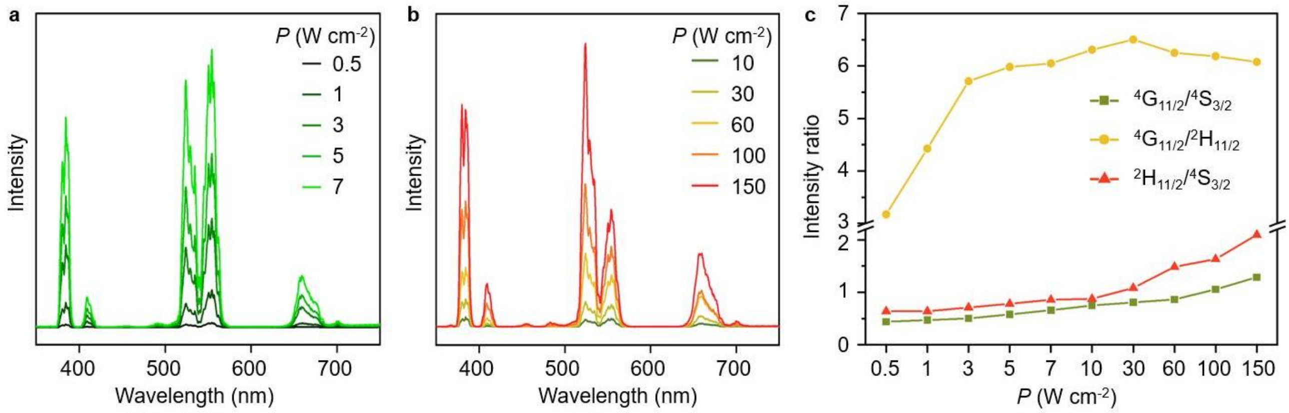


**Supplementary Figure 8.** UCL spectra of $Rb_3InCl_6$: 50%$Yb^{3+}$/1%$Er^{3+}$ MCs upon NIR excitation at 980 nm with power densities in the range of **a** 0.5−7 and **b** 10−150 W $cm^{-2}$, showing unusually strong upconverted UV emission of $Er^{3+}$ at 384 nm. **c** Intensity ratios between the UV and green emissions from $^4G_{11/2}$ and $^4S_{3/2}$ of $Er^{3+}$ ($^4G_{11/2}/^4S_{3/2}$) and between the emissions from the thermally coupled energy levels of $^4G_{11/2}/^2H_{9/2}$ and $^2H_{11/2}/^4S_{3/2}$ of $Er^{3+}$ in $Rb_3InCl_6$: 50%$Yb^{3+}$/1%$Er^{3+}$ MCs as a function of excitation power density. The intensity ratios of $^4G_{11/2}/^4S_{3/2}$ ($I_{384/554}$), $^4G_{11/2}/^2H_{9/2}$ ($I_{384/409}$), and $^2H_{11/2}/^4S_{3/2}$ ($I_{524/554}$) of $Er^{3+}$ increased from 0.441, 3.173, and 0.639 to 1.286, 6.077, and 2.095, respectively, with the increasing power density from 0.5 to 150 W $cm^{-2}$. This indicates that the intensity ratios of $I_{384}/I_{554}$, $I_{384/409}$, and $I_{524/554}$ depend strongly on the excitation power density, which is not unexpected since the nonlinear UCL is power dependent. Specifically, the intensity of UV emission of $Er^{3+}$ is comparable to that of the green emission ($I_{384}/I_{554}$ = 0.471) even under excitation with a low power density of 1 W $cm^{-2}$, wherein the laser-induced heating effect is negligible.

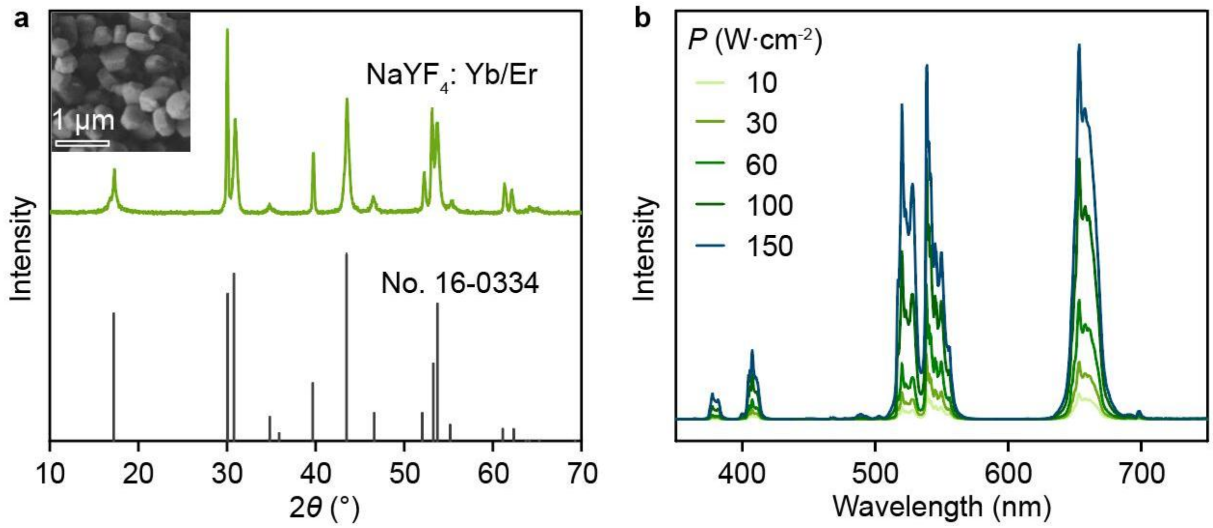


**Supplementary Figure 9. a** XRD pattern, scanning electron microscopy (SEM) image, and **b** UCL spectra of $NaYF_4$: 18%$Yb^{3+}$/2%$Er^{3+}$ MCs upon NIR excitation at 980 nm with power densities ranging from 10 to 150 W $cm^{-2}$. The inset in **a** presents the SEM image of the MCs, showing that the MCs had a hexagonal platelet morphology with an average length of ≈580 nm and a thickness of ≈260 nm.

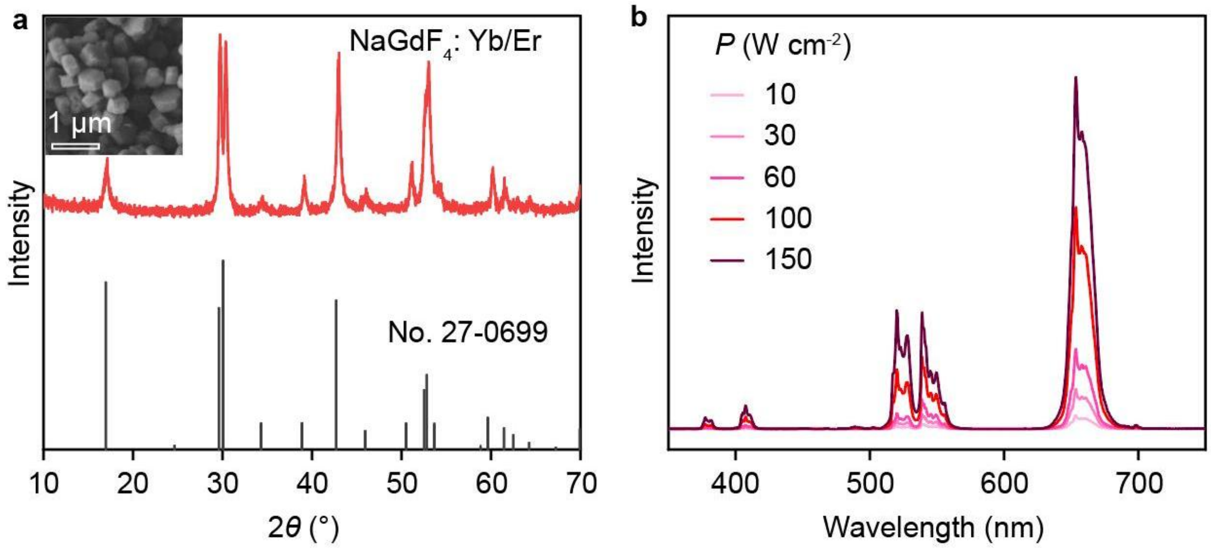


**Supplementary Figure 10. a** XRD pattern, SEM image, and **b** UCL spectra of $NaGdF_4$: 18%$Yb^{3+}$/2%$Er^{3+}$ MCs upon NIR excitation at 980 nm with power densities ranging from 10 to 150 W $cm^{-2}$. The inset in **a** presents the SEM image of the MCs, showing that the MCs had a hexagonal platelet morphology with an average length of ≈600 nm and a thickness of ≈270 nm.

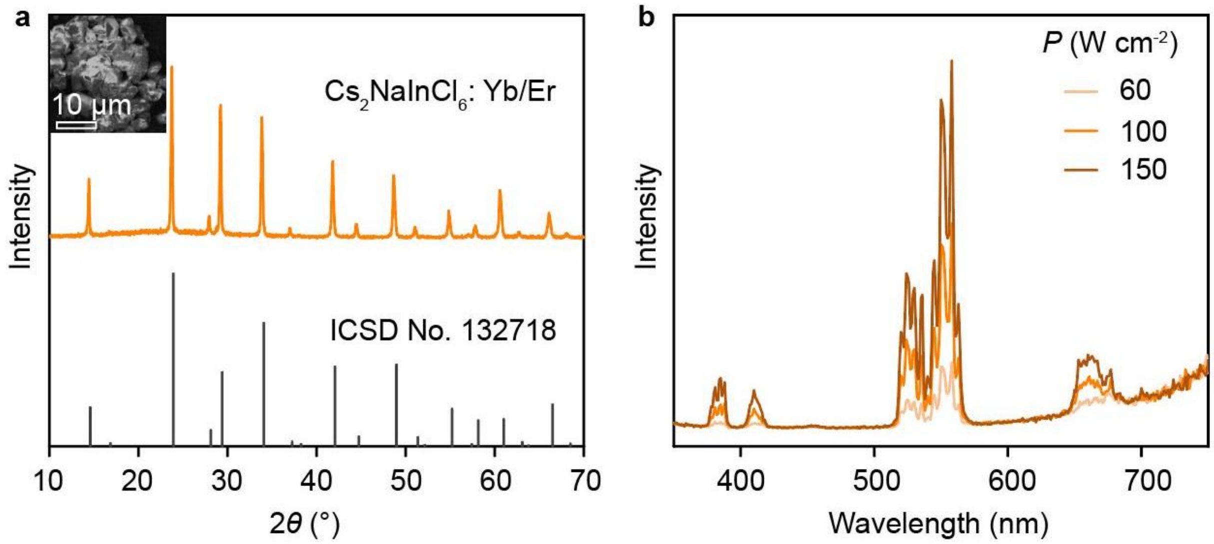


**Supplementary Figure 11.** **a** XRD pattern and **b** UCL spectra of $CsNaInCl_6$: 50%$Yb^{3+}$/1%$Er^{3+}$ MCs upon NIR excitation at 980 nm with power densities ranging from 60 to 150 W $cm^{-2}$. The inset in **a** presents the SEM image of the MCs, showing that the MCs had an irregular morphology with a broad size distribution in the range of 2–20 μm. Note that the UC efficiency of $Yb^{3+}$/$Er^{3+}$ in $CsNaInCl_6$ is much lower than that in $Rb_3InCl_6$, $NaYF_4$, and $NaGdF_4$. Therefore, the UCL spectra of $CsNaInCl_6$: 50%$Yb^{3+}$/1%$Er^{3+}$ MCs should be measured at a relatively higher power density and exhibited a low signal-to-noise ratio.

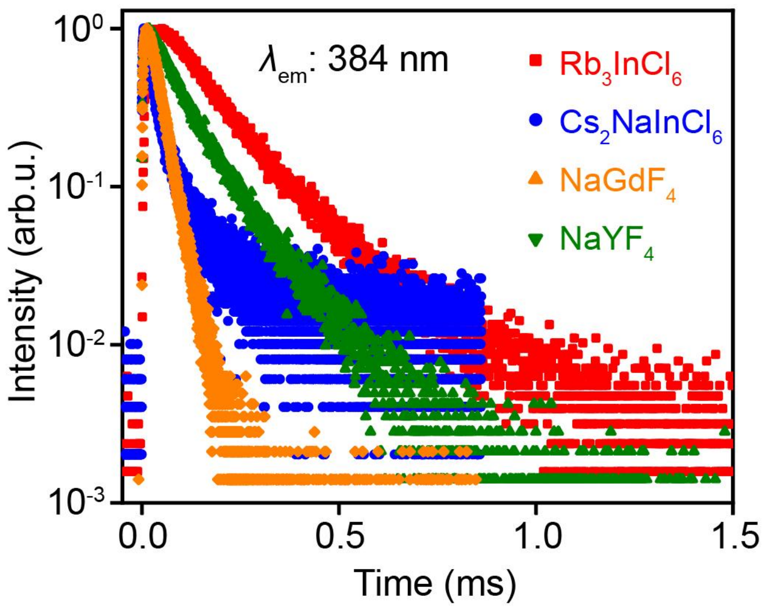


**Supplementary Figure 12.** UCL decay curves from $^4G_{11/2}$ ($\lambda_{em}$: ≈384 nm) of $Er^{3+}$ in $Yb^{3+}/Er^{3+}$ co-doped $Rb_3InCl_6$ (50 mol%/1 mol%), $Cs_2NaInCl_6$ (50 mol%/1 mol%), $NaGdF_4$ (18 mol%/2 mol%), and $NaYF_4$ (18 mol%/2 mol%) MCs upon 980-nm excitation, showing significantly longer UCL lifetime of $^4G_{11/2}$ of $Er^{3+}$ in $Rb_3InCl_6$ than in the other upconversion phosphors.

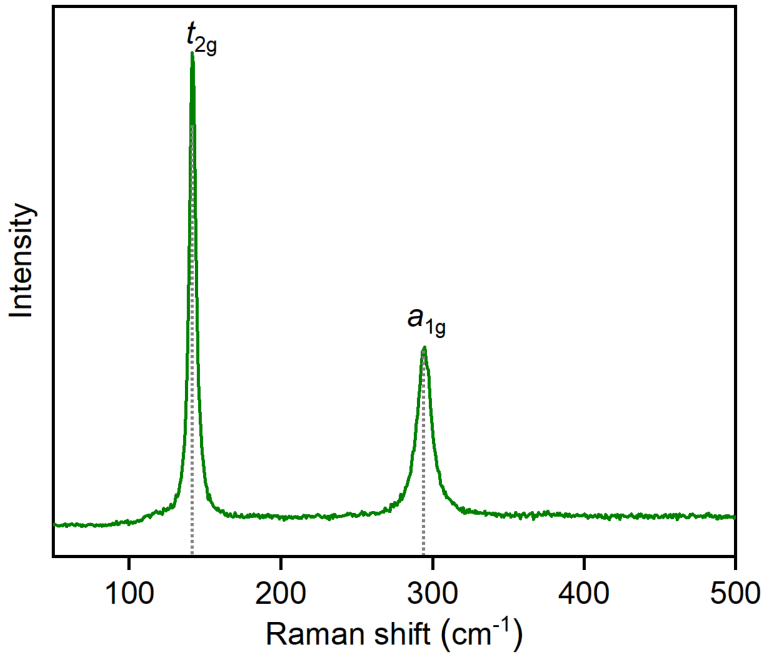


**Supplementary Figure 13.** Raman spectra of $Cs_2NaInCl_6$: 50%$Yb^{3+}$/1%$Er^{3+}$ MCs, showing the cutoff phonon energy of around 295 $cm^{-1}$. $Cs_2NaInCl_6$: 50%$Yb^{3+}$/1%$Er^{3+}$ MCs exhibit two intense vibrational peaks, corresponding to the breathing vibrations of Cl atoms ($t_{2g}$: 141 $cm^{-1}$) and the symmetric stretching vibrations of In–Cl bonds ($a_{1g}$: 295 $cm^{-1}$) in the $[InCl_6]^{5-}$ octahedra, respectively.

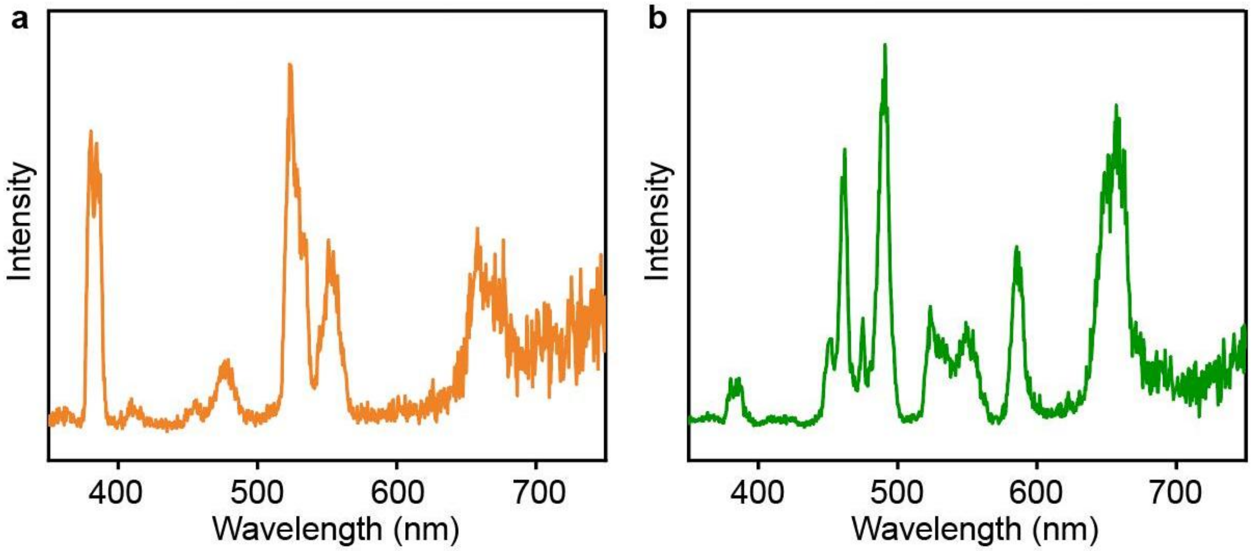


**Supplementary Figure 14.** UCL spectra of **a** $Rb_3InCl_6$: 50% $Yb^{3+}$/1%$Tm^{3+}$ and **b** $Rb_3InCl_6$: 50% $Yb^{3+}$/1%$Ho^{3+}$ MCs upon NIR excitation at 980 nm with a power density of 100 W cm$^{-2}$. Owing to the low phonon energies of $Rb_3InCl_6$ that can not bridge the energy gap between $^3H_5$ of $Tm^{3+}$ (or $^5I_6$ of $Ho^{3+}$) and $^2F_{5/2}$ of $Yb^{3+}$, $Rb_3InCl_6$: $Yb^{3+}$/$Tm^{3+}$ and $Rb_3InCl_6$: $Yb^{3+}$/$Ho^{3+}$ MCs exhibited negligibly weak UCL. Note that the emissions from $Er^{3+}$ impurities were observed in the UCL spectra.

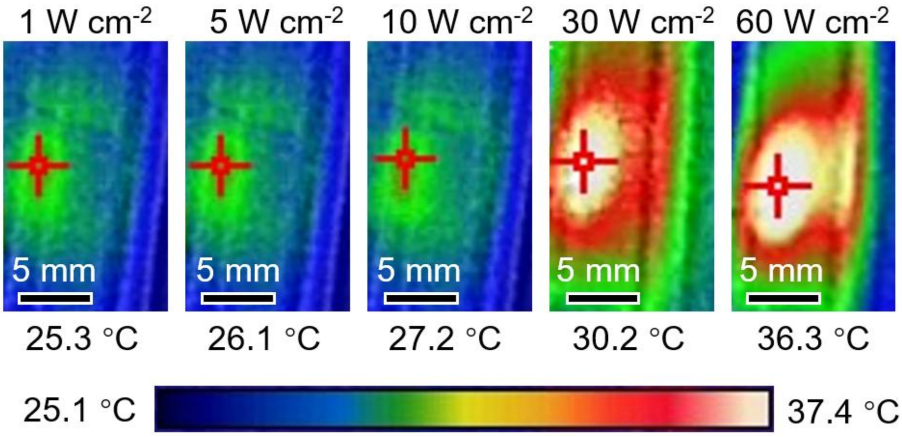


**Supplementary Figure 15.** Thermographs for the powder sample of $Rb_3InCl_6$: 50%$Yb^{3+}$/1%$Er^{3+}$ MCs upon 980-nm excitation at power densities of 1, 5, 10, 30, and 60 W $cm^{-2}$ for 2 min, recorded by an infrared thermographic camera. Owing to the laser heating effect, the temperature of the sample increased from room temperature (25.0 °C) to 25.3, 26.1, 27.2, 30.2, and 36.3 °C, respectively. Note that the UCL measurement was accompanied in 2 min.

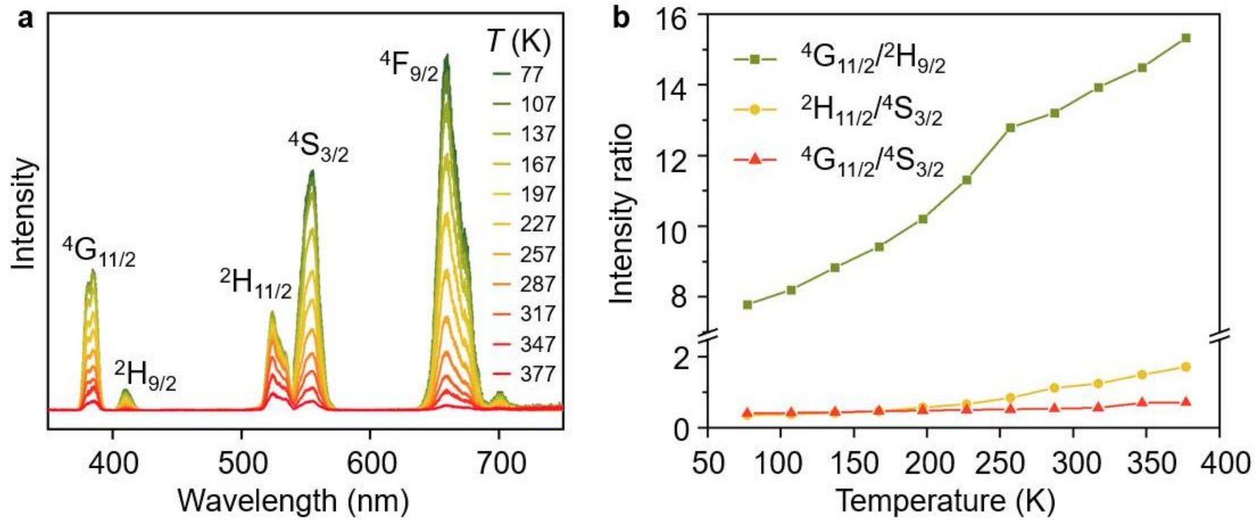


**Supplementary Figure 16. a** Temperature-dependent UCL spectra of $Rb_3InCl_6$: 50%$Yb^{3+}$/1%$Er^{3+}$ MCs upon 980-nm excitation at a power density of 10 W $cm^{-2}$. **b** Intensity ratios between the UV and green emissions from $^4G_{11/2}$ and $^4S_{3/2}$ of $Er^{3+}$ ($^4G_{11/2}/^4S_{3/2}$) and between the emissions from the thermally coupled energy levels of $^4G_{11/2}/^2H_{9/2}$ and $^2H_{11/2}/^4S_{3/2}$ of $Er^{3+}$ in $Rb_3InCl_6$: 50%$Yb^{3+}$/1%$Er^{3+}$ MCs as a function of temperature. The overall UCL intensity of the MCs decreased gradually with the temperature rise from 77 K to 377 K, as a result of accelerated nonradiative relaxation of $Yb^{3+}$ and $Er^{3+}$ at higher temperatures. Concurrently, the intensity ratios of $^4G_{11/2}/^4S_{3/2}$ ($I_{384/554}$), $^4G_{11/2}/^2H_{9/2}$ ($I_{384/409}$), and $^2H_{11/2}/^4S_{3/2}$ ($I_{524/554}$) of $Er^{3+}$ increased from 0.408, 7.791 and 0.357 at 77 K to 0.718, 15.34 and 1.726 at 377 K, respectively, due to the thermal-enhanced population of $Er^{3+}$ at higher states ($^4G_{11/2}$ and $^2H_{11/2}$). Notably, the intensity ratio of $^4G_{11/2}/^4S_{3/2}$ ($I_{384/554}$) remained abnormally large (0.408) at 77 K, as compared to that of conventional UC materials.

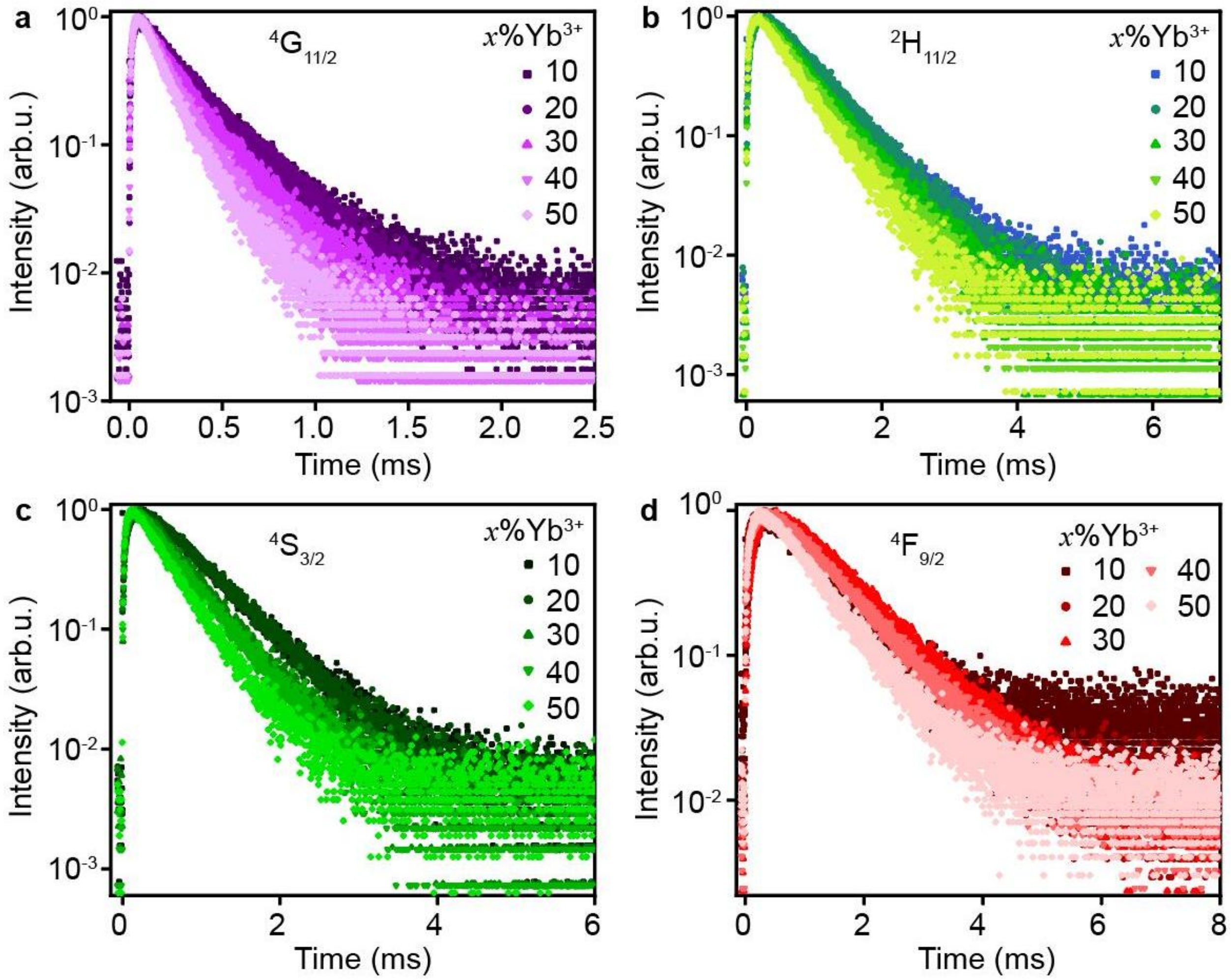


**Supplementary Figure 17**. UCL decay curves from **a** $^4G_{11/2}$ (384 nm), **b** $^2H_{11/2}$ (524 nm), **c** $^4S_{3/2}$ (554 nm), and **d** $^4F_{9/2}$ (659 nm) of $Er^{3+}$ in $Rb_3InCl_6$: $x$%$Yb^{3+}$/1%$Er^{3+}$ MCs with different $Yb^{3+}$ concentrations. The effective UCL lifetimes of $^4G_{11/2}$, $^2H_{11/2}$, $^4S_{3/2}$, and $^4F_{9/2}$ of $Er^{3+}$ were determined to decrease from 311, 1000, 975, and 1580 μs to 190, 689, 609, and 1195 μs, respectively, as the $Yb^{3+}$ concentration increased from 10 to 50 mol% (see also Supplementary Table 8).

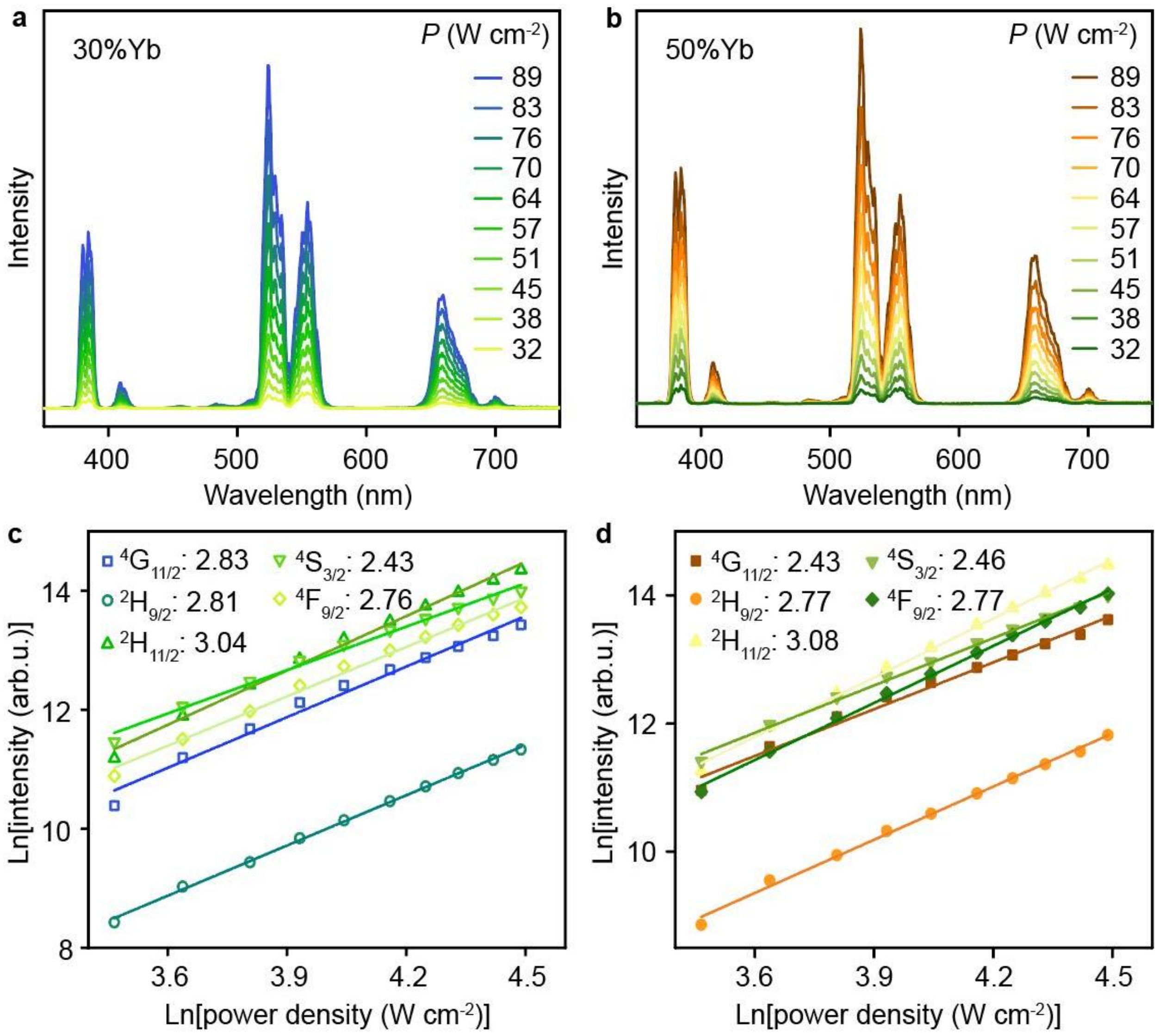


**Supplementary Figure 18**. Power-dependent UCL spectra of **a** $Rb_3InCl_6$: 30%$Yb^{3+}$/1%$Er^{3+}$ and **b** $Rb_3InCl_6$: 50%$Yb^{3+}$/1%$Er^{3+}$ MCs under 980 nm excitation. **c, d** show the corresponding power dependence of the UCL for the $^4G_{11/2} \rightarrow {}^4I_{15/2}$ (384 nm), $^2H_{9/2} \rightarrow {}^4I_{15/2}$ (409 nm), $^2H_{11/2} \rightarrow {}^4I_{15/2}$ (524 nm), $^4S_{3/2} \rightarrow {}^4I_{15/2}$ (554 nm), and $^4F_{9/2} \rightarrow {}^4I_{15/2}$ (659 nm) transitions of $Er^{3+}$.

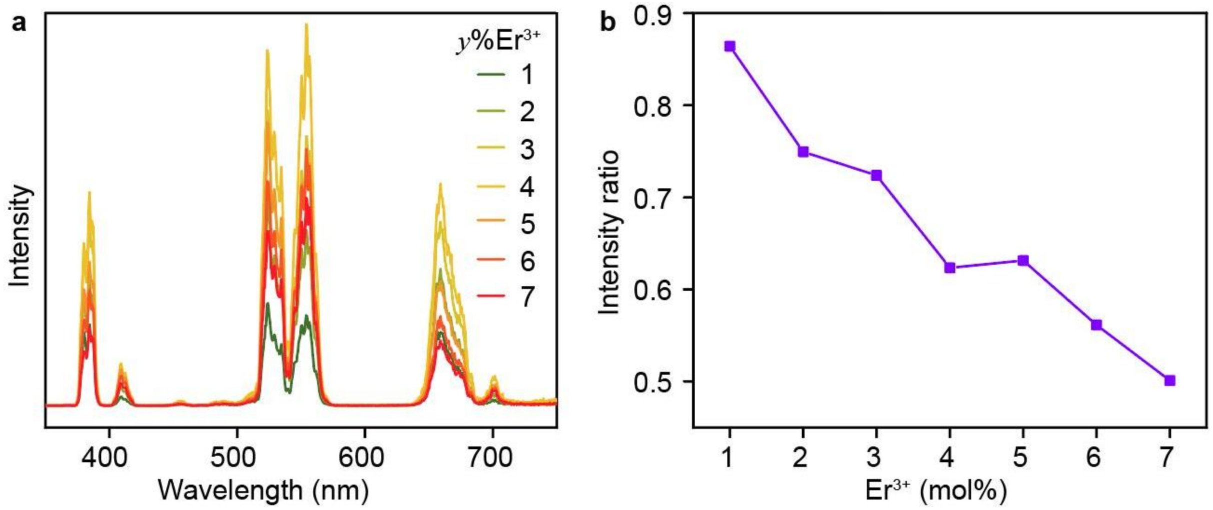


**Supplementary Figure 19**. **a** UCL spectra of $Rb_3InCl_6$: 50%$Yb^{3+}$/*y*%$Er^{3+}$ MCs with different $Er^{3+}$ concentrations under NIR excitation at 980 nm with a power density of 60 W $cm^{-2}$. **b** Intensity ratio between the UV and green emissions ($I_{384}/I_{554}$) of $Er^{3+}$ as a function of the $Er^{3+}$ concentration.

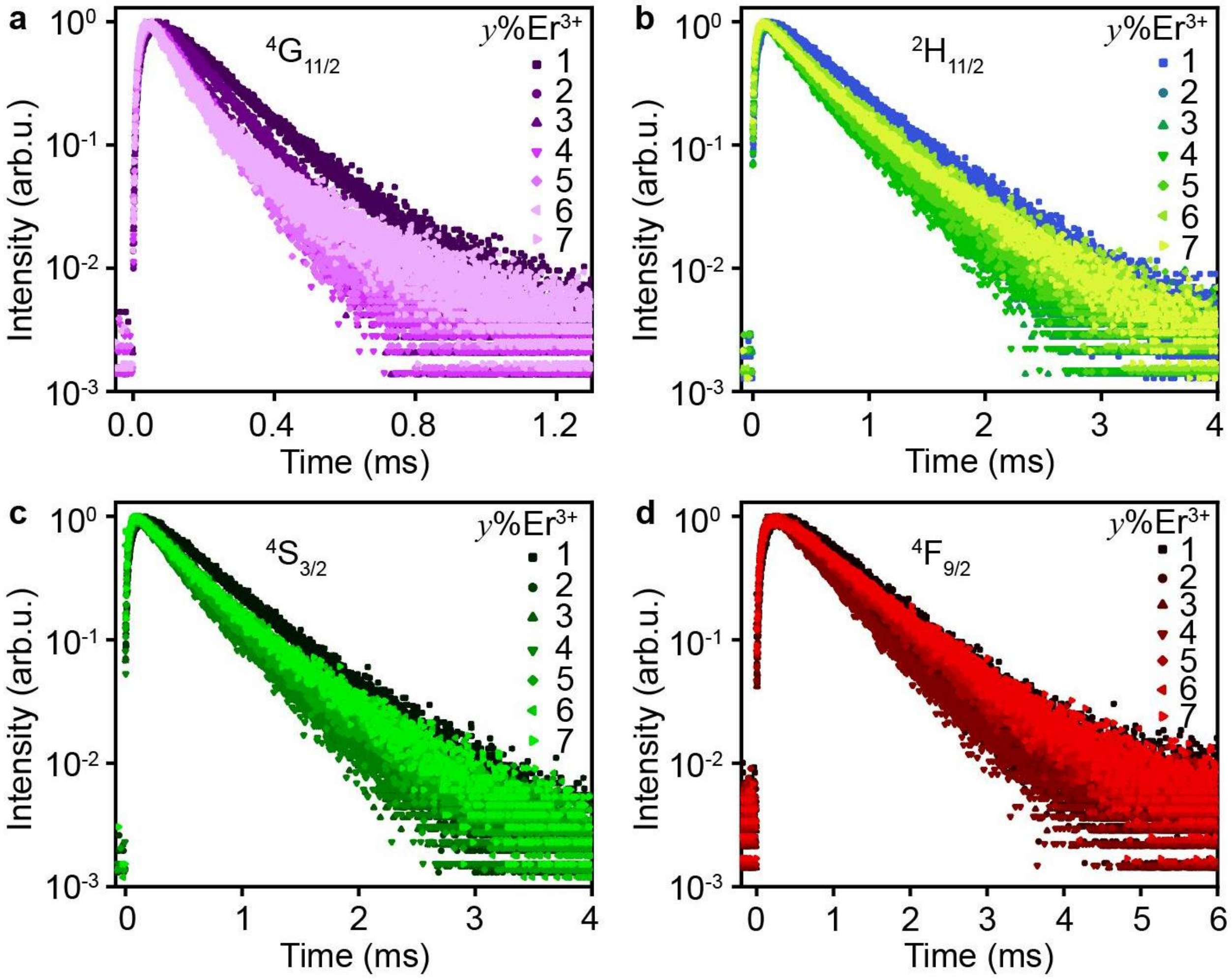


**Supplementary Figure 20**. UCL decay curves from **a** $^4G_{11/2}$ (384 nm), **b** $^2H_{11/2}$ (524 nm), **c** $^4S_{3/2}$ (554 nm), and **d** $^4F_{9/2}$ (659 nm) of $Er^{3+}$ in $Rb_3InCl_6$: 50%$Yb^{3+}$/*y*%$Er^{3+}$ MCs with different $Er^{3+}$ concentrations. The effective UCL lifetimes of $^4G_{11/2}$, $^2H_{11/2}$, $^4S_{3/2}$, and $^4F_{9/2}$ of $Er^{3+}$ were determined to decrease from 190, 689, 609, and 1195 μs to 141, 588, 579, and 1174 μs, respectively, as the $Er^{3+}$ concentration increased from 1 to 7 mol% (see also Supplementary Table 9).

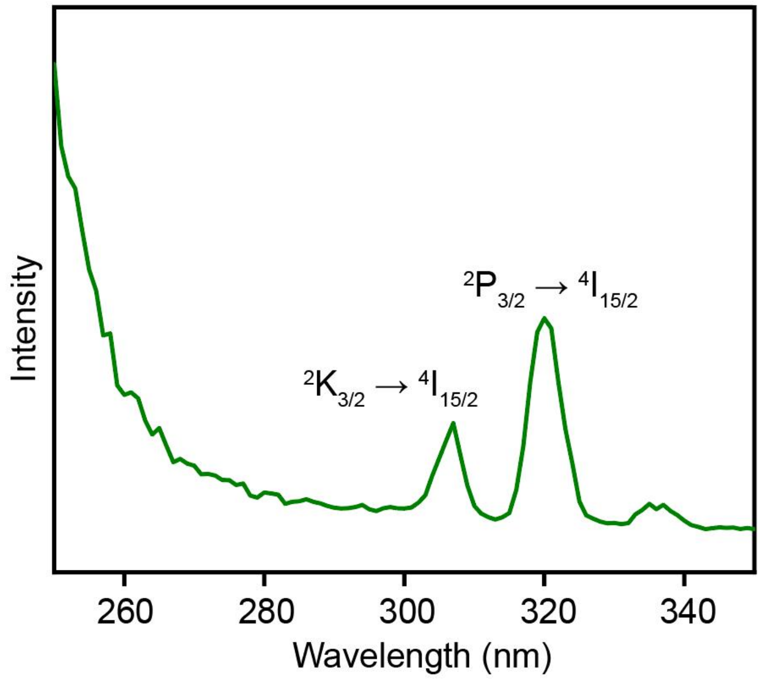


**Supplementary Figure 21**. UCL spectrum of $Rb_3InCl_6$: 50%$Yb^{3+}$/1%$Er^{3+}$ MCs under 980-nm excitation at a power density of 200 W $cm^{-2}$. Owing to the low phonon energies that enable the high-order UCL processes, the four-photon UCL from $^2K_{3/2}$ (307 nm) and $^2P_{3/2}$ (320 nm) of $Er^{3+}$ can be explicitly observed in $Rb_3InCl_6$: $Yb^{3+}$/$Er^{3+}$ MCs.

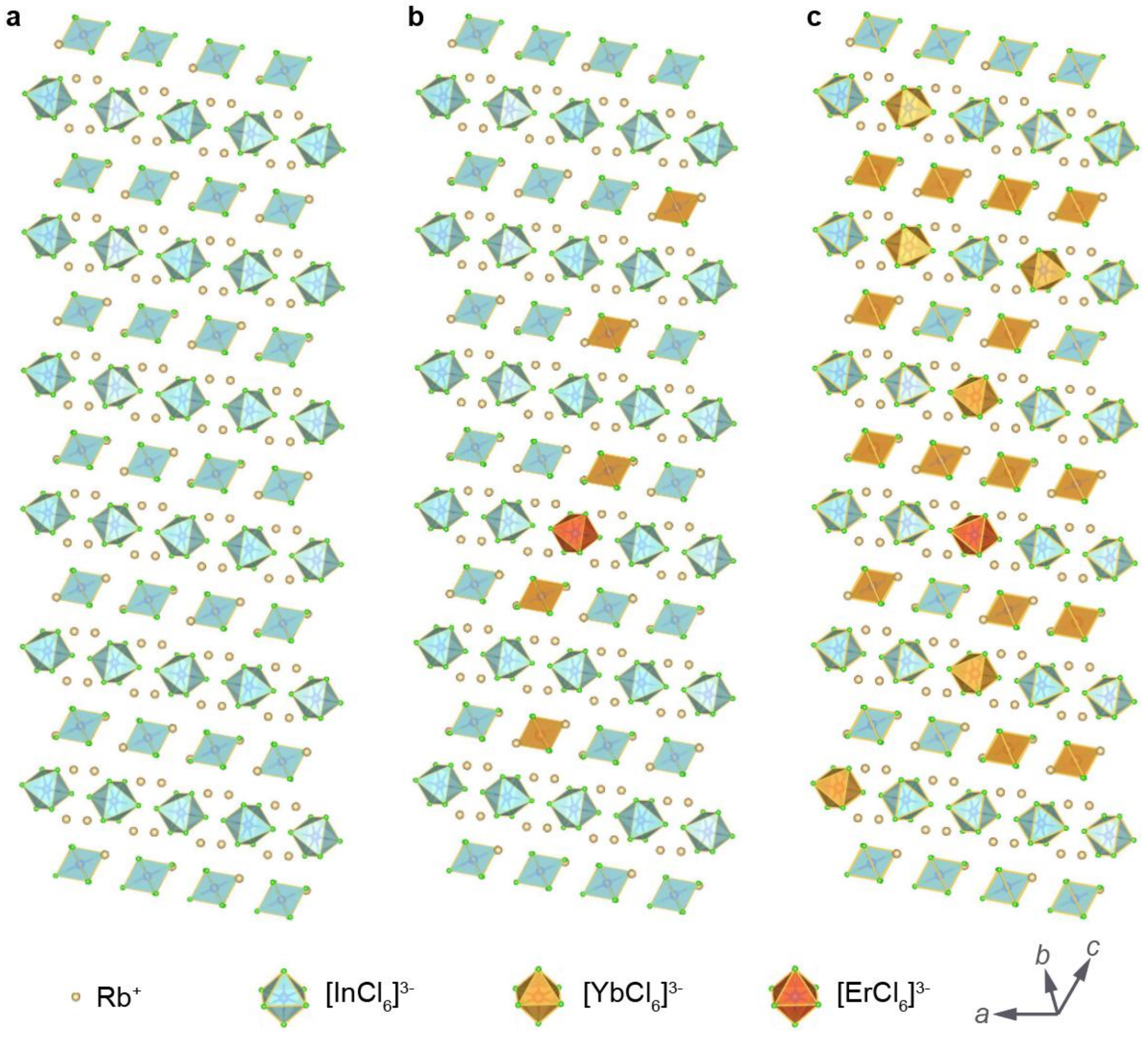


**Supplementary Figure 22**. Optimized crystal structures of **a** $Rb_3InCl_6$, **b** $Rb_3InCl_6$: 10%$Yb^{3+}$/1%$Er^{3+}$, and **c** $Rb_3InCl_6$: 50%$Yb^{3+}$/1%$Er^{3+}$ by using 1 × 1 × 1 Monkhorst-Pack *k*-point sampling for 3 × 3 × 2 supercell structure. The Yb−Yb and Yb−Er distances were calculated to be 7.67 Å and 7.40 Å in $Rb_3InCl_6$: 10%$Yb^{3+}$/1%$Er^{3+}$ and 7.50 Å and 7.14 Å in $Rb_3InCl_6$: 50%$Yb^{3+}$/1%$Er^{3+}$, respectively.

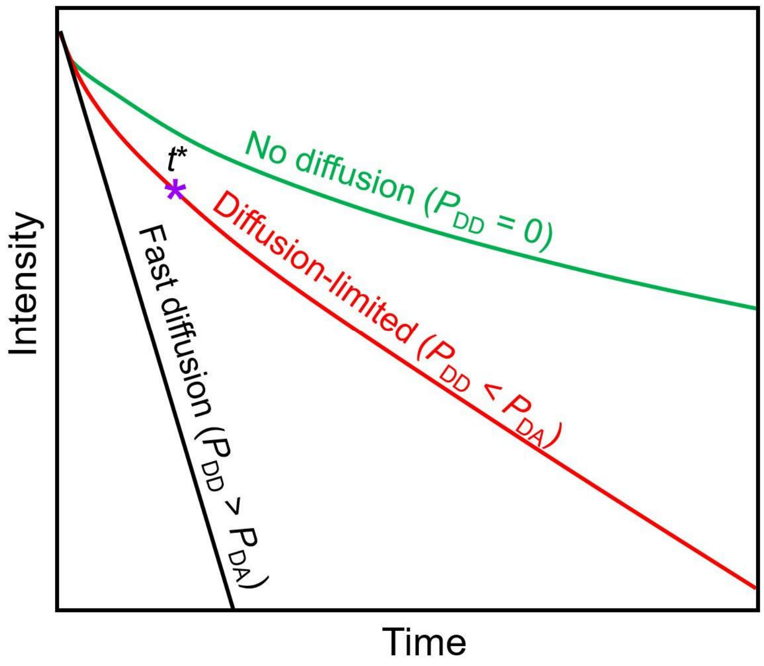


**Supplementary Figure 23**. Representative PL decay curves of the donor within the framework of energy diffusion modes: direct ET without diffusion ($P_{\mathrm{DD}} = 0$), diffusion-limited ($P_{\mathrm{DD}} < P_{\mathrm{DA}}$), and fast-diffusion ($P_{\mathrm{DD}} > P_{\mathrm{DA}}$). The boundary between the non-exponential and exponential decay portions in the diffusion-limited mode can be marked by a characteristic time $t^*$. According to M. Yokota and O. Tanimoto,[1] the donor decay function for diffusion-limited migration in a three-dimensional sublattice can be expressed as:

$$I(t) = I_0 \exp\left[-\frac{t}{\tau_0} - \frac{4}{3}\pi^{\frac{3}{2}} C_{\mathrm{A}} (\mathrm{C}t)^{\frac{1}{2}} \left(\frac{1 + 10.87x + 15.50x^2}{1 + 8.743x}\right)^{3/4}\right] \quad (1)$$

where $x = \mathrm{DC}^{-1/3}t^{2/3}$, D is the diffusion constant, C is the interaction parameter for donor-acceptor, and $C_{\mathrm{A}}$ is the concentration of acceptors. At early times with $t \ll t^*$ ($t^* = \mathrm{C}^{1/2}/\mathrm{D}^{2/3}$), the time is not sufficient for the excitation energy to diffuse among the donors before being transferred to nearby acceptors, therefore, energy diffusion (migration) is negligible. At $t \gg t^*$, Eq. (1) can be reduced to:

$$I(t) = I_0 \exp\left(-\frac{t}{\tau_0} - K_{\mathrm{D}} t\right) = I_0 \exp\left(-\frac{t}{\tau}\right) \quad (2)$$

where

$$K_{\mathrm{D}} = 4\pi \mathrm{D} C_{\mathrm{A}} R_{\mathrm{D}} \quad (3)$$

$$R_{\mathrm{D}} = 0.91 \left(\frac{\mathrm{C}}{\mathrm{D}}\right)^{1/4} \quad (4)$$

$$\frac{1}{\tau} = \frac{1}{\tau_0} + K_{\mathrm{D}} \quad (5)$$

Eq. (2) verifies that the PL decay of diffusion-limited migration becomes exponential in long times after pulse excitation, where the decay rate of the slow exponential portion is determined by the radiation and migration rate. The decay rate due to migration ($K_D = 1/\tau_D$) can thus be obtained from Eq. (5), where $1/\tau$ is the observed decay rate derived from the exponential portion of the decay curve and $1/\tau_0$ is the radiative rate obtained from the low-doping (1 mol% $Yb^{3+}$) sample.

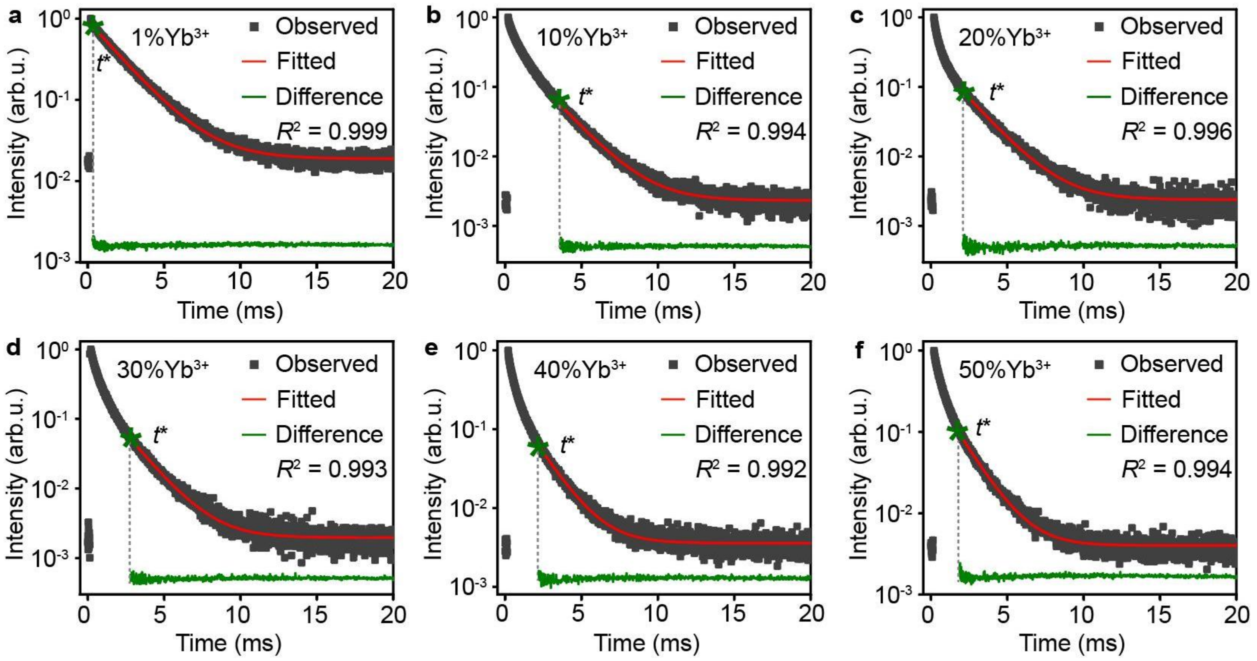


**Supplementary Figure 24**. PL decay curves of $Yb^{3+}$ in $Rb_3InCl_6$: $x$%$Yb^{3+}$ MCs with different $Yb^{3+}$ concentrations by monitoring the $Yb^{3+}$ emission at 994 nm upon pulsed laser excitation at 930 nm. The observed decay times ($\tau$) of $Yb^{3+}$ and the decay rates due to migration ($1/\tau_D$) were derived based on the diffusion-limited mode by single-exponential fitting to the exponential portion of the decay curves. The boundary between the non-exponential and exponential decay portions is marked by the characteristic time $t$*. The goodness-of-fit parameters $R^2$ are higher than 0.99, demonstrating the reliability of the fitting.

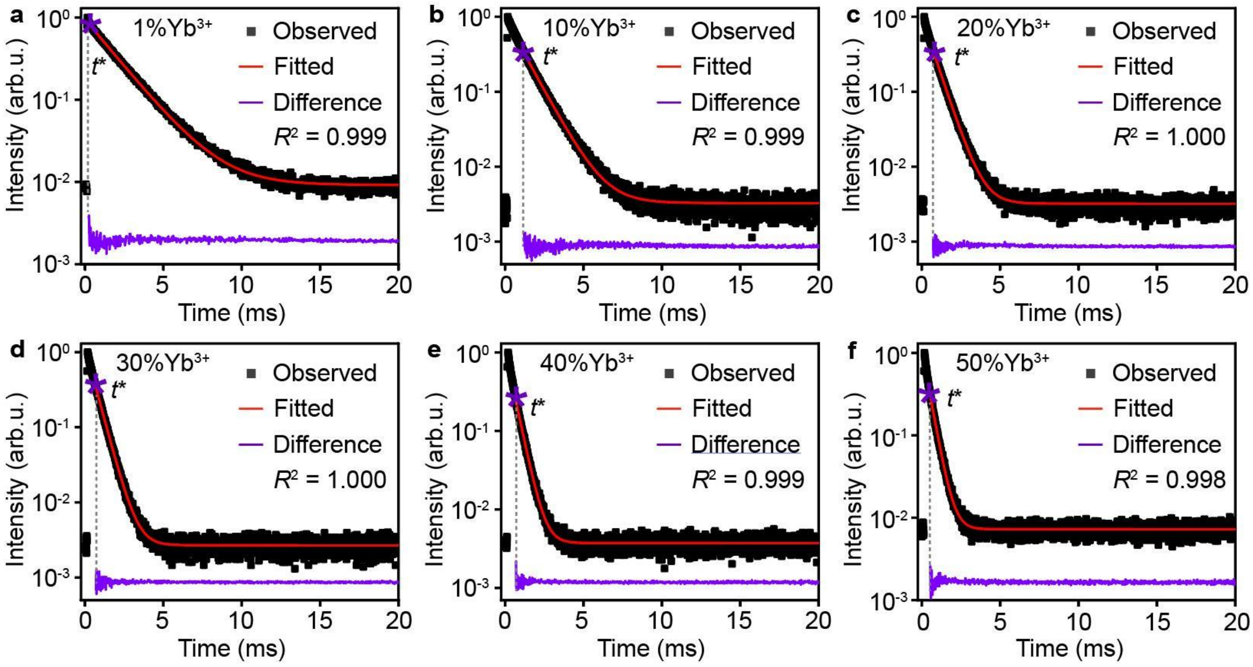


**Supplementary Figure 25**. PL decay curves of $Yb^{3+}$ in $NaYF_4$: $x$%$Yb^{3+}$ MCs with different $Yb^{3+}$ concentrations by monitoring the $Yb^{3+}$ emission at 994 nm upon pulsed laser excitation at 930 nm. The observed decay times ($\tau$) of $Yb^{3+}$ and the decay rates due to migration ($1/\tau_D$) were derived based on the diffusion-limited mode by single-exponential fitting to the exponential portion of the decay curves. The boundary between the non-exponential and exponential decay portions is marked by the characteristic time $t$*. The goodness-of-fit parameters $R^2$ are higher than 0.99, demonstrating the reliability of the fitting.

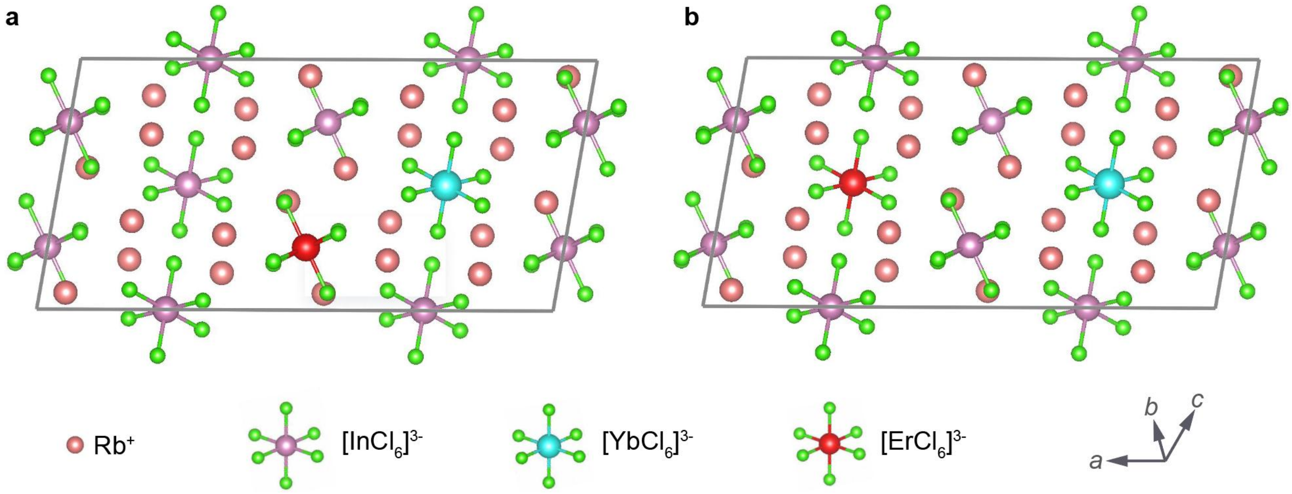


**Supplementary Figure 26**. Crystal structures of $Rb_3InCl_6$: $Yb^{3+}/Er^{3+}$, showing the location of $Yb^{3+}$ and $Er^{3+}$ at **a** close and **b** distant positions, respectively. In order to identify the positions of the doping ions, the system energy of $Yb^{3+}$ and $Er^{3+}$ at different doping positions in a $Rb_3InCl_6$ unit cell was calculated using the Vienna ab initio simulation package (VASP) code employing the GGA with PBE functions. The results showed that the total energy (-278.47 eV) of the system with $Yb^{3+}$ and $Er^{3+}$ located at two close proximity positions is lower than that (-276.56 eV) of the system with $Yb^{3+}$ and $Er^{3+}$ located at two distant positions. Therefore, it is deduced that $Yb^{3+}$ and $Er^{3+}$ are more likely to be distributed in close proximity to each other in the $Rb_3InCl_6$ lattice. Note that the theoretical calculations represent only idealized outcomes, because the physical experiments, especially the chemical synthesis of the NCs, are intricate and multifaceted. Nonetheless, they can provide a general picture of preferential distributions of the $Yb^{3+}$ and $Er^{3+}$ dopants in the $Rb_3InCl_6$ lattice.

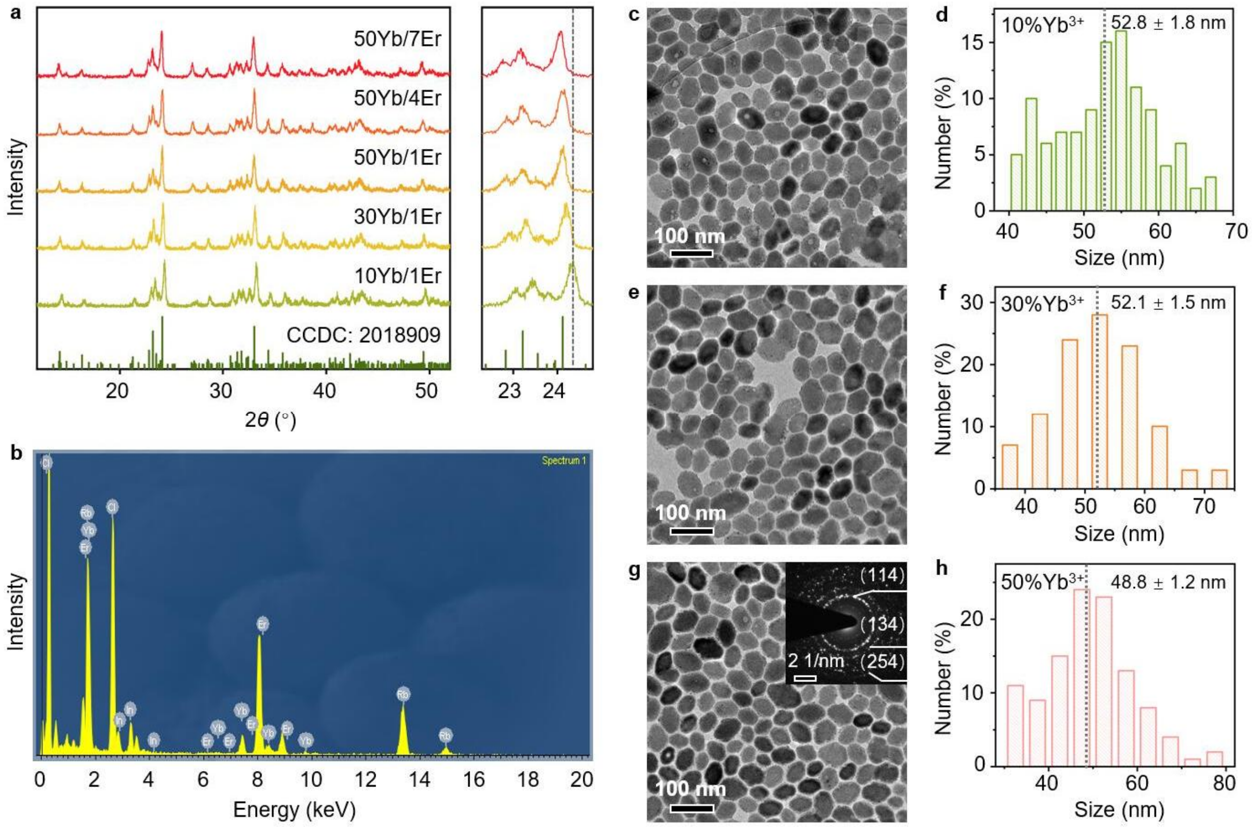


**Supplementary Figure 27**. **a** XRD patterns of $Rb_3InCl_6$: *x*%$Yb^{3+}$/*y*%$Er^{3+}$ NCs with different $Yb^{3+}$ and $Er^{3+}$ concentrations. The bottom lines represent the standard XRD pattern of monoclinic $Rb_3InCl_6$ (CCDC No. 2018909). **b** Energy dispersive X-ray spectra of $Rb_3InCl_6$: 50%$Yb^{3+}$/1%$Er^{3+}$ NCs, showing the elements of Rb, In, Cl, Yb, and Er in the NCs. Transmission electron microscopy (TEM) images and size distribution histograms of **c, d** $Rb_3InCl_6$: 10%$Yb^{3+}$/1%$Er^{3+}$, **e, f** $Rb_3InCl_6$: 30%$Yb^{3+}$/1%$Er^{3+}$, and **g, h** $Rb_3InCl_6$: 50%$Yb^{3+}$/1%$Er^{3+}$ NCs. The size distributions of the NCs were obtained by randomly calculating 200 particles in the TEM image. The inset in **g** shows the selected area electron diffraction (SAED) pattern of the NCs. All diffraction peaks of the NCs can be well indexed into monoclinic $Rb_3InCl_6$, indicating high crystallinity and phase purity of the as-synthesized $Rb_3InCl_6$: *x*%$Yb^{3+}$/ *y*%$Er^{3+}$ NCs. The diffraction peaks of the NCs shifted towards lower angles with the increasing $Yb^{3+}$ and $Er^{3+}$ concentrations, as a result of lattice expansion induced by the substitution of $In^{3+}$ (r = 0.81 Å, CN = 6) by larger $Yb^{3+}$ (r = 0.86 Å, CN = 6) and $Er^{3+}$ (r = 0.88 Å, CN = 6). TEM images showed that the NCs were roughly monodispersed with mean sizes in the range of 48.8–52.8 nm. The doping concentrations of $Yb^{3+}$ and $Er^{3+}$ had no significant influence on the size and morphology of the resulting NCs.

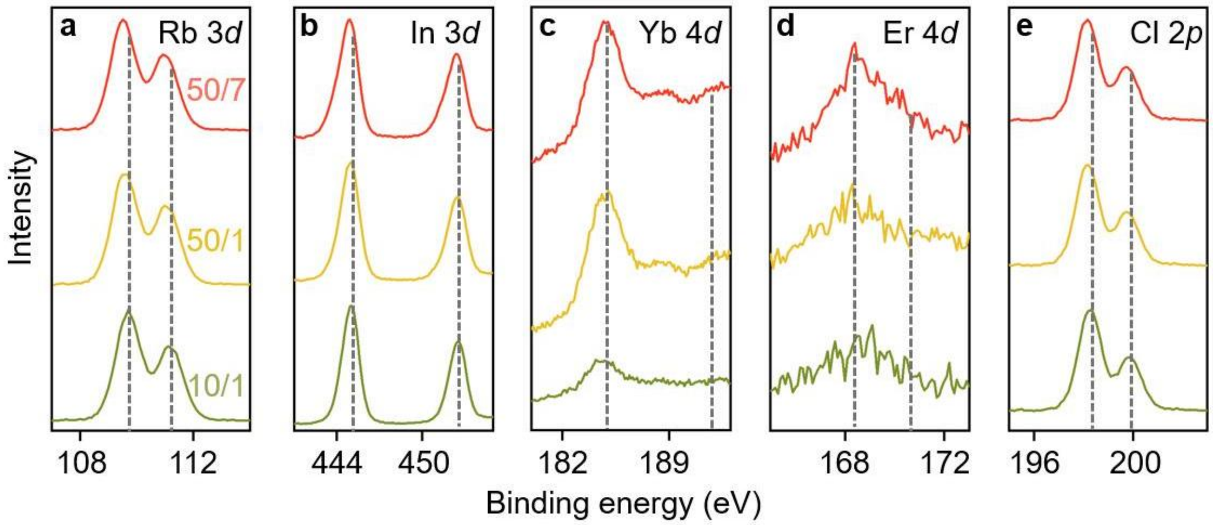


**Supplementary Figure 28**. XPS spectra of $Rb_3InCl_6$: 10%$Yb^{3+}$/1%$Er^{3+}$, $Rb_3InCl_6$: 50%$Yb^{3+}$/1%$Er^{3+}$, and $Rb_3InCl_6$: 50%$Yb^{3+}$/7%$Er^{3+}$ NCs. The spectra are shown over the energy regions typical for **a** Rb 3*d*, **b** In 3*d*, **c** Yb 4*d*, **d** Er 4*d*, and **e** Cl 2*p* peaks. The bimodal peaks at the binding energies of 109.7 and 111.1 eV, 445.0 and 452.3 eV, 184.7 and 192.2 eV, 169.1 eV and 170.4 eV, and 198.3 and 199.9 eV can be assigned to the $Rb^+$ $3d_{5/2}$ and $3d_{3/2}$, $In^{3+}$ $3d_{5/2}$ and $3d_{3/2}$, $Yb^{3+}$ $4d_{3/2}$ and $4d_{5/2}$, $Er^{3+}$ $4d_{3/2}$ and $4d_{5/2}$, and $Cl^-$ $2p_{1/2}$ and $2p_{3/2}$, respectively. The $Rb^+$ 3*d*, $Cl^-$ 2*p*, and $In^{3+}$ 3*d* peaks of the NCs shifted towards lower energies with the increasing $Yb^{3+}$ or $Er^{3+}$ concentration, ascribing to the strengthening of electron densities around $Rb^+$, $In^{3+}$, and $Cl^-$. This implies that $Yb^{3+}$ and $Er^{3+}$ ions replace the octahedral $In^{3+}$ site in the $Rb_3InCl_6$ lattice.

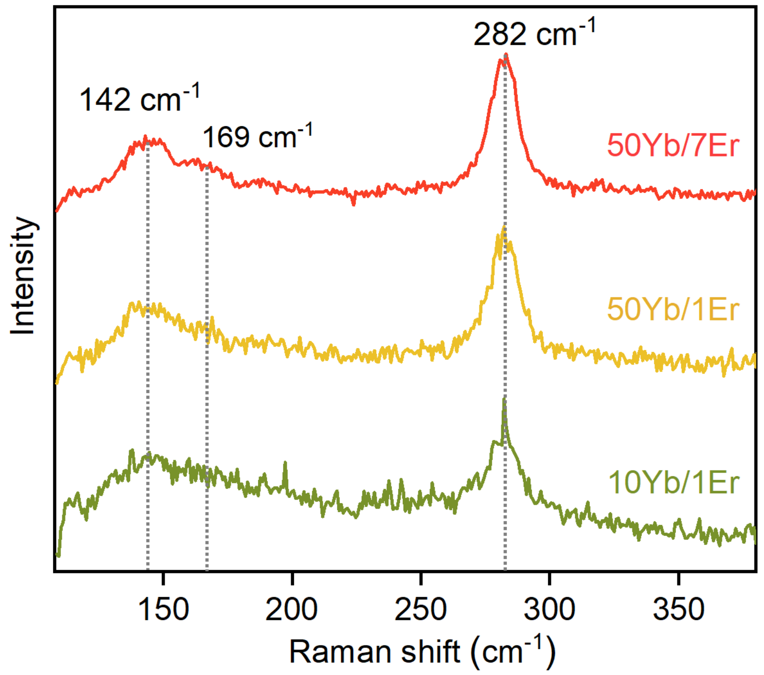


**Supplementary Figure 29**. Raman spectra of $Rb_3InCl_6$: 10%$Yb^{3+}$/1%$Er^{3+}$, $Rb_3InCl_6$: 50%$Yb^{3+}$/1%$Er^{3+}$, and $Rb_3InCl_6$: 50%$Yb^{3+}$/7%$Er^{3+}$ NCs. The vibrational peaks at 142, 169, and 282 $cm^{-1}$ can be ascribed to the $t_{2g}$, $e_g$, and $a_{1g}$ vibrational modes of the $[InCl_6]^{3-}$ octahedra, respectively. The Raman peaks of the NCs were broadened in comparison with those of the MCs, due to the influence of surface ligands and size effect of the NCs.

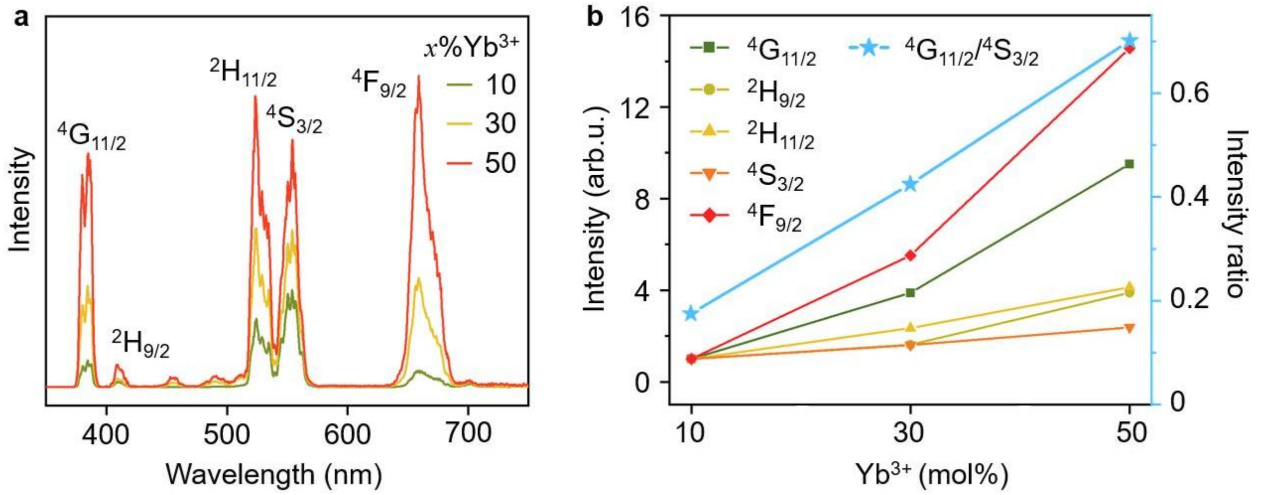


**Supplementary Figure 30**. **a** UCL spectra of $Rb_3InCl_6$: $x$%$Yb^{3+}$/1%$Er^{3+}$ NCs with different $Yb^{3+}$ concentrations under 980-nm excitation at a power density of 60 W $cm^{-2}$. **b** UCL intensity ratio of $^4G_{11/2}$/$^4S_{3/2}$ of $Er^{3+}$ and intensities of the upconverted emissions from $^4G_{11/2}$ (384 nm), $^2H_{9/2}$ (409 nm), $^2H_{11/2}$ (524 nm), $^4S_{3/2}$ (554 nm), and $^4F_{9/2}$ (659 nm) of $Er^{3+}$ in $Rb_3InCl_6$: $x$%$Yb^{3+}$/1%$Er^{3+}$ NCs as a function of the $Yb^{3+}$ concentration. The integrated UCL intensity of the NCs increased steadily with the increasing $Yb^{3+}$ concentration from 10 mol% to 50 mol%, indicating the absence of concentration quenching effect of $Yb^{3+}$ in $Rb_3InCl_6$: $x$%$Yb^{3+}$/1%$Er^{3+}$ NCs. Specifically, the intensity ratio of UV-to-green ($I_{384}/I_{554}$) of $Er^{3+}$ was remarkably enhanced from 0.175 (10 mol% of $Yb^{3+}$) to 0.702 (50 mol% of $Yb^{3+}$). These results are generally consistent with those observed in $Rb_3InCl_6$: $x$%$Yb^{3+}$/1%$Er^{3+}$ MCs.

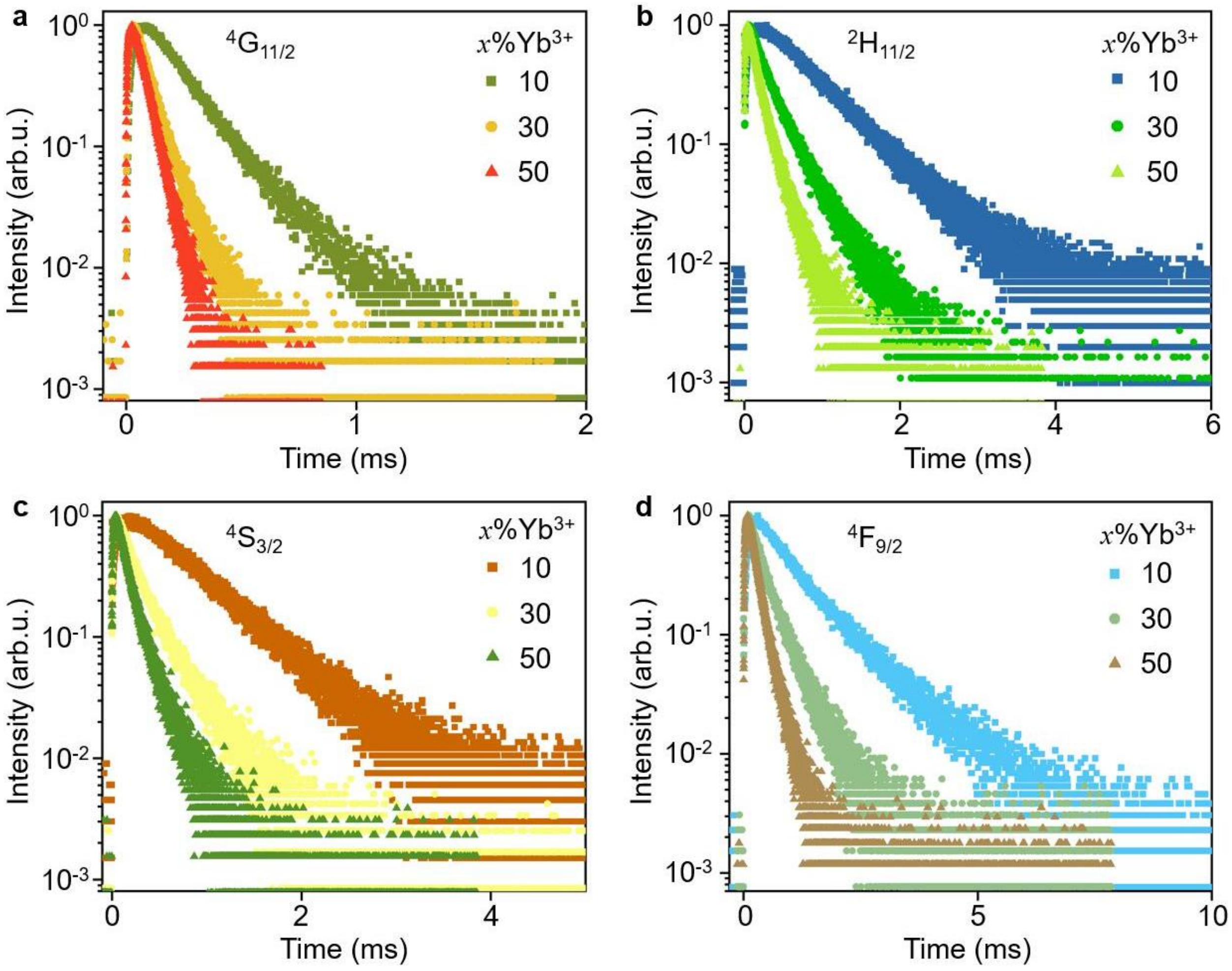


**Supplementary Figure 31**. UCL decay curves from **a** $^4G_{11/2}$ (384 nm), **b** $^2H_{11/2}$ (524 nm), **c** $^4S_{3/2}$ (554 nm), and **d** $^4F_{9/2}$ (659 nm) of $Er^{3+}$ in $Rb_3InCl_6$: $x$%$Yb^{3+}$/1%$Er^{3+}$ NCs with different $Yb^{3+}$ concentrations. The effective UCL lifetimes of $^4G_{11/2}$, $^2H_{11/2}$, $^4S_{3/2}$, and $^4F_{9/2}$ of $Er^{3+}$ were determined to decrease from 190, 662, 693, and 869 μs to 59, 157, 151, and 207 μs, respectively, as the $Yb^{3+}$ concentration increased from 10 to 50 mol% (see also Supplementary Table 13).

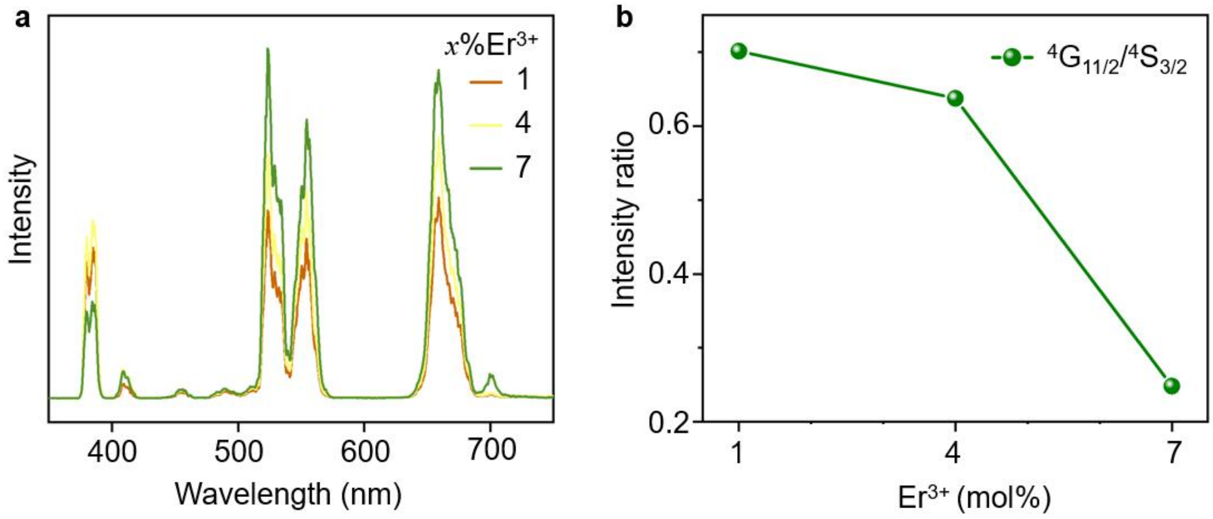


**Supplementary Figure 32**. **a** UCL spectra of $Rb_3InCl_6$: 50%$Yb^{3+}$/*y*%$Er^{3+}$ NCs with different $Er^{3+}$ concentrations under 980-nm excitation at a power density of 60 W $cm^{-2}$. **b** UCL intensity ratio of $^4G_{11/2}$/$^4S_{3/2}$ of $Er^{3+}$ ($I_{384}/I_{554}$) in $Rb_3InCl_6$: 50%$Yb^{3+}$/*y*%$Er^{3+}$ NCs as a function of the $Er^{3+}$ concentration, showing decreased $I_{384}/I_{554}$ with the increasing $Er^{3+}$ concentration.

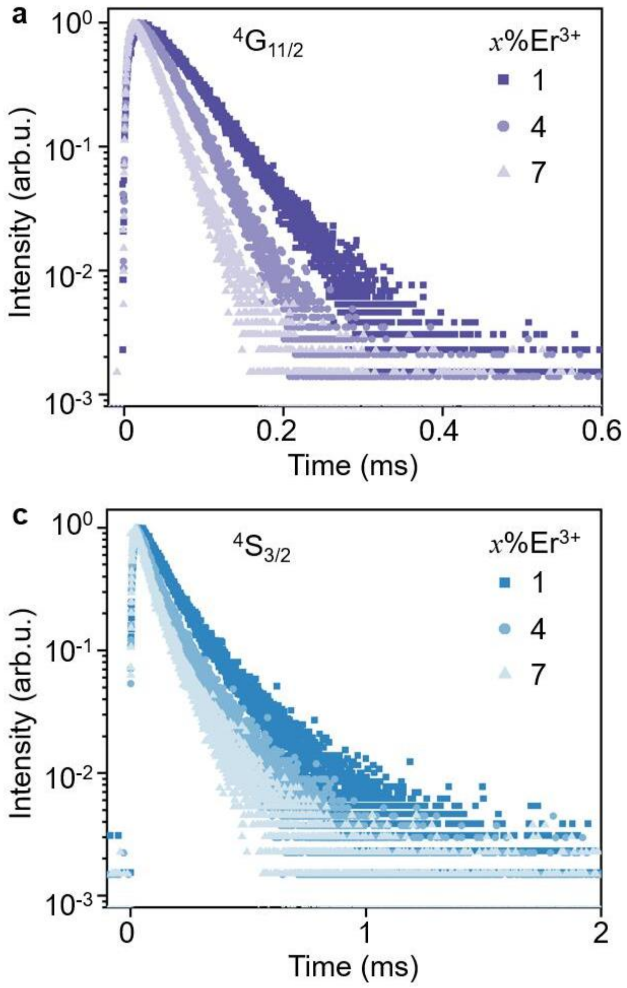


**Supplementary Figure 33**. UCL decay curves from **a** $^4G_{11/2}$ (384 nm), **b** $^2H_{11/2}$ (524 nm), **c** $^4S_{3/2}$ (554 nm), and **d** $^4F_{9/2}$ (659 nm) of $Er^{3+}$ in $Rb_3InCl_6$: 50%$Yb^{3+}$/*y*%$Er^{3+}$ NCs with different $Er^{3+}$ concentrations. The effective UCL lifetimes of $^4G_{11/2}$, $^2H_{11/2}$, $^4S_{3/2}$, and $^4F_{9/2}$ of $Er^{3+}$ were determined to decrease from 59, 157, 151, and 207 μs to 33, 87, 78, and 167 μs, respectively, as the $Er^{3+}$ concentration increased from 1 to 7 mol% (see also Supplementary Table 14).

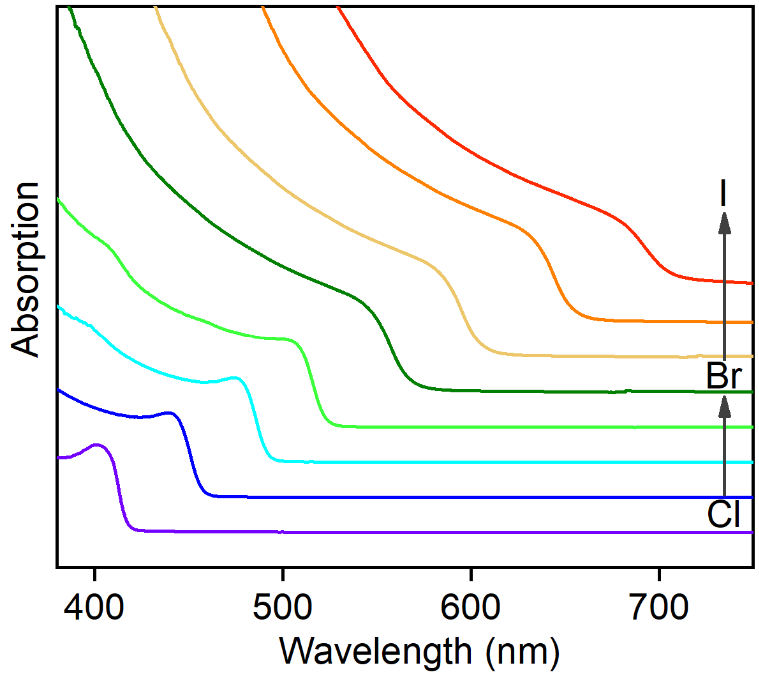


**Supplementary Figure 34**. Optical absorption spectra of $CsPbX_3$ PeNCs with different halide compositions, derived from anion exchange based on the mixtures of RIC/DBM/$CsPbCl_3$ and RIC/IDP/$CsPbBr_3$ upon 980-nm NIR laser exposure.

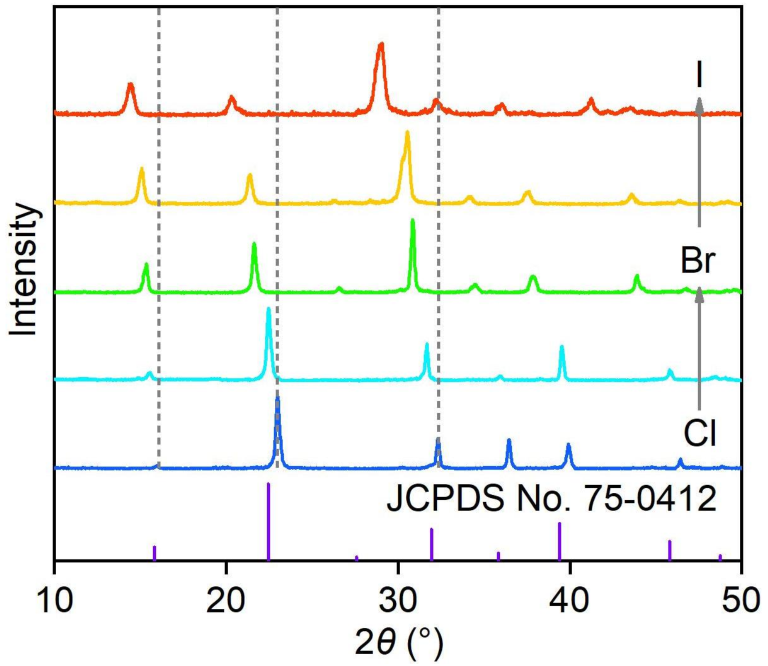


**Supplementary Figure 35**. XRD patterns of $CsPbX_3$ PeNCs with different halide compositions, derived from anion exchange based on the mixtures of RIC/DBM/$CsPbCl_3$ and RIC/IDP/$CsPbBr_3$ upon 980-nm NIR laser exposure. The bottom line represents the standard XRD patterns of cubic $CsPbBr_3$ (JCPDS No. 75-0412). All diffraction peaks of the PeNCs can be well indexed into cubic $CsPbX_3$, indicating high crystallinity and phase purity of the resulting PeNCs.

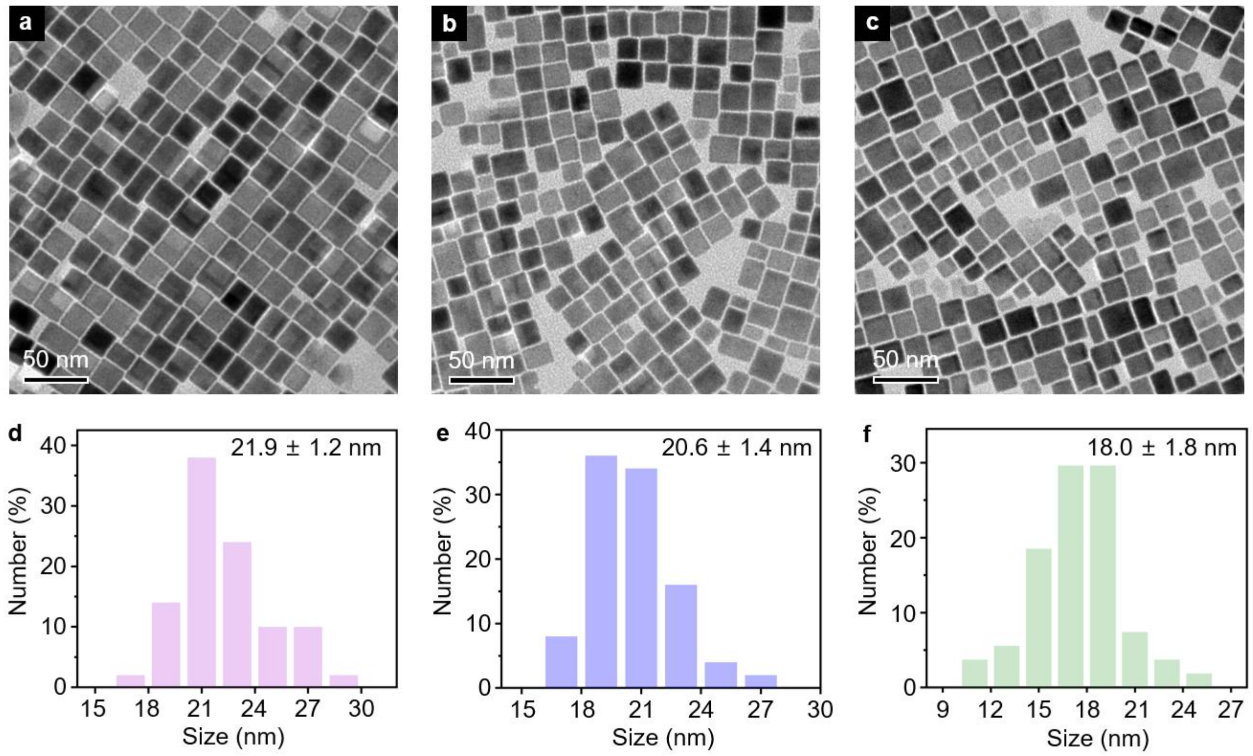


**Supplementary Figure 36**. **a-c** TEM images and **d-f** size distribution histograms of $CsPbCl_3$, $CsPbBr_3$, and $CsPbI_3$ PeNCs, derived from anion exchange based on the mixtures of RIC/DBM/$CsPbCl_3$ and RIC/IDP/$CsPbBr_3$ upon 980-nm NIR laser exposure.

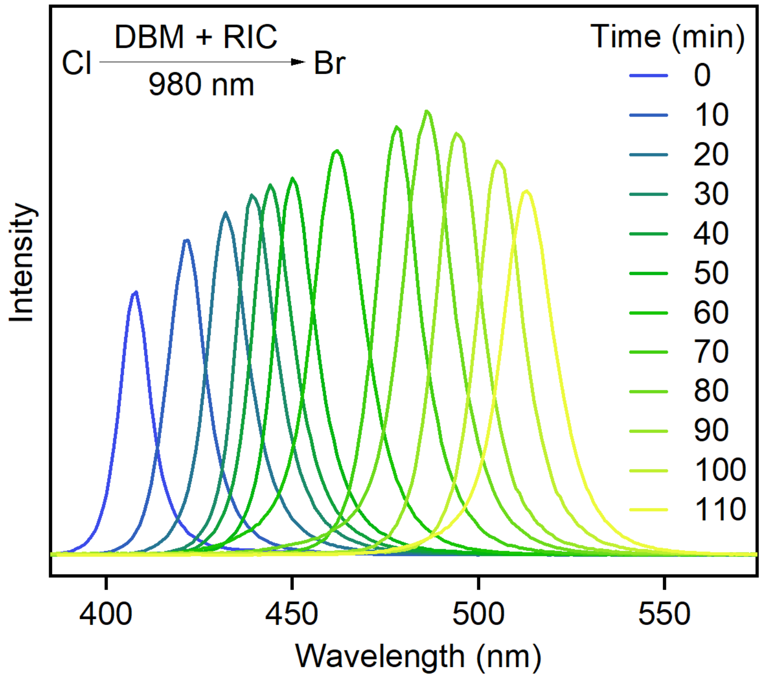


**Supplementary Figure 37**. Time-dependent PL emission spectra ($\lambda_{ex}$ = 365 nm) for $CsPbCl_3$ → $CsPbBr_3$ in RIC/DBM/$CsPbCl_3$ upon 980-nm irradiation at a power density of 60 W $cm^{-2}$.

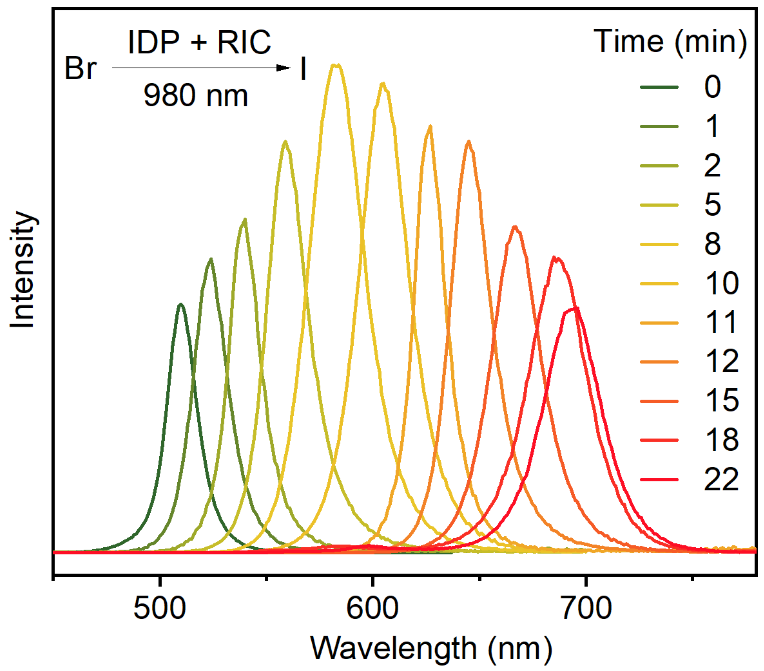


**Supplementary Figure 38**. Time-dependent PL emission spectra ($\lambda_{ex}$ = 365 nm) for $CsPbBr_3 \rightarrow CsPbI_3$ in RIC/IDP/$CsPbBr_3$ upon 980-nm irradiation at a power density of 60 W $cm^{-2}$.

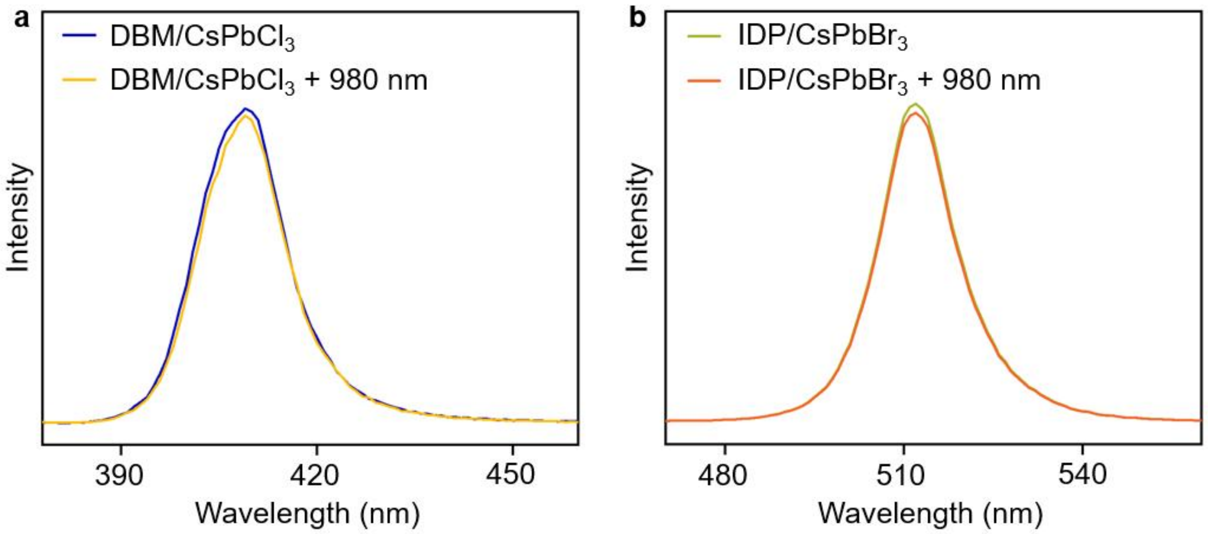


**Supplementary Figure 39**. PL emission spectra ($\lambda_{ex}$ = 365 nm) of the mixtures of **a** DBM/$CsPbCl_3$ and **b** IDP/$CsPbBr_3$ before and after irradiation with the 980-nm laser at a power density of 60 W $cm^{-2}$ for 3 hours.

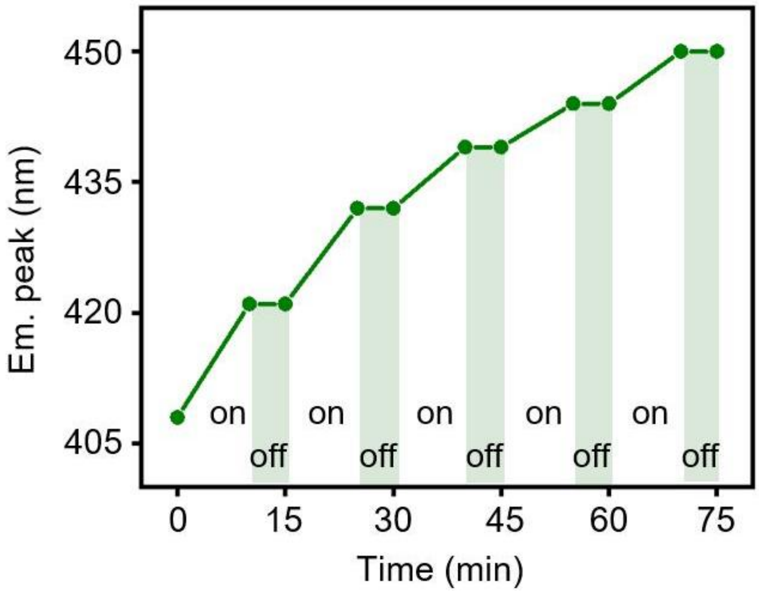


**Supplementary Figure 40**. PL emission peak shift for $CsPbCl_3$ → $CsPbBr_3$ in RIC/DBM/$CsPbCl_3$ under the "on" and "off" period of 980-nm laser irradiation.

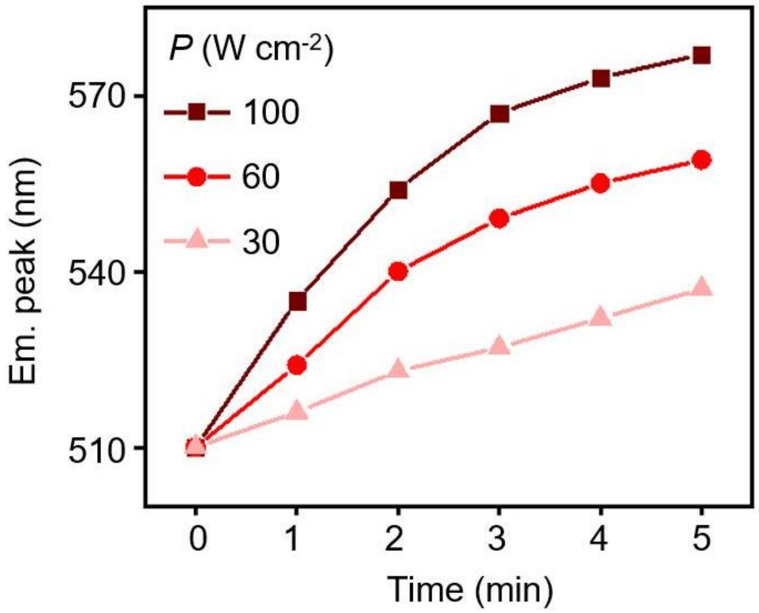


**Supplementary Figure 41**. Time dependence of the PL emission peak shift for $CsPbBr_3$ → $CsPbI_3$ in RIC/IDP/$CsPbBr_3$ under 980-nm laser irradiation at different power densities.

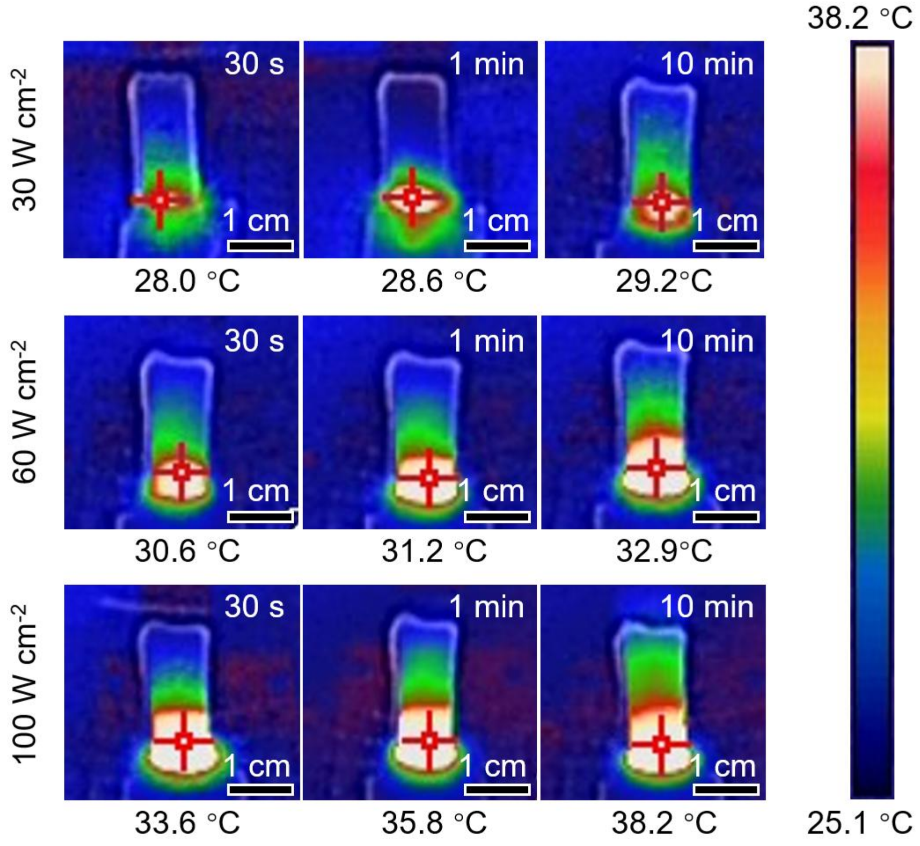


**Supplementary Figure 42**. Thermographs of the RIC/IDP/$CsPbBr_3$ solution upon 980-nm excitation at power densities of 30, 60, and 100 W cm$^{-2}$ for 10 min, recorded by an infrared thermographic camera. The laser-induced temperature rise is well below the boiling temperature (≈80.7 °C) of the cyclohexane solution. Such laser-induced heating effect may also contribute to the enhanced anion exchange rate due to the increased motion of the anions with the temperature rise.

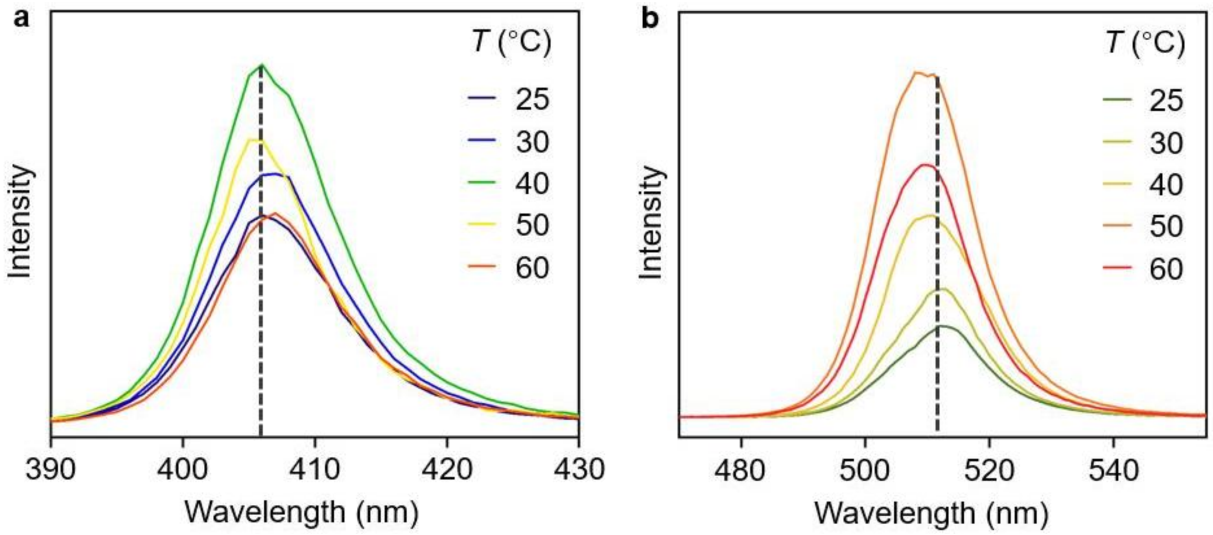


**Supplementary Figure 43**. PL emission spectra ($\lambda_{ex}$ = 365 nm) of **(a)** the RIC/DBM/$CsPbCl_3$ and **(b)** RIC/IDP/$CsPbBr_3$ solutions after heating in the dark for 10 minutes at different temperatures. The emission peak positions of the RIC/DBM/$CsPbCl_3$ and RIC/IDP/$CsPbBr_3$ solutions remained nearly unchanged with varying temperatures, despite of the different intensities. These observations demonstrate unambiguously that the local heating effect of laser (60 W $cm^{-2}$, 32.9 °C) cannot trigger the anion exchange of PeNCs in the mixed solutions.

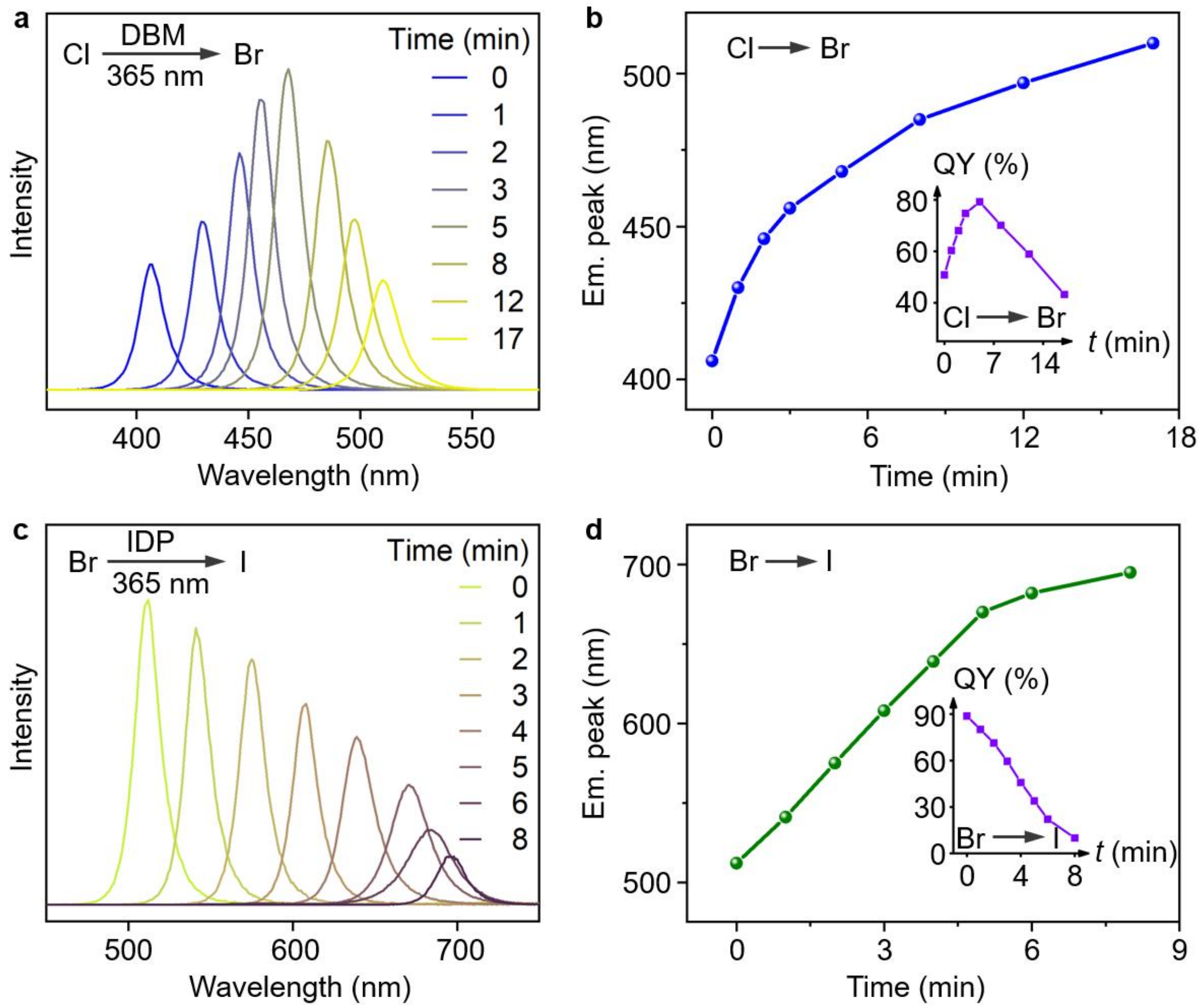


**Supplementary Figure 44**. Time-dependent PL emission spectra ($\lambda_{ex}$ = 365 nm) for **a** $CsPbCl_3$ → $CsPbBr_3$ in DBM/$CsPbCl_3$ and **c** $CsPbBr_3$ → $CsPbI_3$ in IDP/$CsPbBr_3$ upon 365-nm UV-LED (3 W) irradiation. Time dependence of the PL emission peaks ($\lambda_{ex}$ = 365 nm) for **b** $CsPbCl_3$ → $CsPbBr_3$ in DBM/$CsPbCl_3$ and **d** $CsPbBr_3$ → $CsPbI_3$ in IDP/$CsPbBr_3$ upon 365-nm UV-LED (3 W) irradiation. The insets in **b** and **d** show the corresponding PLQYs of PeNCs. The reaction times required for completing the anion exchange from $CsPbCl_3$ to $CsPbBr_3$ and from $CsPbBr_3$ to $CsPbI_3$ were 17 and 8 minutes, respectively, under UV illumination, much shorter than those upon NIR irradiation using $Rb_3InCl_6$: $Yb^{3+}$/$Er^{3+}$ (110 and 22 minutes). However, the PLQYs of PeNCs after UV-triggered anion exchange decreased significantly from 50.8% for $CsPbCl_3$ to 43.2% for $CsPbBr_3$ and from 88.7% for $CsPbBr_3$ to 10.0% for $CsPbI_3$, due to the serious photodamage of PeNCs especially $CsPbI_3$ upon prolonged UV exposure.

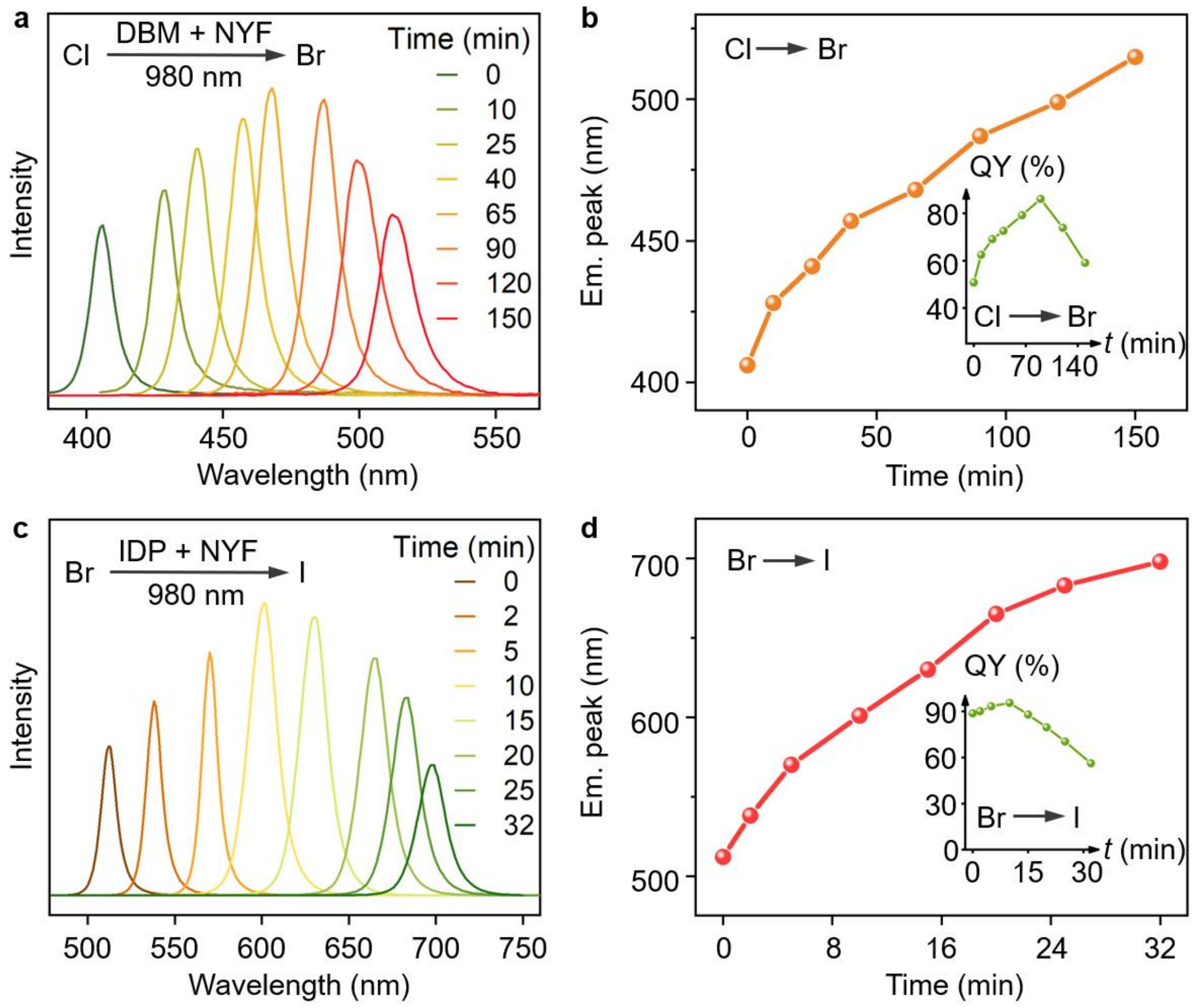


**Supplementary Figure 45**. Time-dependent PL emission spectra ($\lambda_{ex}$ = 365 nm) for **a** $CsPbCl_3$ → $CsPbBr_3$ in NYF/DBM/$CsPbCl_3$ and **c** $CsPbBr_3$ → $CsPbI_3$ in NYF/IDP/$CsPbBr_3$ upon 980-nm laser irradiation at a power density of 60 W/cm$^{-2}$. Time dependence of the PL emission peaks ($\lambda_{ex}$ = 365 nm) for **b** $CsPbCl_3$ → $CsPbBr_3$ in NYF/DBM/$CsPbCl_3$ and **d** $CsPbBr_3$ → $CsPbI_3$ in NYF/IDP/$CsPbBr_3$ upon 980-nm laser irradiation at a power density of 60 W cm$^{-2}$. The insets in **b** and **d** show the corresponding PLQYs of PeNCs. The reaction times required for completing the anion exchange from $CsPbCl_3$ to $CsPbBr_3$ and from $CsPbBr_3$ to $CsPbI_3$ were 150 and 32 minutes, respectively, by using $NaYF_4$: $Yb^{3+}/Er^{3+}$ as the NIR-to-UV transducer, which are longer than those based on $Rb_3InCl_6$: $Yb^{3+}/Er^{3+}$ (110 and 22 minutes). The PLQYs of PeNCs obtained via the NIR-triggered anion exchange using $NaYF_4$: $Yb^{3+}/Er^{3+}$ (50.8-95.5%) were somewhat lower than those using $Rb_3InCl_6$: $Yb^{3+}/Er^{3+}$ (52.3–98.1%), probably due to the prolonged NIR illumination.

**Supplementary References**

1 Yokota, M and Tanimoto, O. Effects of Diffusion on Energy Transfer by Resonance. *J. Phys. Soc. Jpn.* **22**, 779-784 (1967).
